# Cytometry in the Short-Wave Infrared

**DOI:** 10.1021/acsnano.4c04345

**Published:** 2024-07-08

**Authors:** Te-I Liu, Jhih-Shan Wang, Ai-Phuong Nguyen, Marco Raabe, Carlos Jose Quiroz Reyes, Chih-Hsin Lin, Ching-Wei Lin

**Affiliations:** † Institute of Atomic and Molecular Sciences, Academia Sinica, Taipei City 106319, Taiwan; ‡ Department of Materials Science and Engineering, National Taiwan University, Taipei City 106319, Taiwan; § Department of Physics, University of Stuttgart, Stuttgart 70174, Germany; ∥ Department of Chemistry, National Tsing Hua University, Hsinchu 300044, Taiwan; ⊥ International Ph.D. Program in Biomedical Engineering, Taipei Medical University, New Taipei City 235603, Taiwan; # Graduate Institute of Nanomedicine and Medical Engineering, Taipei Medical University, New Taipei City 235603, Taiwan

**Keywords:** flow cytometry, image cytometry, single-wall carbon nanotubes, NIR-II window, reactive oxygen species, antioxidant

## Abstract

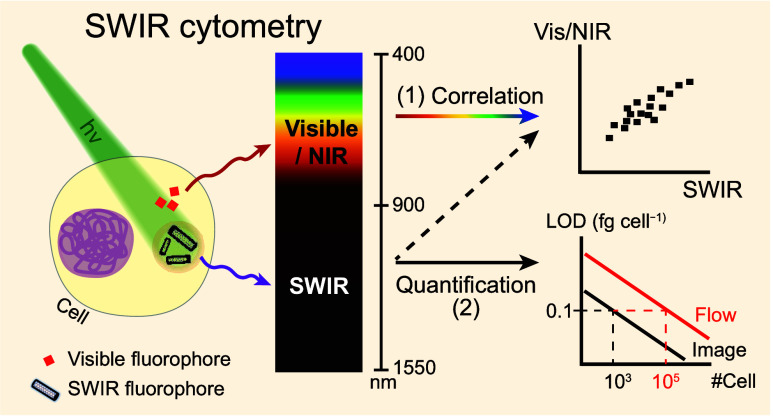

Cytometry plays a crucial role in characterizing cell properties, but its restricted optical window (400–850 nm) limits the number of stained fluorophores that can be detected simultaneously and hampers the study and utilization of short-wave infrared (SWIR; 900–1700 nm) fluorophores in cells. Here we introduce two SWIR-based methods to address these limitations: SWIR flow cytometry and SWIR image cytometry. We develop a quantification protocol for deducing cellular fluorophore mass. Both systems achieve a limit of detection of ∼0.1 fg cell^–1^ within a 30 min experimental time frame, using individualized, high-purity (6,5) single-wall carbon nanotubes as a model fluorophore and macrophage-like RAW264.7 as a model cell line. This high-sensitivity feature reveals that low-dose (6,5) serves as an antioxidant, and cell morphology and oxidative stress dose-dependently correlate with (6,5) uptake. Our SWIR cytometry holds immediate applicability for existing SWIR fluorophores and offers a solution to the issue of spectral overlapping in conventional cytometry.

Cytometry, a crucial technique in biological and medical research, facilitates quantitative analyses of physical, chemical, and biological characteristics of cells, including parameters such as number, size, morphology, and biomarker expression.^1^ Unlike ensemble-based measurements such as those conducted with a plate reader, cytometry possesses the distinct advantage of quantifying specific targets, such as intracellular proteins, signaling molecules or surface markers, on the single-cell basis.^2,3^ This provides immediate capabilities of identifying correlations between two different cellular factors through statistical analysis.^4^ Traditional cytometry detects the fluorophore emission in the visible to near-infrared (NIR), or 400–850 nm, satisfying most of the developed biomarker labeling toolkits in the market for cell characterizations.^5^

Recent developments in fluorophores that fluoresce at wavelengths longer than 900 nm, in the short-wave infrared (SWIR) range (900–1700 nm), for deep tissue in vivo imaging have gathered attention for potential clinical translation.^6^ For instance, inorganic nanoparticles like single-wall carbon nanotubes (SWCNTs),^7,8^ quantum dots,^9^ rare-earth-doped downconversion nanoparticles,^10^ and organic dyes, such as donor–acceptor–donor-structured molecules^11^ and polymer dots,^12^ have been utilized in in vivo diagnostics and imaging applications such as angiography,^13,14^ cancer nodule targeting,^8,15,16^ in vivo sensing,^17−19^ image-guided surgery,^20,21^ and single-cell tracking.^22,23^ SWIR light is preferred in these applications due to its lower tissue scattering and autofluorescence.^24,25^ Despite their promising performance, understanding the underlying cellular pathways and subsequent fluorophore metabolism is crucial. The lack of a SWIR-based cytometric tool hinders related research, as current visible-range cytometry cannot be directly applied. Additionally, the most advanced visible cytometry using whole spectral imaging with sophisticated spectral overlap (spillover) compensations has reached its maximum number of detection channels, typically up to ∼20–50 colors.^26−28^ However, there is a growing need for the simultaneous staining of even more colors, and the unmixing of spectral overlaps becomes extremely challenging, especially with highly overlapped emissions. Expansion of the detection window toward longer wavelengths,^29,30^ as offered by SWIR, presents a vivid solution.

In this work, we introduce two types of SWIR cytometries: flow cytometry measures cell signals using a single-element detector, while image cytometry using a two-dimensional camera. Utilizing highly individualized and purified (6,5)-SWCNTs as a model fluorophore and macrophage-like RAW264.7 cells as a model cell line, we develop standard protocols for fluorophore quantification, deduce and compare the limits of detection (LOD) of both systems, determine the level of signal-to-noise-ratio (SNR), and examine the importance of spillover compensations in the SWIR range. Finally, we conduct a case study to investigate the correlations between the amount of (6,5) uptake and the level of intracellular reactive oxygen species.

## RESULTS and DISCUSSION

### SWIR Fluorophore and SWIR Cytometers Used

**Figure 1:**
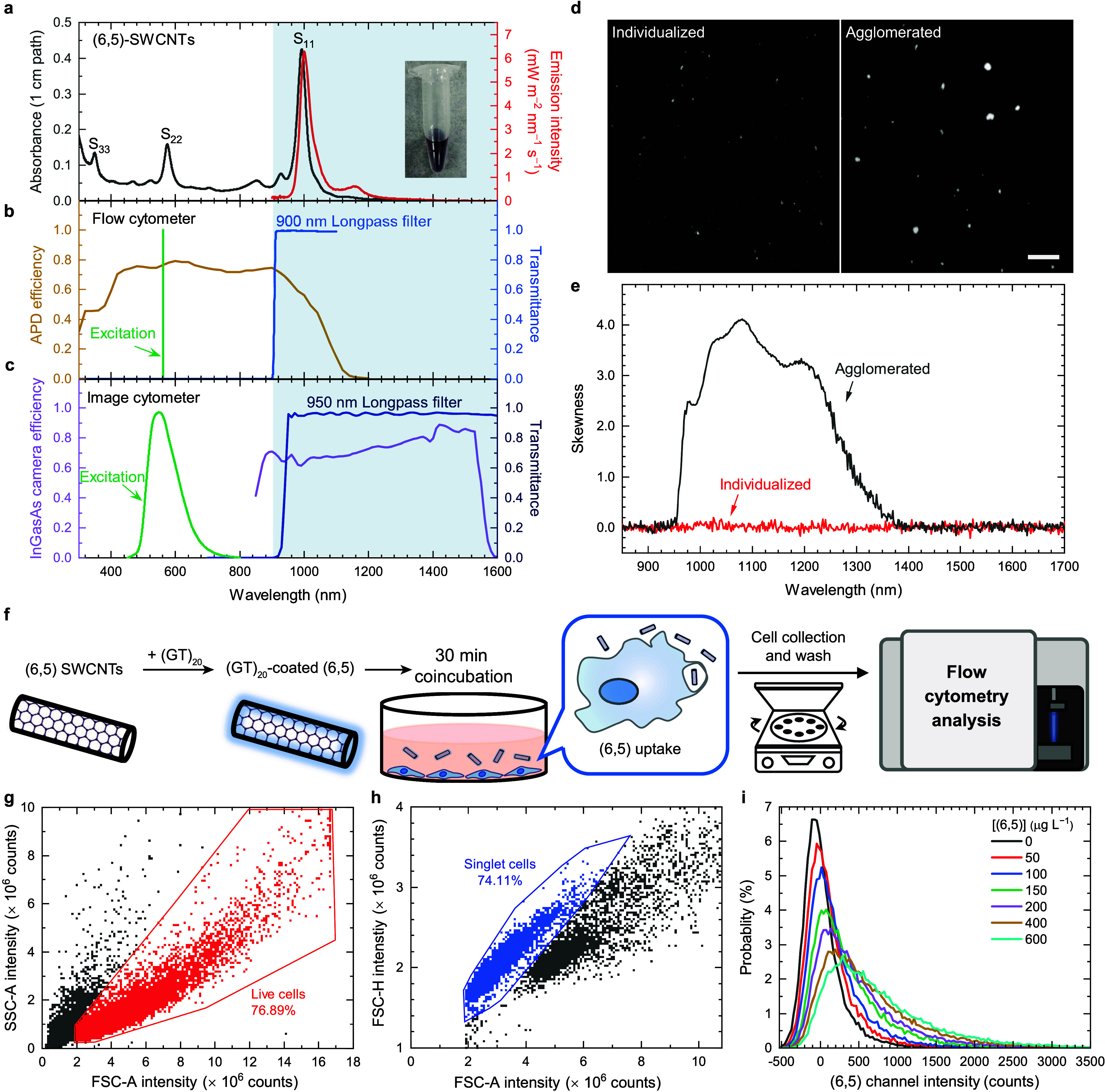
Characterization of the SWIR fluorophore, spectral information on the SWIR cytometers, and fluorescence analysis of the cellular (6,5) using SWIR flow cytometry. (a) Absorption (black line) and fluorescence (red line) spectra of the (GT)_20_-coated (6,5). Inset picture shows a photo of the (6,5) sample. (b) APD efficiency spectrum, transmittance spectrum of the 900 nm long-pass filter, and excitation wavelength of the flow cytometer. An APD quantum efficiency spectrum is regenerated from the datasheet of Beckman CytoFLEX. (c) InGaAs camera efficiency spectrum, transmittance spectrum of the 950 nm long-pass filter, and excitation wavelength of the image cytometer. InGaAs quantum efficiency spectrum comes from the datasheet of Princeton Instruments NIRvana LN. The illumination spectra of the LED is regenerated from the document of Thorlabs Chrolis High-Power LED Sources. (d) SWIR image and (e) skewness spectra of the (6,5) in phosphate buffer. The length of the scale bar equals 100 μm. (f) Illustration of the experimental procedure. The (6,5) is treated for 30 min. (g) Scatter plot of SSC (side scatter) vs FSC (forward scatter). Live cells are gated in red. (h) Scatter plot of FSC-H vs FCS-A. Singlet cells are gated in blue. (i) Probability distributions with respect to emission intensities of (6,5) in singlet cells (gated population). The bin width of the distributions is set to 30 counts. Seven samples with various (6,5) concentrations are measured. A cell number of ∼50,000 is presented.

Semiconducting SWCNTs emit sharp SWIR fluorescence^31^ and show minimal photobleaching under strong illumination,^32^ making them ideal model fluorophores for developing SWIR fluorescence-related instruments. In Figure 1a, the absorption spectrum of high-purity (6,5) reveals strong Van Hove transitions, including S_11_ at 992 nm, S_22_ at 574 nm, and S_33_ at 350 nm. Efficient excitation is achieved using a commercially available 561 nm laser. Small-diameter SWCNTs with diameters of ∼1 nm have S_22_ transitions in the visible range, making them immediately applicable to most flow cytometers. However, the emission peak of (6,5) at 999 nm falls outside the detection region of commonly used photomultiplier tube detectors.^33^ To attain reasonable detection sensitivity near the 1000 nm region, we selected a flow cytometer equipped with avalanche photodiodes (APD) featuring enhanced SWIR detection efficiency up to 1100 nm (see Figure 1b). We further installed a 900 nm long-pass filter in the emission path to exclusively collect the (6,5) emission. In parallel, we employed a deeply cooled indium gallium arsenide (InGaAs) camera with a 950 nm long-pass filter and a green light-emitting diode (LED) to construct our SWIR image cytometer. The spectral information is shown in Figure 1c.

To enable quantitative analysis of the SWIR emissive (6,5), it is crucial to ensure the individualization of the nanotubes, minimizing biased intensities from fluorescence quenching and peak broadening. We achieve this by centrifuging a surfactant-dispersed (GT)_20_-coated (6,5) sample, followed by collecting its supernatant and pellet as representatives for individualized and agglomerated samples, respectively. Far-field images facilitate the identification of particles much larger than the diffraction limit, which is ∼642 nm for the SWIR fluorescence under Rayleigh criterion. As shown in Figure 1d, individualized particles appear as small dots with one to few pixel sizes, while larger agglomerates are evident in the right panel. We can, therefore, affirm the absence of agglomerates larger than the diffraction limit in the well-suspended sample. To further investigate the presence of nanotube bundles smaller than ∼642 nm, we conducted skewness analysis using the variance spectrometry.^34^Figure 1e shows the skewness spectra of both agglomerated and nonagglomerated samples. The full spectral analysis, rather than a single-pixel approach, clearly indicates a noise level at ∼0.1. The agglomerated sample exhibits high (6,5) intensity skewness in the range of 950–1400 nm, whereas the nonagglomerated sample shows no observable skewness signal, suggesting no small bundles in the sample. Therefore, we utilized the well-individualized (6,5) for the subsequent experiments.

### SWIR Emission Measured by Cytometry

To detect SWIR signals from flowing single cells, we first treat RAW264.7 cells with our model SWIR fluorophore, (6,5)-SWCNTs. Macrophage-like cells are chosen for their capability to engulf foreign objects and are important for the toxicological studies of nanomedicine. Figure 1f outlines our protocol for preparing cells treated with varying amount of (GT)_20_-coated (6,5) (hereinafter abbreviated as (6,5)). After (6,5) treatment, cells with different amount of (6,5) are dislodged and collected for measurements using flow cytometry. First, live RAW macrophages are gated, excluding all cell debris and large cell clumps (see Figure 1g). Second, we gate the smaller forward scatter-area (FSC-A) population to obtain singlet-cell signals shown in blue (Figure 1h). It is essential to note that the individualized (6,5) particles or even their loose agglomerates do not contribute to the analyzed scatter signals because their nanometer to submicron size is beyond the gated region for singlet cells. Figure 1i shows probability functions of (6,5) fluorescence intensities from the gated singlet live cells at seven doses of (6,5) concentrations ranging from 0 to 600 μg L^–1^ (see Figure S7a for nongated results). The spreads of the intensities from nonstained cells predominately arise from the system noise, rather than autofluorescence (see Discussion S7 and Figure S9). A clear monotonic sublinear increase of the mean fluorescence intensity (MFI) with increasing (6,5) dose concentration is observed, indicating a larger amount of (6,5) per cell with higher (6,5) concentration treatment. The larger fwhm at larger MFI represents varying amount of (6,5) uptake among cells, broadening the distribution. The feasibility of SWIR image cytometry using an InGaAs camera is not shown, as it is well-known that the SWIR fluorescence microscope system allows the detection of (6,5) down to single particle level.^35−37^

### (6,5) Quantification Protocol for SWIR Cytometry

**Figure 2:**
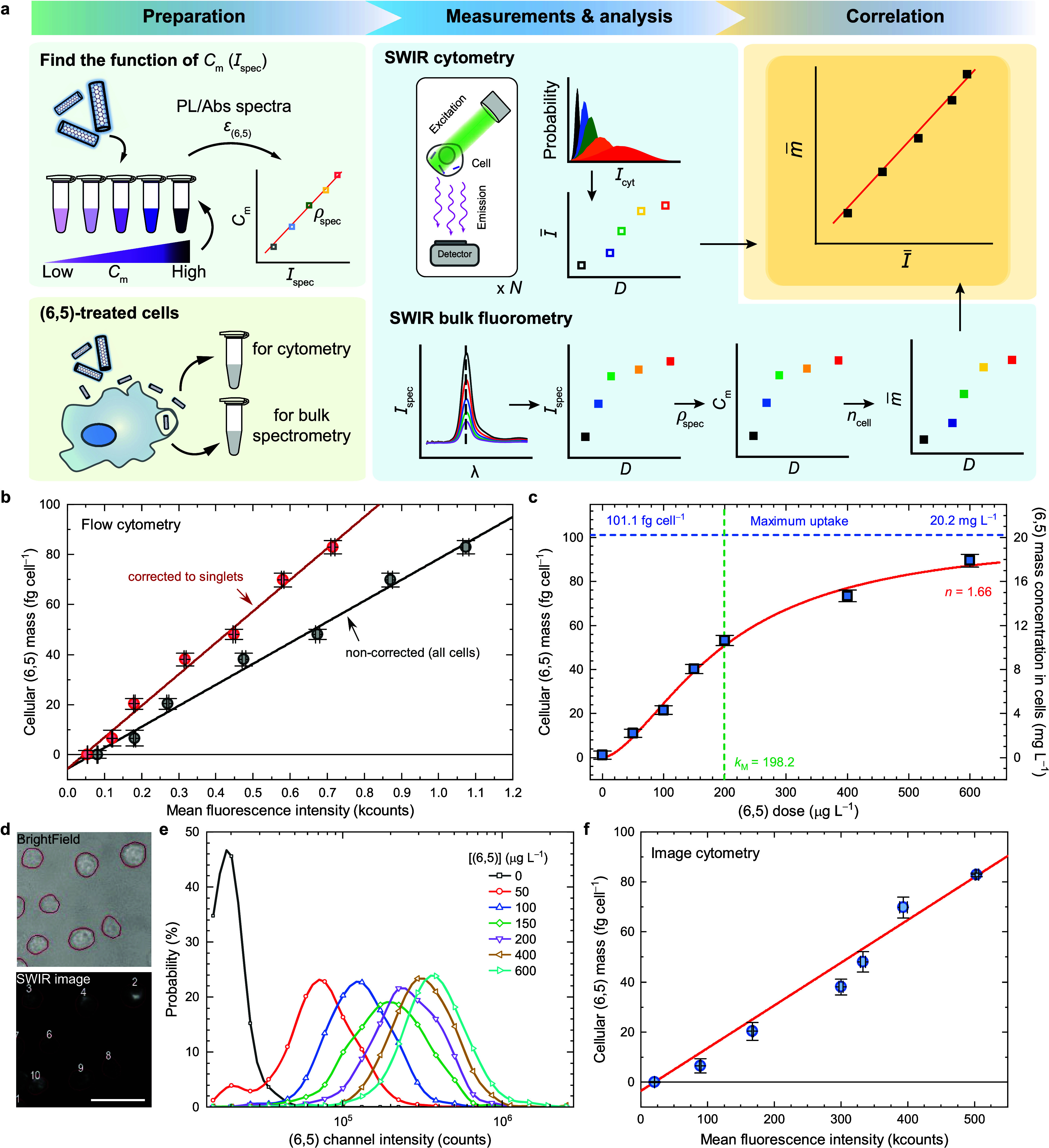
Quantification protocol and resulting parameters for (6,5) in RAW macrophages using SWIR cytometers. (a) *C*_*m*_, (6,5) mass concentration of the sample for incubation. *I*_spec_, (6,5) fluorescence intensity measured from the SWIR spectrometer. *ρ*_spec_, the (6,5) mass concentration *C*_*m*_ divided by the (6,5) fluorescence intensity *I*_spec_. *n*_cell_, the specific cell number. λ, emission wavelength. *D*, dose of (6,5) for incubation. *I*_cyt_, fluorescence intensity of the (6,5) channel measured in the cytometry. *I̅*, mean of *I*_cyt_. *m̅*, average mass of the (6,5) in a cell. The *m̅* as a function of *I̅*, *m̅*(*I̅*), is the *cellular fluorophore mass function*. (b) The cellular fluorophore mass function *m̅*(*I̅*) measured from the (6,5) channel in the SWIR flow cytometer. The black line represents the linear fit to the result from all cell events, and the red line represents the linear fit to the same data set with additional correction (a multiplication of *β* = 1.5) for cell clusters. Absolute (6,5) concentration of the following experiments will be deduced from the measured MFI. (c) The cellular (6,5) mass *m̅* with respect to the treated (6,5) mass concentration *D*. The red curve indicates data fitted with Hill equation. (d) Microscope images of the cells treated with (6,5)-SWCNTs for 30 min. Machine-learning based segmentation is performed and is represented as red outlines around cells. Scale bar represents 100 μm. (e) (6,5) channel intensity histograms *P*(*I*_image_), which are binned at a width of *P*(*I*_image_) = 1.3, within the red outlines from the singlet cells. (f) Cellular (6,5) mass as a function of the MFI from the distribution shown in Figure S16a. The error bars represent SD from triplicate samples.

The measured fluorescence intensity from a cytometer can be used to quantify the amount of the fluorophores when calibrated. Figure 2a illustrates our strategy for quantifying the (6,5) mass in a cell from the measured MFI. The procedure contains three steps: preparation, measurements and analysis, and correlation. In the preparation step, we first use a spectrophotometer and a SWIR fluorometer to obtain absolute (6,5) concentrations and their corresponding fluorescence intensities. Eight samples with various (6,5) concentrations are measured. A linear relation between (6,5) mass concentration *C*_*m*_ and (6,5) fluorescence intensity *I*_spec_ can be obtained with a slope ρ_spec_, and the *C*_*m*_ of any ensemble sample can then be interpolated using the measured *I*_spec_ (*C*_*m*_ = ρ_spec_*I*_spec_, see Figure S12d). This is an essential step to achieve practical and accurate measurements because of the following two main reasons: first, our system is capable of measuring decent fluorescence signal from low-volume samples, which is true in this case because only limited amount of (6,5) can be collected from the cells; second, the remained cell residues in the samples give background absorption and additional scattering, causing overestimated and nonlinear SWCNT absorption values (see Figure S10). Therefore, the fluorescence measurements ensure almost no extra signal deviation from the cell residues in the SWIR range, and the ultrashort light path through the sample guarantees a calibratable linear response. The second part of the preparation step is to incubate the RAW macrophages and treat them with various (6,5) concentrations. After (6,5) treatment, the cells are collected and separated into three aliquots, one for SWIR bulk fluorometry, another for SWIR flow cytometry, and the remaining aliquot for SWIR image cytometry, for the following measurement and analysis step.

In the second step, (6,5) fluorescence is measured and analyzed using both cytometry and bulk fluorometry. For SWIR cytometry, the (6,5) fluorescence distributions from all cell events are converted into the MFI of the (6,5) *I̅* with respect to their corresponding doses *D* (see Discussion S6). For SWIR bulk fluorometry, the SWIR fluorescence spectra of the samples are measured, giving a relation of peak intensity *I*_spec_ as a function of dose *D*. The *I*_spec_ can be converted to the (6,5) mass concentration *C*_*m*_ using the *ρ*_spec_ obtained from the first step, followed by the deduction of the cellular (6,5) mass *m̅* using the measured specific cell number *n*_cell_(*D*), or the cell number per unit volume. Detailed measurement results for *n*_cell_(*D*) deduction are listed in Table S3. Hence, the resulting equation for (6,5) mass per cell *m̅* as a function of the dose *D* is
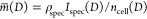
1

In the last step, we correlate the cellular (6,5) mass *m̅* from bulk fluorometry and the MFI *I̅* from cytometry, giving a *cellular fluorophore mass function m̅* (*I̅*).
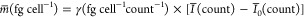
2where γ is the cellular (6,5) mass per unit of *I̅*, or the inverse brightness of an emitter, and *I̅*_0_ is the MFI without (6,5). We take the whole cell population (nongated) to correlate the results obtained from bulk measurements. The cellular (6,5) mass *m̅* can then be deduced directly from the measured (6,5) MFI *I̅* with correction using eq 2. Even though the deduction of the γ is performed using nongated data, cellular fluorophore mass function can be applied to any cellular conditions by multiplying a correction factor β, which is the all-cell to gated (e.g., singlet-cell) event number ratio, to the original γ (see Discussion S12). We also note that the γ can vary if the quantum yield of the fluorophore changes, a phenomenon that occurs frequently in many fluorescent nanoparticles.

### Quantification of Cellular (6,5) in SWIR Flow Cytometry

We performed the calibration protocol described in the previous section and obtained the results for the cellular fluorophore mass function (eq 2) shown in Figure 2b and Table 1. The 68-count *I̅*_0_ represents a much lower autofluorescence in the (6,5) channel than in other visible channels (see Figure S11). Figure 2c reveals the cellular (6,5) mass *m̅* with respect to the (6,5) dose *D* for the gated singlet-cells. This relation can be fitted well using the following Hill equation, which has been used to explain the nanoparticle uptake behaviors:^38,39^
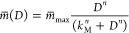
3

**Table 1:** Fitted Parameter Values Using Eq 1, Eq 2, and Eq 5 for Flow and Image Cytometers^a^

Parameter	Bulk fluorometry	Flow cytometry	Image cytometry	Unit
*ρ*_spec_	17.8 ± 0.2	-	-	μg L^–1^ count^–1^
*γ* ± *σ*_*γ*_	-	83.9 ± 3.3	0.171 ± 0.003	ag cell^–1^ count^–1^
*I̅*_0_	-	68 ± 22	20,700 ± 5,600	count
*R*^2^	-	0.992	0.999	
*σ*_0_	-	240 ± 46	6,300 ± 770	count
*ξ*	-	1.64 ± 0.09	0.58 ± 0.02	
*σ*_inst_	-	130 ± 38	220 ± 120^b^	count

a Results represent mean ± SD obtained from triplicate samples.

b Measured directly from the InGaAs camera in darkness.

The fitted result gives three parameters, including Hill coefficient *n*, Michaelis constant *k*_M_, and maximum cellular uptake mass *m̅*_max_. The deduced Hill coefficient *n* at ∼1.66, which is much larger than 1, reveals a positively cooperative uptake nature, or an active internalization process, such as receptor-mediated endocytosis of (6,5) into RAW macrophages.^39−42^ The Michaelis constant *k*_M_ at 198.2 μg L^–1^ estimates the required (6,5) concentration to treat the cells for obtaining half of the maximum cellular (6,5) mass. The (6,5) uptake efficiency gradually decreases when treated (6,5) concentration is higher than the Michaelis constant, indicating a saturation of the RAW macrophages’ active transportation. The maximum uptake mass per cell *m̅*_max_ is plateaued at 101.1 fg cell^–1^, which can be considered as the highest possible (6,5) mass when treated with infinitely high (6,5) concentration. Assuming that the average volume of the RAW macrophages is ∼5 pL,^43^ the maximum cellular (6,5) mass concentration *c*_*m*_^max^ is at ∼20.2 mg L^–1^, which is close to the reported range of ∼7–15 mg L^–1^ for HiPco SWCNTs.^44^ The slightly higher *c*_*m*_^max^ in our work might originate from the different incubation condition, surface coating, and types of SWCNTs. In addition, we find that RAW macrophages concentrate (6,5) into the intracellular space by a factor of ∼51 as they are treated with the (6,5) at the *k*_M_ concentration (see full-range estimation in Figure S6c).

### Quantification of (6,5) in SWIR Image Cytometry

Image cytometry allows visualization of cells, aiding the quantification of associated fluorophores. Compared to the flow cytometry, it provides additional information on cell morphology and biomarker distribution. Its required instrument, which is a widefield fluorescence microscope, and related assays are simpler and more attainable than using a flow cytometer. We develop a cytometric SWIR imaging modality based on our custom-built SWIR fluorescence microscope and a machine learning-based data processing.^45^ We exploit similar three-step calibration protocol for (6,5) quantification in cells. Compared to the flow cytometry, the image cytometry requires an extra cell deposition in coverslip wells and a significant postacquisition data processing for accurately counting fluorophore emission intensity from each cell.

Figure 2d shows an example of the acquired brightfield and SWIR fluorescence images of (6,5)-treated RAW macrophages. (6,5) fluorescent light emitted from cell cytoplasm is observed using our deeply cooled InGaAs camera. Red outlined regions delineate cell areas for integrating fluorescence signals, which can be converted into an MFI histogram as shown in Figure 2e and Figure S16a for singlet and whole cell populations, respectively. Similar to the procedure for the flow cytometry, all cells are taken into account for (6,5) quantification calibration, and only singlet cells are selected for the data analysis. Figure 2f shows fitted results of the deduced cellular (6,5) mass as a function of the MFI of the integrated signal within circled cell area, using eq 2 again, and the obtained *γ*, *I̅*_0_, and R-squared are listed in Table 1. The lower *γ* value from the image cytometry comes from the lower gain of the InGaAs camera, while the higher *I̅*_0_ value originates from the summation of cell autofluorescence signals across multiple pixels of the InGaAs camera. The fitted Hill equation in the deduced *C*_*m*_ versus *D* yields parameter values closely resembling those obtained from the SWIR flow cytometer, indicating relevance and reliability of both methods (see Figure 2c and Figure S16c). The ∼20% difference may originate from the lower cell count in the image cytometer, as well as the less consistent focusing on the cells on the coverslip.

### Limit of Detection (LOD) and Signal-to-Noise Ratio (SNR)

LOD provides information about the signal detectability of a specific instrument. In this work, the three-sigma rule, representing the values within three standard deviations (SDs) of the mean (99.7% probability) in a normally distributed population,^46^ was applied to define the value of LOD. That is, the mean (6,5) intensity of the whole cell population *I̅*_(6,5)_^∞^ versus the SD of the mean intensity of the unstained cells *σ*_0_ should be greater than three, or
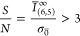
4

**Figure 3:**
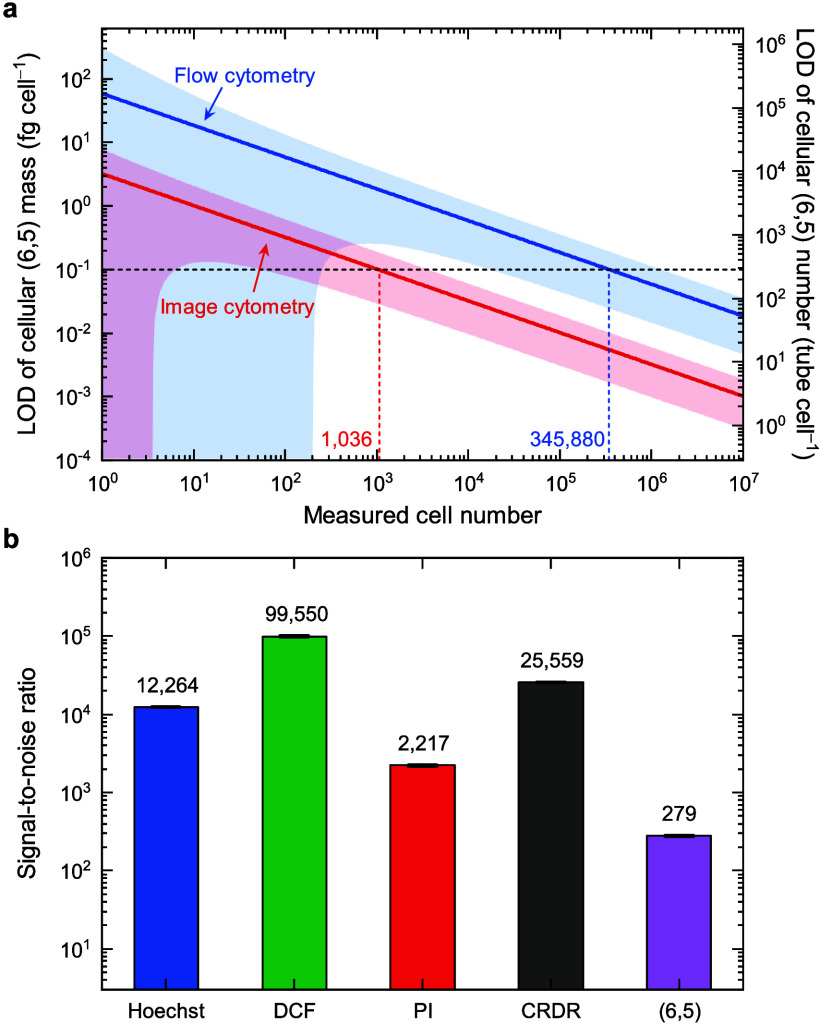
Limit of detection (LOD) and signal-to-noise ratio (SNR). (a) Estimated LOD of cellular (6,5) mass (left) and cellular (6,5) number (right) using SWIR flow and image cytometers. The red and blue areas represent SDs of the two simulated lines. (b) The SNR of Hoechst, DCF, PI, CRDR, and (6,5) in the flow cytometry. Results are mean ± SD obtained from triplicate samples.

The LOD, or minimum detectable level, of the cellular (6,5) mass *m̅*^LOD^ and its deduction error *σ*_*m̅*^LOD^_(*N*) can then be estimated from the following equation
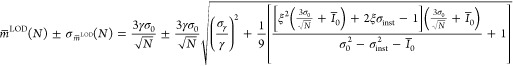
5where *N* is the number of measured cells, *σ*_*γ*_ is the SD of *γ*, *ξ* is the slope of the SD with respect to the MFI of the measured signal, and *σ*_inst_ is the instrument noise. The detailed derivation from eq 4 to eq 5 is provided in the Discussion S15. The *m̅*^LOD^ is inversely proportional to the square root of the measured number of cells *N*, as shown in Figure 3a for both flow and image cytometers, with the parameters of the equation listed in Table 1. We note that the two simulated lines for flow and image cytometries in the log–log plot are in parallel. Thus, the flow cytometry requires ∼334 times the measured cell numbers than the image cytometry to achieve similar LOD, or the LOD of the flow cytometry is ∼18 times higher than that of the image cytometry with the same measured cell counts (see Discussion S16). The much-lowered sensitivity using flow cytometry stems from its shorter acquisition time (∼10 μs per cell vs 7 s per frame), less efficient spectral filtration, lower quantum efficiency of the detector (∼55% for Si APD vs ∼65% for InGaAs camera), and higher detector temperature (295 K vs 83 K), resulting in larger thermal noise. Nevertheless, the experimental rate of the flow cytometry is ∼608 times faster than that of the image cytometer (see Table S6). Therefore, the required experimental time using the flow cytometer is ∼1.82 times shorter than that using the image cytometer. In other words, the high throughput feature of the flow cytometry significantly compensates its low sensitivity drawback. To reach an LOD of the cellular (6,5) mass *m̅*^LOD^ at the 0.1 fg cell^–1^ level, 345,880 and 1,036 measured cells are required for the flow and image cytometry, respectively. This aligns with the practical numbers of cell measurements, falling within 10^5^–10^6^ for flow cytometry and 10^3^–10^4^ for image cytometry. The LOD at 0.1 fg cell^–1^, or ∼284 tubes cell^–1^, is ∼1011 times less than the maximum (6,5) uptake, as estimated in Figure 2c using Hill equation, indicating both systems give reasonable sensitivities for studying (6,5) signals in cells.

Although both methods reach similar performance in our systems, we note that the instrument design and sample conditions can significantly influence and result in divergent outcomes. The SNR of a cytometer detection channel is determined by the detector quantum efficiency, detector noise, and fluorophore brightness. In Figure 3b, we compare the SNR of (6,5) with four commonly used visible fluorophores in the flow cytometer: Hoechst 33342 (used for staining nucleus DNA for cell cycle analysis), DCFH-DA (2′,7′-dichlorofluorescein diacetate, for reactive oxygen species (ROS) detection, turning into DCF fluorescent form), PI (propidium iodide, for staining dead cells), and CellROX deep red (CRDR, another probe for ROS detection). All channels achieve SNRs larger than 100. The relatively lower SNR of the (6,5) channel may be attributed to its lower detector quantum efficiency and fluorophore quantum yield. However, the low autofluorescence, which gives ∼60 counts in the SWIR compared to ∼3k counts in the NIR and ∼10k in the visible, helps maintain in a reasonable SNR, even with a lower signal count (see Figure S11). The SNR of (6,5) in the image cytometer is also presented in Figure S16d.

### Fluorescence Spillover and Its Compensation

**Figure 4:**
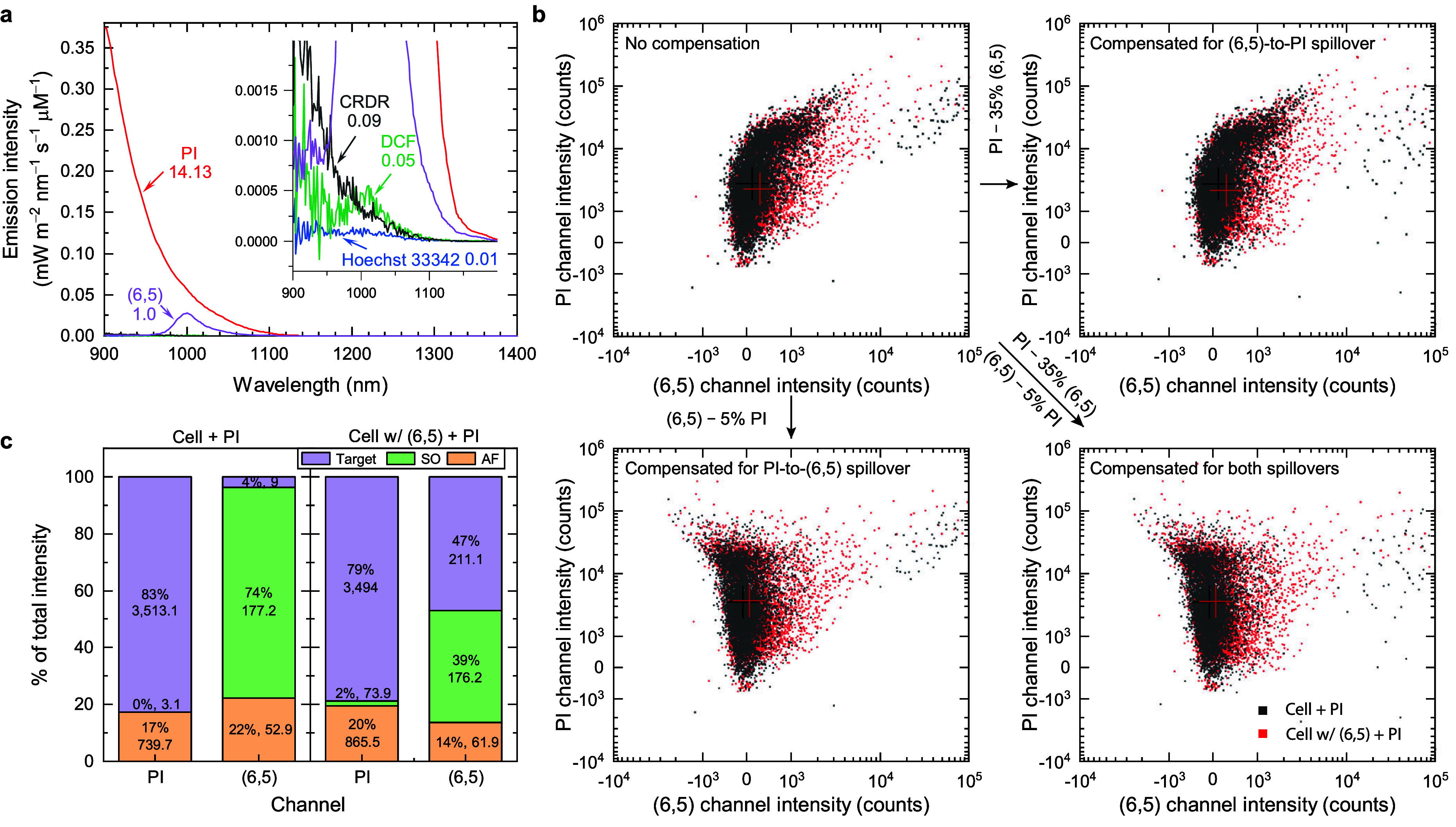
Fluorescence spillover between (6,5) and several other commonly used visible fluorophores for flow cytometric measurements. (a) SWIR fluorescence spectra of (6,5), PI, CRDR, DCF, and Hoechst 33342. The emission intensity is normalized to molar concentration of the fluorophores and multiplied by the detector spectral efficiency. A 50-carbon (6,5) is assumed to reach similar molecular weight compared to other fluorophores. Inset zooms in the figure for visualizing the spectra of CRDR, DCF and Hoechst 33342. The relative integrated molar emission intensity normalized to that of (6,5) in the range of 900–1100 nm are labeled below the fluorophore names. (b) Spillover compensations between PI and (6,5) channels. The crosses in the figure represent the median intensities of PI and (6,5) channel intensities. Approximately 170,000 cells are presented. (c) Deduced intensity distributions among target fluorescent signals (purple), spillover signals (green), and autofluorescence background (orange). The cellular (6,5) mass is ∼17.7 fg cell^–1^ in this case.

The fluorescence spillover is the inevitable collection of the fluorescent signal from one fluorophore by a detection channel intended for a different fluorophore. The spillover effect leads to an overestimation of fluorophore (target) signals when the emission spectra of two or more fluorophores overlap. This is a common issue in the visible and near-infrared range when many fluorophores are stained, and their fluorescence needs to be acquired simultaneously. SWIR window is known to pertain lower autofluorescence,^47^ but the emission tails of visible fluorophores that spill into the SWIR window are rarely studied.^48^ Here, we examine the extent of spillover of the four above-mentioned fluorophores, including Hoechst 33342, DCFH-DA, PI, and CRDR, into the (6,5) channel. Figure 4a shows SWIR fluorescence spectra of these fluorophores using a 561 nm laser excitation. The emission intensities are normalized to the molar concentration of the fluorophores, allowing a comparison of the relative emission brightness among fluorophores. We observe that the PI emission tail is ∼14 times stronger than the (6,5) emission, potentially resulting in significant spillover. In contrast, the emission intensities of the other three fluorophores are at least 10 times smaller than that of (6,5).

The spillover signals can be compensated using the following generalized equation
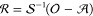
6where  is the real signal vector,  is the spillover matrix,  is the observed signal vector, and  is the autofluorescence vector (see Discussion S19). Figure 4b shows intensity scatter plots of PI and (6,5) channels with and without spillover compensations. The uncompensated data reveals that the spillover signals contribute to the higher emission intensities from some cells, leading to extra population in the upper-right region along the diagonal line. The compensation of the (6,5) channel for the PI spillover signals (lower left panel) leads to significant shifts in cell population from the upper right toward the upper left, resulting in a more symmetric distribution in the axis of (6,5) channel intensity. In addition, the compensation of the PI channel for the (6,5) spillover (upper right panel) shifts some of the upper right cell population toward the lower right. In theory, the (6,5) should not emit any fluorescence at the 690 nm. We suspect that the spillover comes from the imperfect spectral filtration of the (6,5) and/or the residual (9,1) emissions into the PI channel. Moreover, the (6,5)-to-PI spillover coefficient calculated based on eq 6 overcompensates the PI channel intensities, giving an extra tail below the main population, as shown in Figure S21. The overcompensation may originate from the ∼17% higher autofluorescence level of the (6,5)-treated cells than the non-(6,5)-treated ones (Figure S22a), as evidenced by the observed larger cell sizes in the (6,5)-treated groups (Figure S22b) that lead to higher autofluorescence.^42,49^ This issue has been corrected by reducing the spillover coefficient and then observing the shape of the intensity distribution, as shown in Figure S23. In contrast, the deduced PI-to-(6,5) spillover coefficient results in only ∼5.7% overcompensation, probably due to the lower autofluorescence level in the SWIR window.

The extent of the spillover in each sample and each channel can be visualized in Figure 4c. In the group of cells without (6,5) treatment, the PI fluorescence contributes to 83% of the total intensity observed in the PI channel, while the PI fluorescence spillover contributes to 74% of the observed (6,5) channel intensity. The absence of (6,5) results in nearly no spillover in the PI channel, while the 4% target signal in the (6,5) channel is attributed to measurement uncertainties. In the group of (6,5)-treated cells, only ∼2% (also within measurement uncertainty) of the total PI intensity comes from the spillover of the SWCNT fluorescence, while ∼39% of the (6,5) channel intensity is attributed to the spillover of the PI fluorescence. Therefore, in the case of PI and (6,5) costaining, the spillover compensation in the (6,5) channel is essential because the spillover intensity is over 80% of the (6,5) fluorescence intensity.

### Correlation of Intracellular (6,5) and ROS Levels in RAW Macrophages

Understanding the impact of the SWIR fluorescent nanomedicine on cellular responses, particularly in relation to oxidative stress, is of longstanding interest.^50,51^ The simultaneous analysis of a SWIR fluorophore and visible probes for the desired biomarkers requires a modality embedded with a detector that works in both visible and SWIR spectral windows. Here, we utilize our Visible-NIR-SWIR flow cytometer to study the statistical correlation of intracellular (6,5) mass and ROS production from single-cell measurements. Some previous studies also aimed at observing similar objects,^52−55^ but the extraction of their correlations was challenging. In addition, the SWCNT sample quality, especially the aggregation state, species purity, and surface coating,^56^ were different and sometimes not well characterized, leading to ambiguous or conflicting results among them. We ensure the individualization and purity of (6,5) as mentioned in the first section. RAW macrophages are first treated with (6,5) and then stained with CRDR to evaluate the intracellular ROS levels.

**Figure 5:**
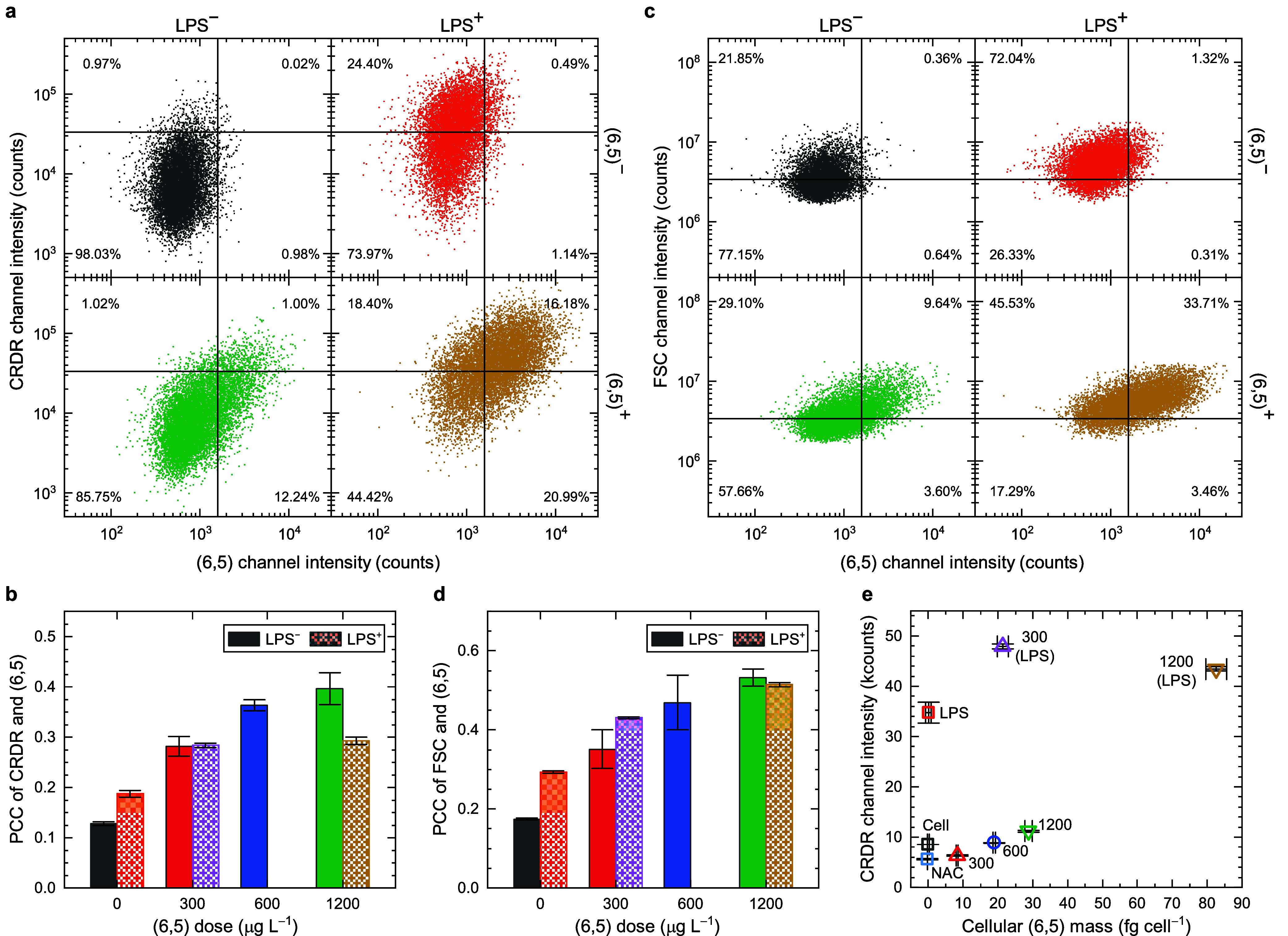
Investigation of the correlation between intracellular (6,5) accumulation and ROS generation. (a) The scatter plots of fluorescence intensities from CellROX deep red (CRDR) and (6,5) channels from singlet RAW macrophage with and without LPS and (6,5) treatments (singlet-cell gated). (b) The Pearson correlation coefficients (PCCs) of CRDR and (6,5) at different (6,5) doses. Both LPS positive (LPS^+^) and LPS negative (LPS^–^) are presented. (c) The scatter plots of FSC intensity vs (6,5) channel intensity with and without LPS and (6,5) treatments from (singlet-cell gated). (d) The PCCs of FCS and (6,5) at different (6,5) doses. Both LPS^+^ and LPS^–^ are presented. (e) The mean fluorescence intensities of CRDR and cellular (6,5) mass of samples with different (6,5) doses (unit: μg L^–1^) and LPS treatments. Cell and NAC represent nontreated and NAC-treated RAW macrophages, respectively. Approximately 30,000 cells are presented in all cases. The error bars represent SD from triplicate samples.

Lipopolysaccharide (LPS) treatment is a common technique to induce higher ROS level in the cells. The non (6,5)- and non LPS-treated groups show a near-symmetrical shape that represents autofluorescence and system noise distributions (see Figure 5a). The addition of (6,5) largely shifts the population toward higher (6,5) channel intensity with a small intensity increase in the CRDR channel. The treatment of LPS significantly increases the CRDR_ch_^+^ from 0.99% to 24.89%. The CRDR_ch_^+^ population gives a slight increase of (6,5) channel signals, indicating an increase of autofluorescence level after LPS treatment. The dual-treated group further increases the (6,5)_ch_^+^ from 1.63% to 37.17%, while the increase of the CRDR_ch_^+^ is limited to only 9.69%. The correlation between CRDR and (6,5) signals can be analyzed using Pearson correlation coefficients (PCC), as shown in Figure 5b. The PCC values greatly rise with increasing (6,5) doses, indicating that the accumulated (6,5) could stimulate ROS production. This relation seems to align with previous reports that the carbon nanotubes could induce the NOX-mediated respiratory burst after endocytosis.^41,50^ This phenomenon is less evident in the LPS^+^ group, probably due to the leading factor for ROS production coming from the LPS treatment, not (6,5).

The size and morphology of the cells change with different treatments, as observed in the previous section, and can be evaluated using the scattering signal. The FSC signal is known to reveal the relative sizes of the cells.^57^ The addition of LPS moves ∼51% of the cell population from FSC^–^ to FSC^+^ region, revealing the increased cell size after treatment (see Figure 5c). Meanwhile, an increase of the (6,5)_ch_^+^ for ∼0.6% is also observed, indicating the increased autofluorescence in the SWIR. The simultaneous increase of these two parameters suggests a positive correlation between the size and autofluorescence of the cells, leading to a higher PCC at zero (6,5) dose after LPS treatment (shown in Figure 5d). The (6,5) treatment alone seems to show smaller size increase, but the PCC between FSC and (6,5) reaches a similar level for LPS^–^ and LPS^+^ groups at the highest (6,5) dose. Similarly, we also find strong correlation between side-scatter signal (SSC) and cell treatments, indicating that the treatments increase the cell granularity/complexity (see Figure S25).

Figure 5e summarizes the results of the median CRDR channel intensity with respect to the cellular (6,5) mass. Notably, we observe a lower intracellular ROS level at a low cellular (6,5) mass, ∼8.3 fg cell^–1^, than no (6,5). It has been reported that highly covalently functionalized 60 nm SWCNTs exhibit some antioxidant feature in simple chemical environment,^58^ but this phenomenon has not been observed in the cells, to the best of our knowledge. The ROS level gradually increases with the rise of the cellular (6,5) mass, likely due to increased (6,5)-induced oxidative stress, albeit milder than that observed in the LPS-treated group. Interestingly, while LPS activates the RAW macrophages to produce more ROS, LPS-activated RAW macrophages appear to enhance the endocytosis further,^59,60^ engulfing even more (6,5) under the same (6,5) dose concentration.^61^ The two types of (6,5) uptake—enhanced by (6,5) itself versus by LPS—might involve different stimulation mechanisms, as indicated by the distinct (6,5) dose-dependent increases in intracellular (6,5) mass (shown in Figure S24f). However, further studies are needed to understand their detailed mechanisms.

## Conclusions

In this work, we demonstrated the effectiveness and efficiency of two SWIR-based cytometric techniques, SWIR flow cytometry and SWIR image cytometry, using (6,5)-SWCNTs as a model fluorophore. These methods serve as statistically relevant quantitative tools for analyzing SWIR fluorescent signals emitted from individual or clustered cells. The limit of detection (LOD) for (6,5) reaches a level of ∼0.1 fg cell^–1^ within a half-hour experimental time frame, facilitated by high-throughput counting for flow cytometry and high sensitivity detection for image cytometry. It is reported that a minimal dose of ∼100–200 ng SWCNTs is sufficient for in vivo imaging and diagnostic applications.^7,17^ The fate of these nanoparticles is very likely to be in the liver macrophage Kupffer cells.^62^ The number of Kupffer cells in an adult mouse liver is ∼141.75 million (Adult mice liver usually weighs 2–3 g with a cell density of ∼135 M cell g^–1^,^63,64^ including ∼35% Kupffer cells^65,66^). Assuming all the administered (6,5) eventually accumulate evenly in the Kupffer cells, the cellular (6,5) mass will be ∼0.7 fg cell^–1^, which is at least seven times higher than the LOD of our systems. Therefore, it is feasible to study the cellular responses and toxicity of (6,5) down to the minimal level that is relevant for in vivo diagnosis and imaging purposes. Besides, our in vitro studies show that the RAW macrophages concentrate the surrounding (6,5) by a factor of ∼51, and their maximum uptake concentration is predicted to reach ∼101.1 fg cell^–1^ in some extreme cases. When cells are stained with a second fluorophore that has strong emission tail in the SWIR regime, spillover compensation needs to be considered and applied. In the case study of the correlation between ROS level and accumulated (6,5) mass, we obtain higher Pearson correlation coefficients when cells are treated at higher (6,5) concentrations, so as the correlation of cell size and granularity with (6,5) channel signal. These values are valuable as they are unlikely to be extracted from ensemble measurements. Furthermore, high sensitivity measurements enable observation of antioxidation effect of (6,5) at a low dose level. We also find that higher cell stress results in greater (6,5) uptake, and the uptake of (6,5) is positively cooperative, as indicated by the deduced Hill coefficient, which is much larger than one.

Our developed SWIR cytometers, with appropriate spectral filters, can be adapted for use with any SWIR emissive materials, as demonstrated with (6,5)-SWCNTs. The expansion of an extra 200 nm detection window up to 1,100 nm in the flow cytometer provides a possible 40% addition to the existing visible/NIR channels. Our SWIR image cytometer can further extend the detection wavelength up to 1550 nm, providing enormous possibilities for biomarker identifications. For example, SWCNTs alone have more than 35 different species that emit at wavelengths in the range of 900–1550 nm, and all of them can be well distinguished using seven different colors of excitations.^67,68^ An interesting use is to stain and analyze more than 40 biomarkers simultaneously, combined with the conventional visible/NIR cytometer that already allows up to 40 detection channels.^28^ Further development of an appropriate detector with an extended spectral window up to 1550 nm for the flow cytometer is needed in the future to achieve the same capability as the SWIR image cytometer. To some extent, we may still prefer to conduct research using flow cytometry rather than image cytometry, as extra sample preparation procedures and significant postacquisition data processing are required for the later method. Our initial demonstration has already proven the feasibility of the SWIR cytometry, and future refinements and improvements in instrumental design and fluorophores, in conjunction with the current visible/NIR modality, will establish it as an indispensable tool for cell studies.

## MATERIALS and METHODS

### (GT)_20_-Coated (6,5) Preparation

Our single-chirality (6,5) samples were purified from individualized SG65i CoMoCAT SWCNTs (Lot #: SG65i-L67, Chasm) using the aqueous two-phase extraction (ATPE) method. Briefly, CoMoCAT powder was added to a 1% (w/v) sodium deoxycholate (SDC) (30970, Sigma-Aldrich) solution at a concentration of 1 g L^–1^. Tip sonication (Q700, Qsonica), with an amplitude of 35, was applied for 72 h (30 s on and 30 s off). Subsequently, individual (6,5) CoMoCATs were separated by collecting the supernatant postultracentrifugation (150,000 *g*, 90 min). The ATPE method, detailed in the Method S1, was utilized for the isolation of single-chirality (6,5).

Single-stranded DNA (GT)_20_ (Integrated DNA Technologies) was exploited to coat the (6,5) surface using a modified protocol from a prior study.^69^ Specifically, (GT)_20_ was mixed with a 100 μL (6,5) solution (1% sodium cholate), giving a final (GT)_20_ concentration of 1.65 g L^–1^. Methanol (7.5 μL) was added dropwise into the solution under vortex. The resultant mixture was then carefully dropped into 500 μL of isopropanol to induce precipitation of both (GT)_20_ and (GT)_20_-coated (6,5), followed by a 5 min centrifugation (3000 *g*) to collect the pellet. The pellet was transferred to a 1.5 mL centrifuge tube and rinsed thoroughly with 5 μL phosphate buffer (0.05 M) several times to ensure complete removal of residual isopropanol. Subsequently, 200 μL of phosphate buffer was added to the tube, and a 30-s tip sonication (amplitude 1, 5 s on, 10 s off) was applied to resuspend the (GT)_20_-coated (6,5). Finally, the sample underwent five rounds of 15 min centrifugation at 21,100 *g* at 4 °C to eliminate SWCNT aggregates. The resulting sample was stored in a 4 °C refrigerator until use.

For the agglomeration tests specifically, both supernatant and pellet were collected separately after five rounds of 15 min centrifugation as described above, representing individualized and agglomerated samples, respectively. These SWCNT samples were then dropcast onto coverslips at an appropriate concentration (∼0.1 OD at S_11_), followed by taping the coverslips on a slide for SWIR imaging using our custom-built SWIR microscope system. The detailed acquisition parameters were the same as those described in Method S6 for measurements conducted with HiPco SWCNTs.

### Skewness Analysis by Variance Spectrometry

Variance spectroscopy was performed based on the gravity model previously reported by Weisman’s group.^70^ The optical setup resembled that described in the referenced paper, with a 561 nm excitation laser and a 200-μm-path-length cuvette (48-Q-0.2, Starna Cells), replacing the 785 nm laser and the 100-μm-path-length cuvette, respectively. The height between the collection (lower) and source (upper) 1.5 mL centrifuge tubes was adjusted to achieve an optimal flow rate of ∼50 μL min^–1^. A total of 2000 spectra were captured over a fixed period of 300 ms, with an exposure time of 50 ms, resulting in a total acquisition time of ∼8.35 min.

In the data processing, the extreme outliers that exceeded five time the standard deviation were excluded. Subsequently, laser fluctuation was estimated using a highly smoothed, average signal curve in the frame series (101 smoothing window), and corrected by subtracting the raw data from this estimated average signal curve. Mean, variance, covariance, and skewness spectra were then calculated.

A multispecies standard sample was prepared for system validation purpose. We prepared a SWCNT sample with multiple species in 1% SDC using HiPco powders (Batch#: HR35-188, NanoIntegris). The well-suspended and highly individualized SWCNTs were obtained by collecting the upper part of the sample supernatant right after ultracentrifugation (280,000 *g* for 1.5 h). The agglomerated sample was collected.

### In Vitro Sample Preparation

Murine macrophage RAW264.7 (60001, Bioresource Collection and Research Center, Taiwan) served as our model cell line. Parental cells were seeded into a 6-well plate at a concentration of 500,000 cells well^–1^ and cultured for 1 day at 37 °C with 5% CO_2_. Following the removal of medium and the washing of the cells with Dulbecco's phosphate-buffered saline (DPBS), (GT)_20_-coated (6,5) samples in DPBS solutions were added at various concentrations. A 30 min coincubation period was given under cell culturing conditions, followed by three DPBS washes and cell detachment using Accutase. The cell pellet was collected by centrifugation (100 *g*, 10 min) and resuspended in 100 μL DPBS.

For flow cytometry analysis and SWIR microscope imaging, 20 μL of the cell suspension was taken and diluted with 1 mL of DPBS. An additional 5 μL of the remaining suspension was used for cell counting (Invitrogen countess 2, Thermo Fisher Scientific), and the volume of the remaining part was measured to determine the total number of cells in the sample. This remaining cell suspension was utilized for fluorescence and absorbance measurements. A detailed record of the volumes is available in Table S3. A viability experiment was conducted, revealing no observable cytotoxicity of (GT)_20_-coated (6,5) SWCNTs, as depicted in Figure S27.

For fluorescence and absorbance measurements, the cell suspension underwent initial tip sonication for 5 s at an amplitude of 1. Subsequently, 20 μL of a SDC solution (10% w/v) was added to the suspension, and the solution was then diluted with deionized (DI) water to a final volume of 200 μL. An ice-bath was employed during sonication. Another round of tip sonication (5 s on; 10 s off; 1 min total active time) was used to ensure proper resuspension of SWCNTs within the sample. Finally, 190 μL of the sample was collected for measurement.

For microscope imaging, 200 μL of cell suspension from the flow cytometry sample was taken for cell fixation using a 4% formaldehyde solution in DPBS for 10 min. Subsequently, the fixed cells were washed with DPBS via centrifugation (300 *g*, 5 min), and then placed in an 8-well chamber coverglass (Lab-Tek Chambered Coverglass, 155411, Nunc) for microscope imaging. After adding the cells to the wells, the slide was positioned on a flat shaker for 10 min, followed by a resting period of ∼30 min to allow the cells to attach to the bottom. The estimated cell number in each well was ∼30,000, as observed and depicted in Figure S28.

### Ensemble Spectral Measurements

The absorption spectra in the range of 200–1100 nm were measured using a commercial UV–visible spectrophotometer (V-750, Jasco), while that in the range of 400–1400 nm were measured using a custom-built Visible-SWIR spectrophotometer with dual detectors (USB-2000, Ocean Optics & Kymera 328i + iDus 1.7, Andor). The SWIR fluorescence spectra were measured using the same custom-built system equipped with a CW laser (Sapphire LPX 561, Coherent). The laser was tightly focused using a UV fused silica aspheric lens (#33-949, Edmund Optics). The emission was collected using the same lens and then refocused into a 50 μm core-sized multimode optical fiber, followed by the transmission into a spectrograph. In the concentration calibration procedure, the detector background has been subtracted during the measurement to give zero intercept.

### SWIR Flow Cytometric Instrumentation and Measurements

A commercial flow cytometer equipped with APDs (CytoFLEX, Beckman) was modified with a custom filter set, incorporating a 900 nm long-pass filter installed in the first channel of the 561 nm excitation module. This specific detection channel is referred to as the (6,5) channel, featuring 561 nm excitation and 900 nm long-pass detection. During the measurements, the voltage gains of the detectors for the FSC, SSC and (6,5) channels were set to 430, 150 and 3000, respectively. The flow rate for the measurements was established at a medium rate of 30 μL min^–1^, with ∼1000 cells counted per second, adjusted through suitable sample dilution. The counted cell number exceeded 20,000 if not specified. Besides, the total measurement time for all samples was controlled to be under three hours to prevent cell death. In the ROS vs (6,5) case study, CRDR was excited at 633 nm and its emission was transmitted through a 690 nm bandpass filter with 50 nm bandwidth, allowing the evaluations of the amounts of ROS generation.

### SWIR Image Cytometric Instrumentation and Measurements

A home-built SWIR microscope, equipped with an inverted microscope stage (ECLIPSE Ti2-E, Nikon), an InGaAs camera (NIRvana LN, Princeton Instruments), an LED source (CHROLIS-C1, Thorlabs) and a 900 nm long-pass filter set (FELH0950, DMLP950R, and FESH0950, Thorlabs), was utilized. The power of the 565 nm LED at the objective exit was measured at 19.70 ± 0.05 mW (see Figure S29). A 40× objective (CFI Plan Apo λD 40×/0.95, Nikon) was employed for acquiring all images for analysis. A custom LabVIEW program was developed to autonomously control the cameras, motorized microscope XY stage, filter sets, and LED lights, enabling acquisitions of bight-field and SWIR fluorescence images interchangeably at different locations. PFS function was applied to maintain the Z focus unchanged between image locations. The nondestructive readout mode (0.5 s readout, total 7 s) of the InGaAs camera was utilized to obtain images with minimal background signal. A total of 70–150 images were captured in each group, depending on the cell density in the well.

### Experimental Procedure for the Spillover Compensation

Hoechst 33342 (14533, Sigma-Aldrich), DCFH-DA (D399, Invitrogen), propidium iodide (PI) (P4170, Sigma-Aldrich) and CellROX deep red (CRDR) (C10422, Invitrogen) were utilized to investigate their signal spillovers into the (6,5) channel. RAW macrophages were initially seeded in a 6-well plate with 700,000 cells per well for 1 d.

For DCFH-DA and CRDR, lipopolysaccharide (LPS) was used as a positive control. Cells were treated with LPS (1 g L^–1^) for 8 h, followed by washing with DPBS. (6,5) was added to the well for a 30 min coincubation at a concentration of 600 *μ*g L^–1^. After DPBS washing, a 30 min DCFH-DA or CRDR staining was conducted at concentrations of 10 or 5 μM, respectively. After DPBS washing and collecting the cells, the cell suspension samples were kept on ice before flow cytometry measurement. Besides (6,5)^+^/DCF^+^, the (6,5)^−^/DCF^+^ and (6,5)^+^/DCF^–^ groups and nonstained groups were also prepared.

For PI staining, cells treated with doxorubicin (DOX) at a concentration of 0.5 mg L^–1^ were used as the PI^+^ control. Cells were coincubated with DOX for 12 h, followed by DPBS washing and a 30 min (6,5) treatment. PI staining (1 mg L^–1^) was conducted for 10 min. After washing and cell collection, the cell suspensions were kept on ice before measurement. For Hoechst 33342 staining, the process was applied at a concentration of 10 mg L^–1^ for 10 min after a 30 min (6,5) treatment.

The parameters for flow cytometry measurement were similar to previous experiments, except for adding an additional excitation and detection channel for each dye. For Hoechst 33342, an excitation of 405 nm and an emission of 450/45 nm were used. For DCF, an excitation of 488 nm and an emission of 525/40 nm were used. For PI, an excitation of 488 nm and an emission of 690/50 nm were used. For CRDR, an excitation of 638 nm and an emission of 660/10 nm were used. The spillover and compensation calculations are described in Discussion S19–22. Additionally, the SNRs for this experiment were calculated by dividing the MFI of the fluorophore in the single-stained group by the standard error of the mean (SEM) of the autofluorescence in the nonstained control group.

### Correlations between (6,5) Mass and ROS Level Using SWIR Flow Cytometry

Intracellular ROS levels were measured using the fluorescent probe CellROX deep red (C10422, Invitrogen). Briefly, RAW 264.7 macrophages were seeded into a 6-well plate (500,000 cells per well) and incubated at 37 °C with 5% CO_2_ for 1 day. For the LPS pretreatment group, LPS was added to the medium at a final concentration of 1 g L^–1^, 6 h before (6,5) treatment. Cells were washed with DPBS, and 1 mL of (6,5) solutions with various concentrations (300, 600, and 1200 μg L^–1^) in DPBS solution were added for 30 min of coincubation. After washing the cells with DPBS, fresh medium was added to the well for an additional 12 h of incubation, followed by washing with DPBS, adding the CRDR probe with a final concentration of 5 μM, and then incubating for 30 min. Cells were collected using a cell scraper and then centrifuged. After resuspending the cell pellets in DPBS, flow cytometry measurements were conducted. The excitation and emission wavelengths of the CRDR channel were 638 nm and 690/10 nm, respectively. It is important to note that an additional experiment for setting up the calibration line should be conducted simultaneously, as described in the previous experimental section.

The fluorescence signal data were acquired using Beckman CytoFLEX and processed with FCS Express to convert the raw data into txt format. Cell populations were gated as illustrated in Figure 1. The live cell population was gated in the FSC-vs-SSC plot, followed by a singlet-cell population gating in the FSC-H-vs-FSC-A plot. MATLAB was utilized to extract the desired signal counts from the raw data and set the bins for the histogram plot. PCC was calculated as a simple method to examine the correlation between ROS levels and cellular (6,5) amounts.

### Data Analysis and Statistics

The flow cytometry results were exported in FCS file format using CytExpert software, followed by gating the cells and exporting the raw/processed data using FCS Express. For image cytometry, image analysis, including cell segmentation, total signal counting, and plotting, was conducted using Python with the Cellpose 2.0 package.^45^ The regions of interest (ROIs) for cells in all image sets, including brightfield and SWIR images, were chosen based on the cell segmentation function with a predefined cell diameter of 30 μm in Cellpose 2.0. Following ROI selection, an additional script was developed to identify cell aggregates and sum the counts in each ROI, allowing for the determination of total emission in singlet cells. Further details are provided in Discussion S13. The code is publicly available on GitHub (https://github.com/jsw99/photonic-nanomaterials-lab/tree/main/Cell-Segmentation). All measurement data, whether from flow or image cytometers, were processed in MATLAB for intensity and LOD analyses. OriginPro was employed for figure plotting, curve fitting, and PCC calculations.

## Supporting Information Available

Additional experimental methods for aqueous two-phase extraction for single chirality (6,5) separation, investigation of the environmental effect on (6,5) emission, resuspension of the intracellular (6,5) for spectral measurements, visualization of (6,5) uptake, effect of formalin fixation, aggregation-state examination and cytotoxicity. Additional discussion of aggregation state determination, cellular uptake, viability, fixation effect, Hill equation fitting, relation of autofluorescence and (6,5) intensity spreading, background level, factors for effecting autofluorescence, concentration deduction, LOD equation derivation, cell segmentation process, spillover compensation, and the relation of cellular (6,5) mass and cellular response (PDF)

## ABBREVIATIONS

SWIR: Short-wave infrared
NIR: near-infrared
SWCNT: single-wall carbon nanotube
LOD: limit of detection
SNR: signal-to-noise ratio
APD: avalanche photodiode
InGaAs: Indium Gallium Arsenide
LED: light emitting diode
SSC: side scatter
FSC: forward scatter
MFI: mean fluorescence intensity
fwhm: full width at half-maximum
HiPco: high-pressure carbon monooxide
SD: standard deviation
ROS: reactive oxygen species
LPS: lipopolysaccharide
ROI: region of interest
PCC: Pearson correlation coefficient
DPBS: Dulbecco's phosphate-buffered saline
