# Supplementary material for: Cytometry in the Short-Wave Infrared
Source: ACS Nano. 2024 Jul 8;18(28):18534–47. doi: 10.1021/acsnano.4c04345 (PMC11256901; doi:10.1021/acsnano.4c04345)
Supplement: Supplementary file 1 — nn4c04345_si_001.pdf [file nn4c04345_si_001.pdf]

Supporting Information

Cytometry in the Short-Wave Infrared

Te-I Liu[1,], Jhih-Shan Wang[1,2,3], Ai-Phuong Nguyen[1,4], Marco Raabe[1], Carlos Jose Quiroz Reyes[1,5], Chih-Hsin Lin[6], Ching-Wei Lin[1,]**

[1] Institute of Atomic and Molecular Sciences, Academia Sinica, Taipei City 106319, Taiwan

[2] Department of Materials Science and Engineering, National Taiwan University, Taipei City 106319, Taiwan

[3] Department of Physics, University of Stuttgart, Stuttgart 70174, Germany

[4] Department of Chemistry, National Tsing Hua University, Hsinchu 300044, Taiwan

[5] International Ph.D. Program in Biomedical Engineering, Taipei Medical University, New Taipei City 235603, Taiwan

[6] Graduate Institute of Nanomedicine and Medical Engineering, Taipei Medical University, New Taipei City 235603, Taiwan

Contents

Methods 3
Method S1. Aqueous two-phase extraction (ATPE) for single chirality (6,5) separation 3
Method S2. Investigation of the environmental effect on (6,5) emission 3
Method S3. Resuspension of the intracellular (6,5) for spectral measurements 4
Method S4. Visualization of (6,5) uptake in RAW 264.7 macrophages 4
Method S5. Effect of formalin fixation on autofluorescence signals detected by flow cytometry 4
Method S6. Preparation of HiPco SWCNTs for aggregation-state examination 4
Method S7. Evaluation of the cytotoxicity of $(GT)_{20}$-coated (6,5) 5
Discussion 6
Discussion S1. Determination of aggregation states of HiPco SWCNTs using SWIR microscope 6
Discussion S2. Determination of aggregation states of (6,5) using skewness analysis 7
Discussion S3. Time-dependent (6,5) uptake of RAW macrophages 10
Discussion S4. Fixation effect on the fluorescence detection of flow cytometry 11
Discussion S5. Gating strategy and the comparison of (6,5) levels in singlet and whole populations 12
Discussion S6. (6,5) dose-dependent MFI fitted with Hill equation 14
Discussion S7. Relation of autofluorescence and (6,5) intensity spreading 17
Discussion S8. Effect of the residual cellular components on (6,5) spectroscopic measurements 18
Discussion S9. Background level of cellular autofluorescence 19
Discussion S10. Deduction of (6,5) concentration using its fluorescence 20
Discussion S11. Factors that influence the autofluorescence level in flow cytometry 23
Discussion S12. Cell cluster effect to the cellular fluorophore mass function 24
Discussion S13. Cell segmentation with ROI analysis in image cytometry 26
Discussion S15. Calculation of the limits of detection of the SWIR cytometers 31
Discussion S16. Comparison of the LODs of the two cytometric methods 37
Discussion S18. Fluorescence spectra of fluorophores used in this work 40
Discussion S19. Spillover compensation 41
Discussion S20. Calculation of spillovers between dyes and (6,5) 43
Discussion S21. Spillover compensations between dyes and (6,5) channels 44
Discussion S22. Investigation of the overcompensation in the case of PI-(6,5) system 45
Discussion S23. The relation of cellular (6,5) mass and cellular response using SWIR flow cytometry 49
Discussion S24. The correlation of cell size and cell autofluorescence 53
Discussion S25. Evaluation of the cytotoxicity of $(GT)_{20}$-coated (6,5) SWCNTs 54
References 56

Methods

Method S1. *<u>Aqueous two-phase extraction (ATPE) for single chirality (6,5) separation</u>*

We purified CoMoCAT SWCNTs using a modified ATPE method from a previous report.[1] The separation involves three steps: concentration, large diameter removal, and small diameter removal. In brief, a mixture of 20 wt% Dextran T70 (DX) solution, 40 wt% PEG 6000 (PEG) solution, and 1% CoMoCAT solution in a 1:1:2 ratio was prepared in a 250 mL bottle. A centrifugation (3000 ×g, room temperature) for 5 min was then applied to facilitate the phase separation, followed by a collection of the black bottom phase containing SWCNTs using a syringe with a needle or thin tube and then transferred into a new bottle. Volume of solution was recorded.

For the second step, the bottom-phase solution from the first step was mixed with a sample with 20 wt% DX solution, 40 wt% PEG solution, and 10% w/v SDS solution, and water in a volume ratio of 0.1:0.35:0.175:0.05:0.325. The solution's pH was adjusted with HCl to move SWCNT species with a diameter larger than (6,5) to the top phase, followed by centrifugation for phase separation. The bottom pinkish-purple phase was collected for the next step separation.

In the final step, the bottom phase obtained from the previous step was mixed with a top mimic phase (15 wt% PEG solution, 0.5% w/v SDS solution, and 0.05% w/v SDC) in a 2:1 ratio. The pH was adjusted using HCl, resulting in a purple top phase solution containing (6,5). Impurities in the (6,5) solution were removed using tangential flow filtration with a hollow fiber membrane (100K, mPES, C02-E100-05-N, Repligen). The 1% SC solution was employed for washing (6,5) and replacing the surfactant coating on (6,5) from SDC to SC, ultimately obtaining single chirality (6,5) in 1% SC.

Method S2. *<u>Investigation of the environmental effect on (6,5) emission</u>*

To assess the potential impact of cellular ingredients and proteins on the (6,5) surface, and their influence on its fluorescence properties, the fluorescence spectra of (6,5) were examined under different conditions. The $(GT)_{20}$-coated (6,5) sample suspension was initially diluted separately with full medium (DMEM with 10% FBS) and phosphate buffer (0.05M). After a 16-hour incubation, the fluorescence emission of (6,5) was measured using a spectrometer, with the emission intensity normalized to the maximum.

For the cellular condition, RAW264.7 macrophages (60001, BCRC, Taiwan) were cultured in a 6-well plate for one day, followed by co-incubation with (6,5) for 30 minutes. After washing the cells with DPBS three times, the cells were collected by scraping and measured using a spectrometer. This experimental setup allowed for the examination of (6,5) fluorescence in the presence of cellular

components and proteins, providing insights into potential interactions and alterations in the fluorescence spectrum.

Method S3. <u>*Resuspension of the intracellular (6,5) for spectral measurements*</u>

To completely individualize and suspend intracellular (6,5) in solution, a 1% SDC (sodium deoxycholate) solution was employed to lyse the cells and resuspend (6,5) through tip sonication (1W, 10s) under ice bath conditions. The resulting solution was subsequently analyzed using our home-built SWIR fluorometer and spectrophotometer.

Method S4. <u>*Visualization of (6,5) uptake in RAW 264.7 macrophages*</u>

The (6,5) uptake by macrophages was investigated using our home-built SWIR fluorescence microscope. Murine macrophage RAW 264.7 cells were initially seeded in an 8-well chambered coverglass (Nunc Lab-Tek, Thermo) at a density of 30,000 cells per well for 24 h. Subsequently, the cells were treated with $(GT)_{20}$-coated (6,5) at a concentration of 300 µg L^{-1} for 15, 30, and 60 min. After co-incubation, the cells were washed with DPBS three times and imaged using our SWIR fluorescence microscope system, equipped with a 561 nm LED light source and a 60× objective lens.

Method S5. <u>*Effect of formalin fixation on autofluorescence signals detected by flow cytometry*</u>

RAW 264.7 macrophages, directly collected from the cell flask, were washed with DPBS and fixed with formalin for 10 min. The fixed cells were then washed three times with DPBS using a benchtop centrifuge (DPBS, 300 ×g, 5 min) and then measured by the flow cytometer. The flow rate of the measurements was set to a medium rate of 30 µL min^{-1}, resulting in a cell count rate of ~1000 cell s^{-1}. Signals from FSC, SSC, and (6,5) channels were acquired.

Method S6. <u>*Preparation of HiPco SWCNTs for aggregation-state examination*</u>

HiPco powder was suspended in a 1% SDC aqueous solution using tip sonication, followed by ultracentrifugation at 280,000 ×g for 1.5 h. The supernatant was collected as the individualized sample, and the pellet was collected as the agglomerated sample. SWCNT particles (5 µL) were dropcast onto a coverslip and affixed to a microscope slide with tape. Fluorescence images of SWCNTs were acquired using our custom-built SWIR microscope system, featuring an inverted Nikon Eclipse Ti2 microscope with a 60× magnification water objective (CFI Plan Apo IR 60XC WI). The samples were excited with a Chrolis™ 6-wavelength high-power LED (365 to 780 nm). Fluorescence images passed through the broadband mode of a Photon etc hyperspectral filter and were captured using a Princeton Instruments

NIRvana LN camera in NDRO (Non-Destructive Readout) mode, with an exposure time of 90 seconds. MATLAB was employed for image processing.

Method S7. *Evaluation of the cytotoxicity of $(GT)_{20}$-coated (6,5)*

The toxicity effect of $(GT)_{20}$-coated (6,5) was evaluated using the PrestoBlue HS assay (P50200, Invitrogen™). RAW 264.7 cells were initially seeded into a 96-well black plate (5000 cells well^{-1}, 100 μL well^{-1}) for 10 h. Subsequently, 100 μL of DMEM medium solutions with various concentrations of (6,5) (25-3200 μg L^{-1}) were added to the wells. After 12 h of coincubation, cells were washed with DPBS, followed by the addition of fresh medium (90 μL well^{-1}) for an additional 48 h of incubation. PrestoBlue HS was then added and incubated for 100 min. Fluorescence measurements were conducted with 520 nm excitation and 580-640 nm emission using a GloMAX plate reader.

Discussion

Discussion S1. *Determination of aggregation states of HiPco SWCNTs using SWIR microscope*

The dispersity of HiPco SWCNTs, whether individualized or aggregated, was assessed using SWIR fluorescence microscopy with 561 nm laser excitation. Figure S1 shows the comparison of the individualized and agglomerated samples. The individualized SWCNTs (left) appear as small dots in the image, while the agglomerated SWCNTs (right) form large bright areas. The lengths of the individualized SWCNTs are ~100-200 nm, much shorter than the diffraction limit (~600 nm) of the light. Microscope images can only distinguish particles larger than the diffraction limit. Therefore, small SWCNT bundles may appear similar in size to the individualized ones.

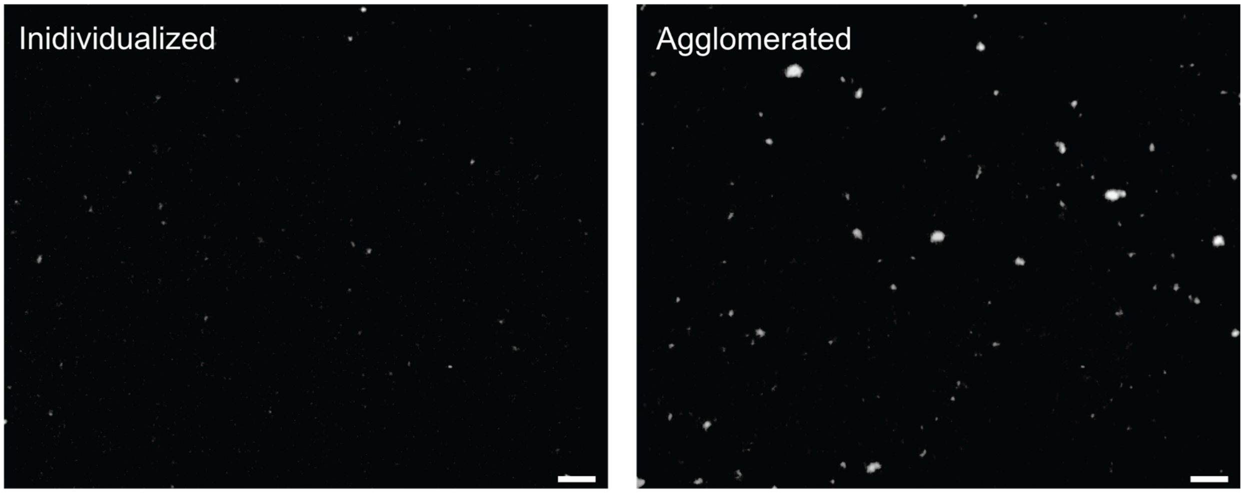

Figure S1. SWIR fluorescence images of HiPco SWCNTs either in individualized or agglomerated states. Scale bar represents 25 µm.

Discussion S2. *Determination of aggregation states of (6,5) using skewness analysis*

Variance spectrometry was employed to identify the hetero-aggregates of SWCNTs by detecting the presence of off-diagonal signals in a 2D covariance map. Further analysis of the third moments of intensity distributions, or skewness, enabled the detection of aggregates of single-chirality SWCNTs. To validate the feasibility of our skewness analysis, two HiPco SWCNT samples, one in an aggregated state and the other individualized, were compared by examining skewness spectra and covariance maps.

HiPco SWCNTs consist of multiple SWCNT species (Figure S2a). The S_{11} emission of (6,5) (980-1000 nm) from the agglomerated SWCNT sample exhibited larger fluctuation compared to the individualized one (Figure S2b). The off-diagonal signal was prominently present in the covariance map of the agglomerated SWCNT group compared to the individualized one (Figure S2c), confirming the existence of aggregations. The characteristic variance intensity at the S_{11} wavelength revealed that the emission signal fluctuation mainly originated from (6,5), (8,4), and (7,6) (Figure S2d), matching their S_{11} emissions at ~980 nm and 1,110-1,120 nm in SDC condition. Further analysis of the skewness value from the fluorescence spectra indicated that a high skewness value (over 0.3) signified that the SWCNTs were in an aggregated or bundled state (Figure S2e). Overall, this experiment qualitatively compared the off-diagonal peak intensity from the covariance map and the peak value from the skewness spectrum, confirming the success of the skewness measurement of well suspended, single-chirality samples.

Figure S3 presents data related to variance spectrometry for $(GT)_{20}$-coated (6,5). The normalized absorption spectrum of the well-individualized (6,5) sample indicates a high purity (6,5) sample, with some (9,1) absorption observed (see Figure S3a). In contrast, the agglomerated sample shows a higher background level and an additional shoulder to the right of the S_{11} transition, both attributed to the aggregation/agglomeration of SWCNTs. The fluorescence spectrum of the well-dispersed SWCNT sample exhibits a prominent (6,5) S_{11} emission and a strong sideband at ~1,160 nm. The relative intensity of this sideband serves as an indicator of the extent of ssDNA coverage on the SWCNT surface (see Figure S3b). We constantly observe that a better surfactant/DNA coating results in a lower sideband, suggesting its reduced correlation with chirality impurity. An extra shoulder at ~1,050 nm is also observed, possibly originating from (7,5) emission. Although the percentage of (7,5) should be extremely low, as evidenced by the absorption spectrum of the individualized sample, we posit that the appearance of the extra (7,5) emission band stems from the severe aggregation of the SWCNT sample, leading to energy transfer of excitons from (6,5) to (7,5) and amplifying the (7,5) signal ratio. High-intensity events are observed in the series of acquisition frames for the agglomerated sample (see Figure S3c), providing direct evidence of the existence of agglomerated particles. Figure S3d displays a more symmetric

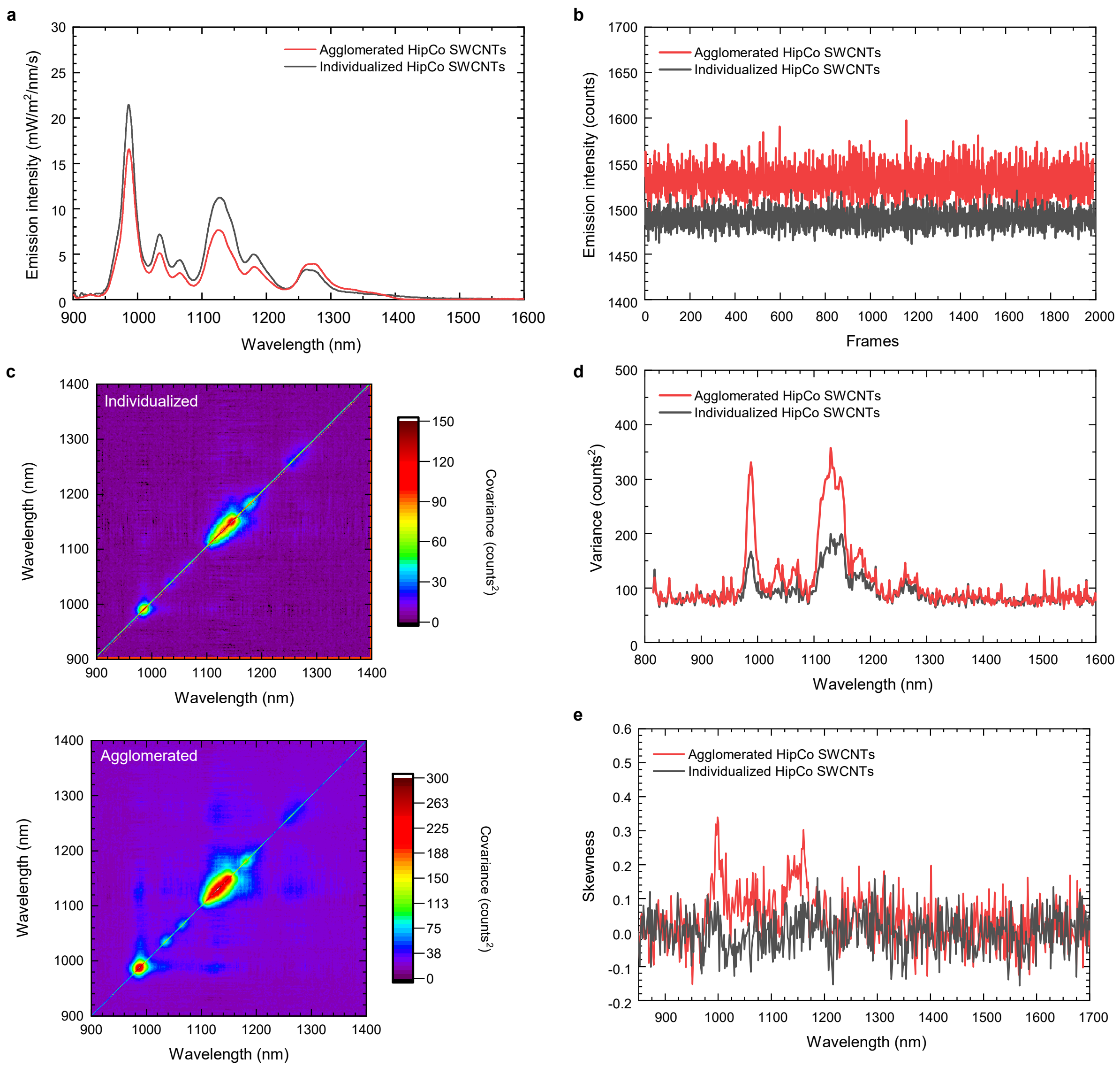

Figure S2. Skewness analysis of HiPco SWCNTs using variance spectrometry. (a) Fluorescence spectra of HiPco SWCNTs either in agglomerated and individualized states. (b) S_{11} emission intensity with respect to the acquisition frame. (c) Covariance map, (d) variance spectra, and (e) skewness spectra of HiPco SWCNTs in either agglomerated or well-suspended states.

distribution for the individualized sample. An essential observation is the variance spectra, where the appearance of a variance peak indicates successful observation of nanoparticle fluctuation. This is crucial for using skewness spectra to demonstrate a well-individualized sample. Both agglomerated and individualized samples exhibit reasonable variance values, as shown in Figure S3e and 3f. We note that the signal-to-noise ratio of the variance spectrum for the individualized sample in our case is smaller than that in the literature. While various factors can contribute to different variance values, we believe that our shorter SWCNT length, prepared through longer sonication time and higher *g* ultracentrifugation, is the primary influencing factor.

An intriguing spectral shape is observed in Figure 1e, deviating somewhat from the expected pattern. Instead of a narrow (6,5) peak at ~1,000 nm, a broader peak spanning from 950 to almost 1,400 nm is evident. This broader peak suggests the agglomeration of (6,5) with residual species of larger diameters. It is important to note that, due to the exceedingly small percentage of these larger diameter species, both the variance and skewness for these components become extremely high.

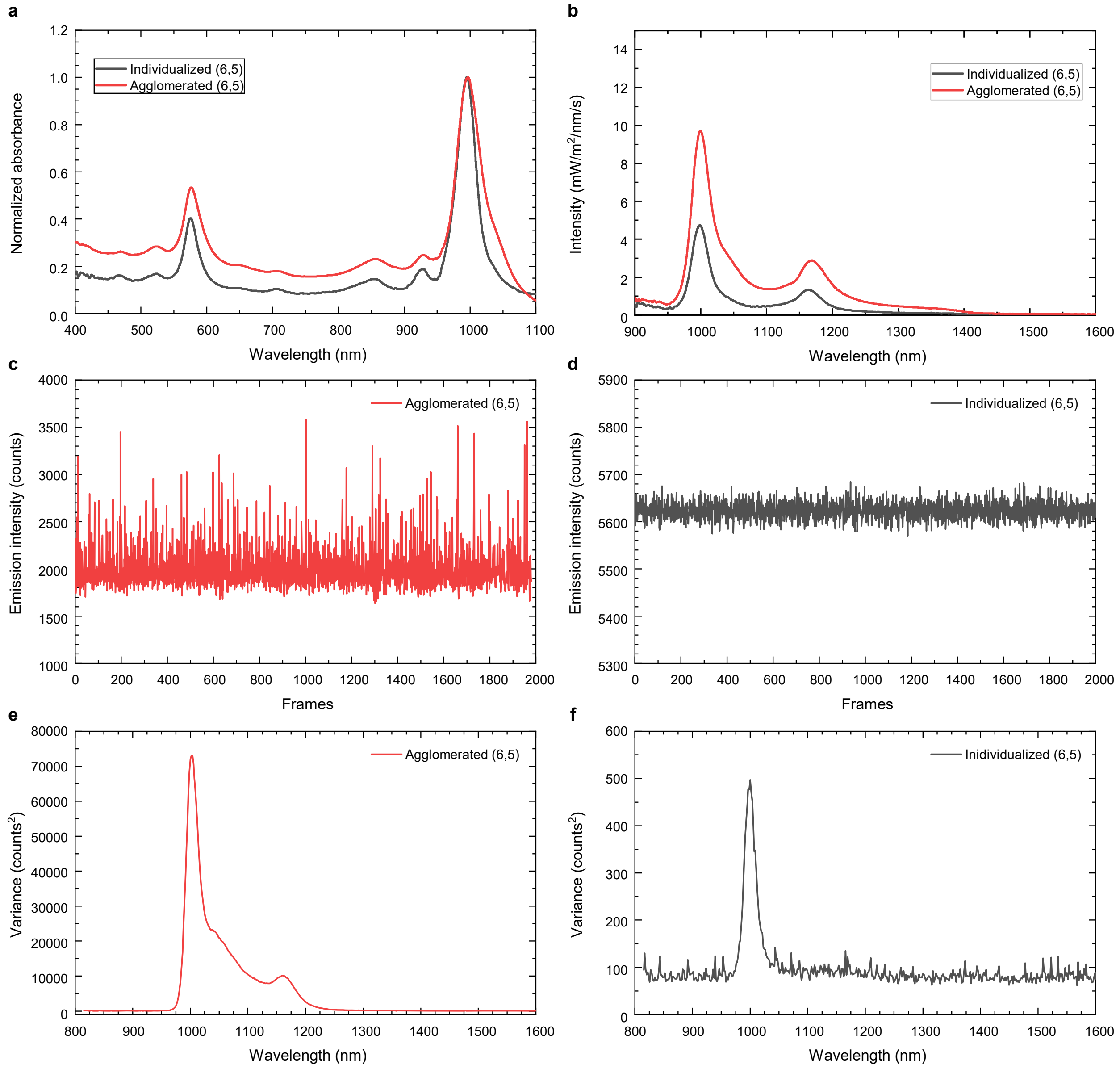

Figure S3. Skewness analysis of (6,5) by variance spectrometry. (a) Absorption and (b), fluorescence spectra of agglomerated and individualized (6,5). (c-d) S_{11} emission intensity regarding to frames from (c) agglomerated and (d) individualized (6,5). (e-f) Variance spectra of (e) agglomerated and (f) individualized (6,5).

Discussion S3. <u>*Time-dependent (6,5) uptake of RAW macrophages*</u>

$(GT)_{20}$-coated (6,5) can be recognized and engulfed by RAW macrophages through endocytosis. However, the high concentration of proteins and sugars in the medium might lead to the formation of (6,5) corona or agglomerates over time, affecting the extent of uptake. Therefore, the uptake condition with respect to different treatment periods has to be investigated.

As shown in Figure S4, some (6,5) nanoparticles (NPs) are observed in the macrophages within a 15-min treatment period. More (6,5) NPs are found in the macrophages after a 30-min treatment. However, when the treatment time is increased to 60 min, big clumps of signals, probably due to the loose agglomerates of (6,5), on the macrophage membrane are observed. Therefore, the (6,5) coincubation time was set to 30 min for all cell experiments in this work.

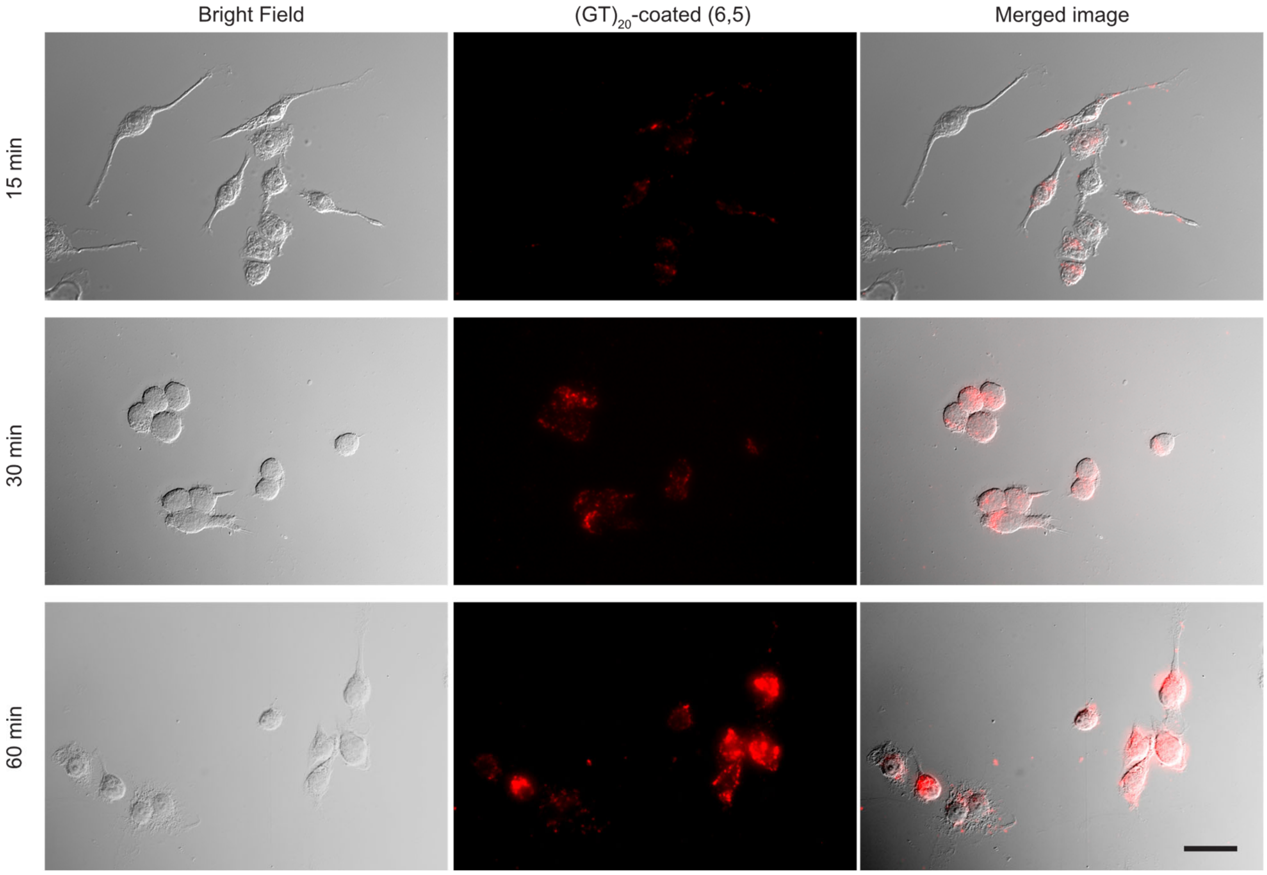

Figure S4. SWIR fluorescence microscopic images of macrophages treated with 300 µg L^{-1} (6,5) for 15, 30 and 60 min. Several (6,5) agglomerates were found on the cell membrane in the case of 60-min treatment. Scale bar is 20 µm.

Discussion S4. <u>Fixation effect on the fluorescence detection of flow cytometry</u>

Fixation is a standard procedure in preparing flow cytometry samples to extend storage time and provide flexibility for characterizations. Formalin is commonly employed for cell or tissue fixation to crosslink the cell membrane structure, potentially impacting the measurement of SWCNT signals.

The impact of formalin fixation is illustrated in Figure S5. The mean fluorescence intensity (MFI) in (6,5) channel exhibited a slight decrease of approximately 24.7% after fixation. However, no significant differences were observed between the fixed and unfixed groups in SSC or FSC versus SWCNT signal plots (Figure S5b and 5c). These findings suggest that the reduced MFI in the cell background signal may arise from other factors, such as the molecular structure of membrane components, rather than a change in the size and complexity of the cells. In summary, the influence of fixation should be considered in flow cytometry, and in our case, we found no significant concerns.

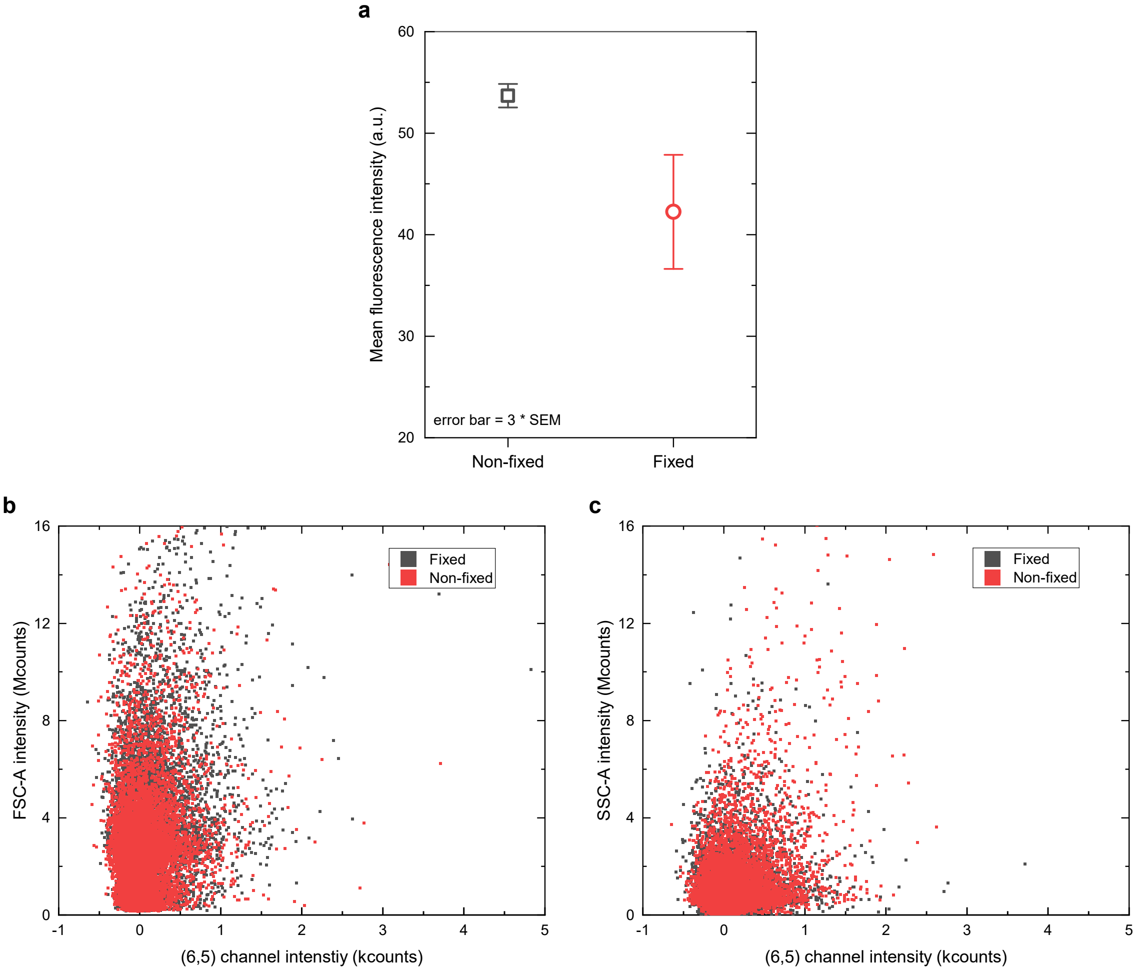

Figure S5. Fixation effect on mean fluorescence intensity of (6,5) in flow cytometry measurement. (a) MFI of macrophages with and without formalin fixation. (b) Scatter plot of FSC vs (6,5) channel intensity. (c) Scatter plot of SSC versus (6,5) channel intensity.

Discussion S5. *Gating strategy and the comparison of (6,5) levels in singlet and whole populations*

Two additional detectors are installed in the flow cytometer to collect forward and side scatter signals (FSC and SSC). The FSC and SSC are measured at 180° and 90° relative to the excitation position, respectively. The signal acquisition is continuous and therefore results in a profile in the plot of signal intensity (voltage) with respect to time, as shown in Figure S6a, when a cell event happens.[2] FSC intensity has a positive correlation to the cell volume,[3] while SSC intensity reflects the morphological complexity of a cell. Height and area of the voltage-time profiles provide additional cell information such as intactness and aggregation. In Figure 1g, cell debris exhibits lower FSC due to its smaller size and lower SSC due to its lower morphological complexity. Therefore, we exclude the lower-left population, gating the remaining intact cells labeled as live cells. In Figure 1h, FSC-H and FSC-A should be proportional if all cells are individualized, indicating similar voltage-time profile for all cells. For a cell-cluster event, the signal area can be proportional to the cell number while signal peak (height) remains the same or similar. As a result, the event location will shift toward the lower right in the FSC-H vs FSC-A plot. A clear doublet cell cluster population is observed at the lower right of the singlet cell population in this case.

The MFI ratio between singlet cells and the entire population was calculated and presented in Figure S6b. This ratio, ranging from 0.6 to 0.8, indicates that the uptake patterns of (6,5) in both singlet cells and cell agglomerated are similar, with the majority (60-80%) of the (6,5) signals originating from singlet cells. We further define a concentration factor $\kappa(D)$ as follows:

$$\kappa(D) = \frac{\overline{m}(D)}{\sigma_{\text{cell}} D} = \frac{\overline{m}_{\max}}{\sigma_{\text{cell}}} \frac{D^{n-1}}{k_{\text{M}}^{n} + D^{n}}$$

Here, the values of $\overline{m}_{\max}/\sigma_{\text{cell}}$, k_{M}, and n are 20.2 mg L^{-1}, 0.1982 mg L^{-1}, and 1.66, respectively. The $\kappa(D)$ can then be estimated for all known (6,5) doses D. As shown in Figure S6c, the concentration factor $\kappa(D)$ increases drastically and reaches a maximum of 52 at 154.3 μg L^{-1}, followed by a slow decrease.

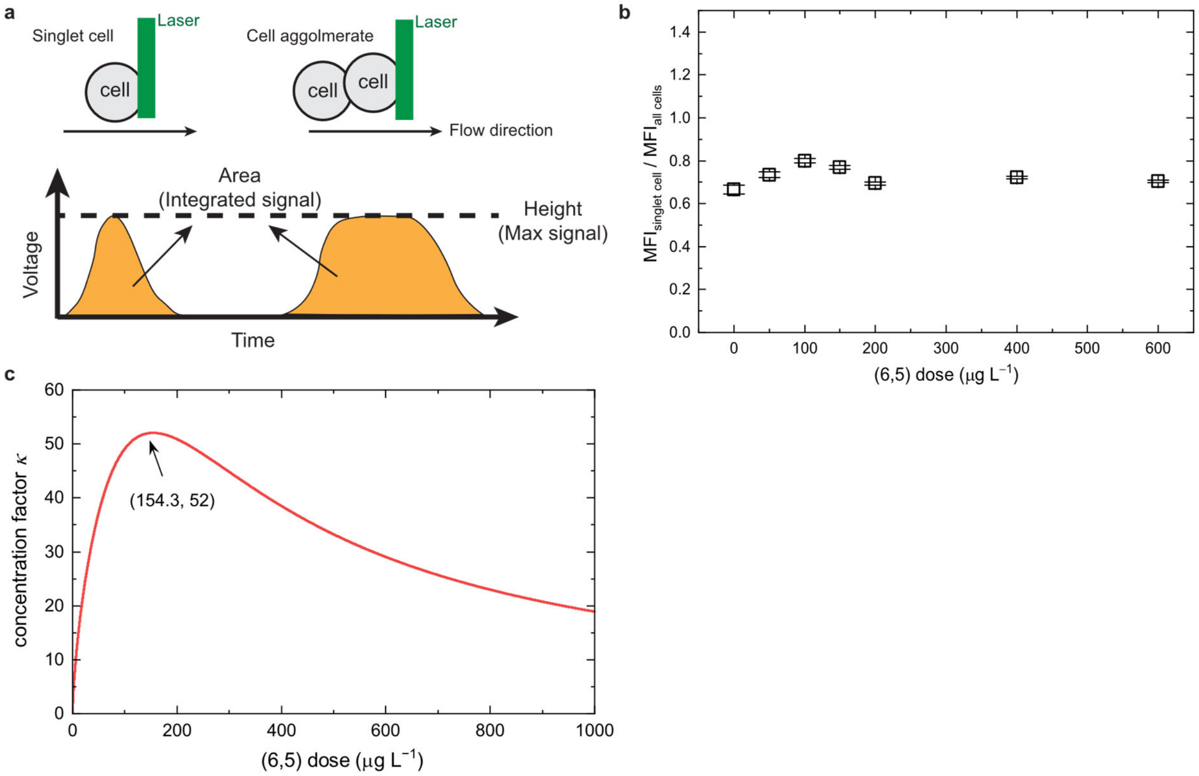

Figure S6. (a) Voltage pulse diagram of detected signal from a flow cytometer. (b, c) Comparison of (6,5) levels in singlet and whole cell populations. (b) Ratio of (6,5) MFIs in the singlet cell population to that in whole cell population measured by flow cytometry. (c) The concentration factor κ with respect to (6,5) does. The peak concentration factor κ_M is at 154.3 μg L^{-1}. Error bars represent SD from triplicate experiments.

Discussion S6. *<u>(6,5) dose-dependent MFI fitted with Hill equation</u>*

The bulk spectrometry measures ensemble average of all (6,5) NPs in the sample. Therefore, no gating (all cell events) should be applied to correctly correlate the results from flow cytometry and bulk spectrometry, as opposed to the data shown in Figure 1g and 1h. The mean fluorescence intensities of (6,5) from the whole cell population are included to obtain the (6,5) intensity distribution in Figure S7a, and the results are listed in Table S1. Figure S7b shows the MFI with respect to the treated (6,5) concentration with and without gating.

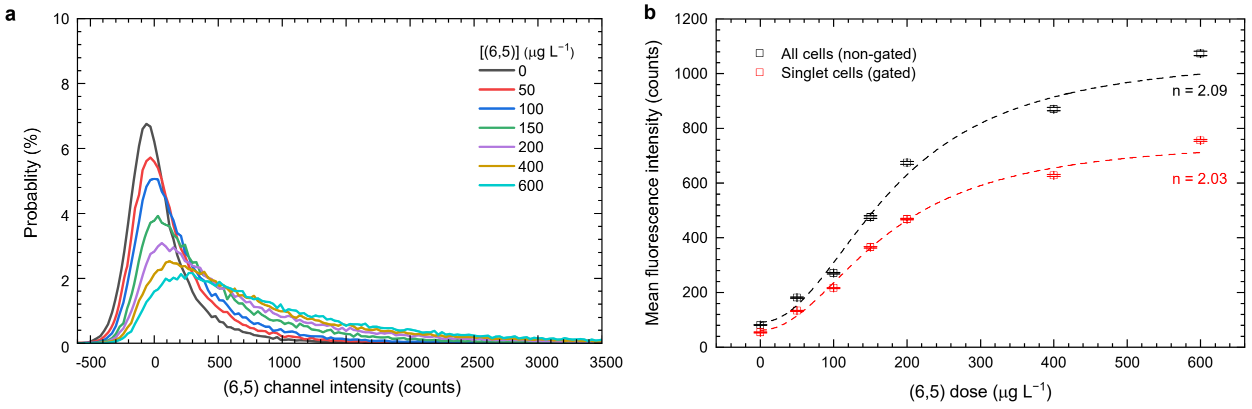

Figure S7. Intracellular (6,5) intensity in the cells with various dose concentrations. (a) Intensity distribution in (6,5) channel without gating. (b) MFI of (6,5) intensity with respect to the treated (6,5) concentration. Short dashed lines represent the fitted Hill equation.

Table S1. Summary of Mean, median, FWHM, SD and SE of fluorescence signals of the samples measured by our SWIR flow cytometry.

Dose (μg L^{-1})	Mean(counts)	Median (counts)	FWHM	SD	SE
Total cell population (non-gated)					
0	81.1	10.3	331.3	240	1.6
50	181.1	75.8	372.7	540	2.4
100	270.6	135.1	434.8	730	2.9
150	475.4	268.1	538.3	1000	4.7
200	674.3	412.3	703.9	1300	5.1
400	870.3	550.8	952.3	1500	6.0
600	1073.7	704.1	1076.6	2000	8.0
Singlet cell population (gated)					
0	54.0	-5.1	321.0	270	1.3
50	133.0	62.4	379.4	310	1.8
100	216.5	125.6	437.7	370	1.9
150	365.4	246.2	554.5	480	2.8
200	468.3	338.5	700.4	560	2.8
400	628.3	478.8	904.6	670	3.4
600	755.7	598.8	963.0	720	3.6

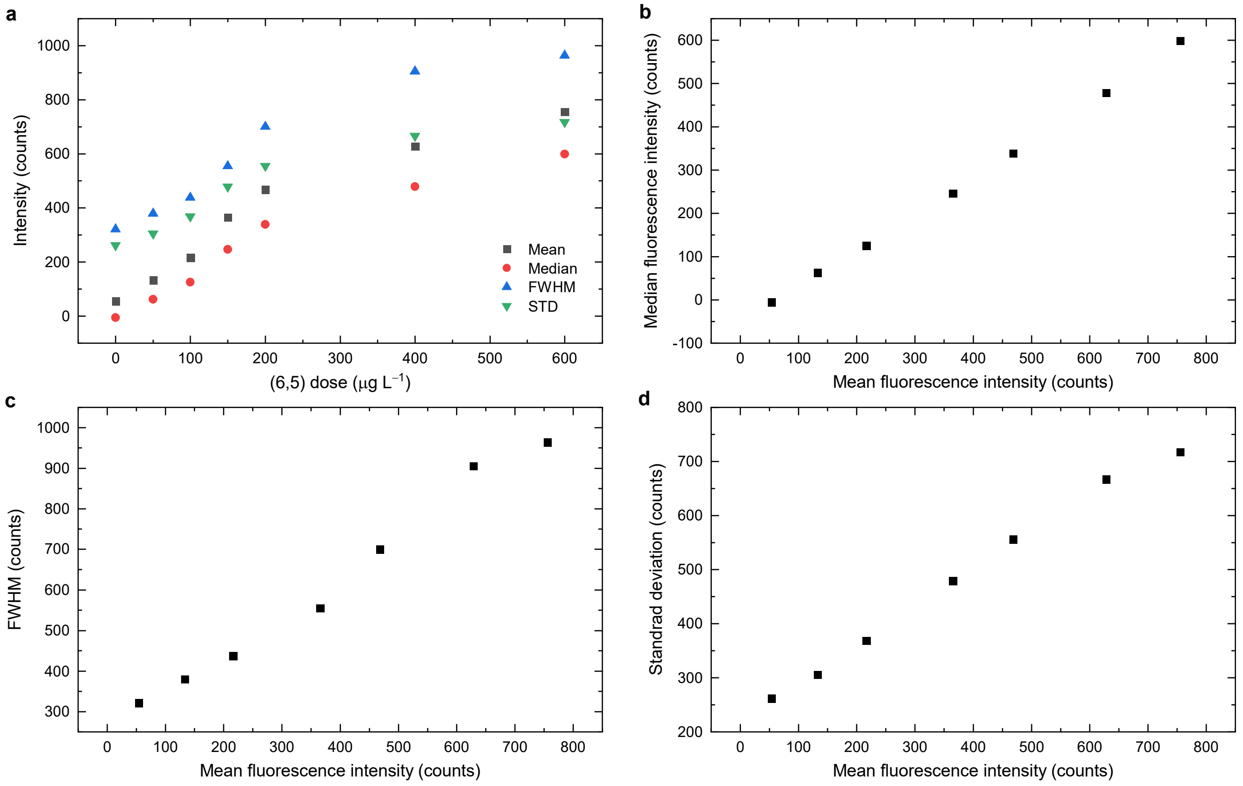

Figure S8. Relation of analytical results of singlet cell population from Table S1.

The intracellular (6,5) level exhibits a monotonically increasing trend with (6,5) dosage, gradually reaching a plateau beyond a dosing concentration of 200 μg L^{-1}. The Hill equation, presented below, is employed to model the mean fluorescence intensity (MFI) $\bar{I}$ as a function of the treated (6,5) concentration D. The "Hill1" function in OriginPro 2021 was utilized for fitting our dataset

$$\bar{I} = \bar{I}_{\mathrm{min}} + (\bar{I}_{\mathrm{max}} - \bar{I}_{\mathrm{min}}) \frac{D^n}{k_{\mathrm{M}}^n + D^n} \quad (1)$$

The fitted parameters and statistics were listed in Table S2, where n is the Hill coefficient, and k_{M} is the Michaelis constant. The Hill coefficient, approximately equal to 2, indicates the positively cooperative nature of the MFI concerning the treated (6,5) concentration. This suggests that (6,5) transportation primarily occurs through an active internalization process, such as the receptor-mediated endocytosis pathway. Note that this result slightly differs from that in Figure 2c, as the function's codomain is MFI instead of cellular (6,5) mass m in this case. The cellular (6,5) mass m is proportional to the MFI $\bar{I}$ with a multiplication factor γ as described in Eq. 2 in the main text. The results therefore would be exactly identical if no deduction error occurred during the conversion of MFI to cellular (6,5) mass. The Michaelis constant k_{M} serves as a measure of the macrophages' (6,5) uptake capability, represented as

the treated (6,5) concentration required for half-maximum uptake. The $\bar{I}_{\mathrm{max}}$ of the non-gated case is extensively higher than that of the gated one, indicating that cell clusters may accumulate extra (6,5). We suspect that, in the cell clusters, some (6,5) agglomerates grab cells together, leading to extra (6,5) fluorescence signal.

Table S2. Fitted parameters from Figure S7b.

Exp. groups	Non-gated	Gated
$\bar{I}_{\mathrm{min}}$	92 ± 95	62 ± 64
$\bar{I}_{\mathrm{max}}$	1,070 ± 200	764 ± 92
k_{M}	182 ± 40	173 ± 24
n	2.1 ± 1.0	2.0 ± 0.7
R^2	0.98596	0.9928

Discussion S7. <u>*Relation of autofluorescence and (6,5) intensity spreading*</u>

Figure S9a illustrates the spread of intensities from non-stained cells, which may arise from either detector noises or varying levels of autofluorescence among different cells. Considering that the size and complexity of cells contribute to different autofluorescence levels, we calculated the correlation between FSC and (6,5) channel intensities of non-stained cells. A linear regression was performed on the data, assuming that larger cells exhibit both stronger autofluorescence and greater forward scattering. Figure S9b presents the comparison of data before and after subtraction of autofluorescence, which is predicted from the regression line. The Pearson Correlation Coefficient (PCC) is used to assess the correlation between (6,5) channel and FSC intensities. The PCC decreases from 0.2 to near 0 after autofluorescence subtraction. However, as shown in Figure S9c, the probability histogram of (6,5) channel intensity displays a broader width for the autofluorescence-subtracted case than for the non-autofluorescence-subtracted one, indicating that system noises, rather than autofluorescence, dominate the observed data spreading.

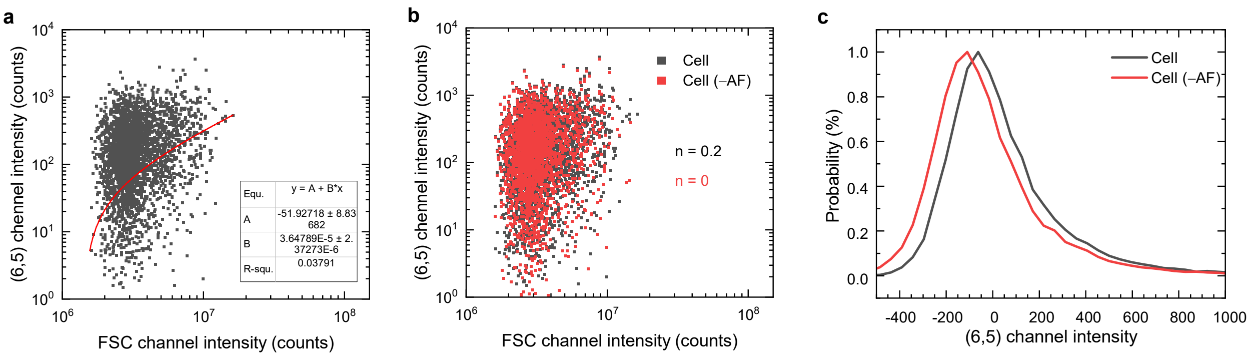

Figure S9. Analyses of signal spreading in (6,5) channel. (a) Scatter plot of (6,5) channel intensity with respect to FSC intensity for unstained cells (control). Red curve represents the linear regression line of the data. (b) Comparison of the data with and without autofluorescence subtraction. *n* stands for the Pearson Correlation Coefficient. (c) Intensity distributions of (6,5) channel intensity before and after autofluorescence subtraction.

Discussion S8. *<u>Effect of the residual cellular components on (6,5) spectroscopic measurements</u>*

As compared to (6,5) in phosphate buffer, the maximum (6,5) emission wavelength in full medium exhibited a 3 nm redshift from 999 to 1,002 nm (see Figure S10a). This shift might result from displacement of ssDNA coverage into medium proteins.[4] On the other hand, intracellular (6,5) displayed a similar redshift in emission to a wavelength of 1,002 nm (Figure S10b). However, strong scattering from cellular components introduced additional background emission, complicating the quantification of (6,5).

To fully liberate and suspend intracellular (6,5) in solution, cell lysis and resuspension were performed using 1% SDC with tip sonication. The shapes of the fluorescence spectra of (6,5) from lysed cells and clean surfactant are very similar (see Figure S10c), while the absorption spectrum of (6,5) from lysed cells gives extra background compared to that from clean surfactant (see Figure S10d). These results indicate that the emission intensity is a more appropriate physical quantification for accurately determining the (6,5) amount in the cells.

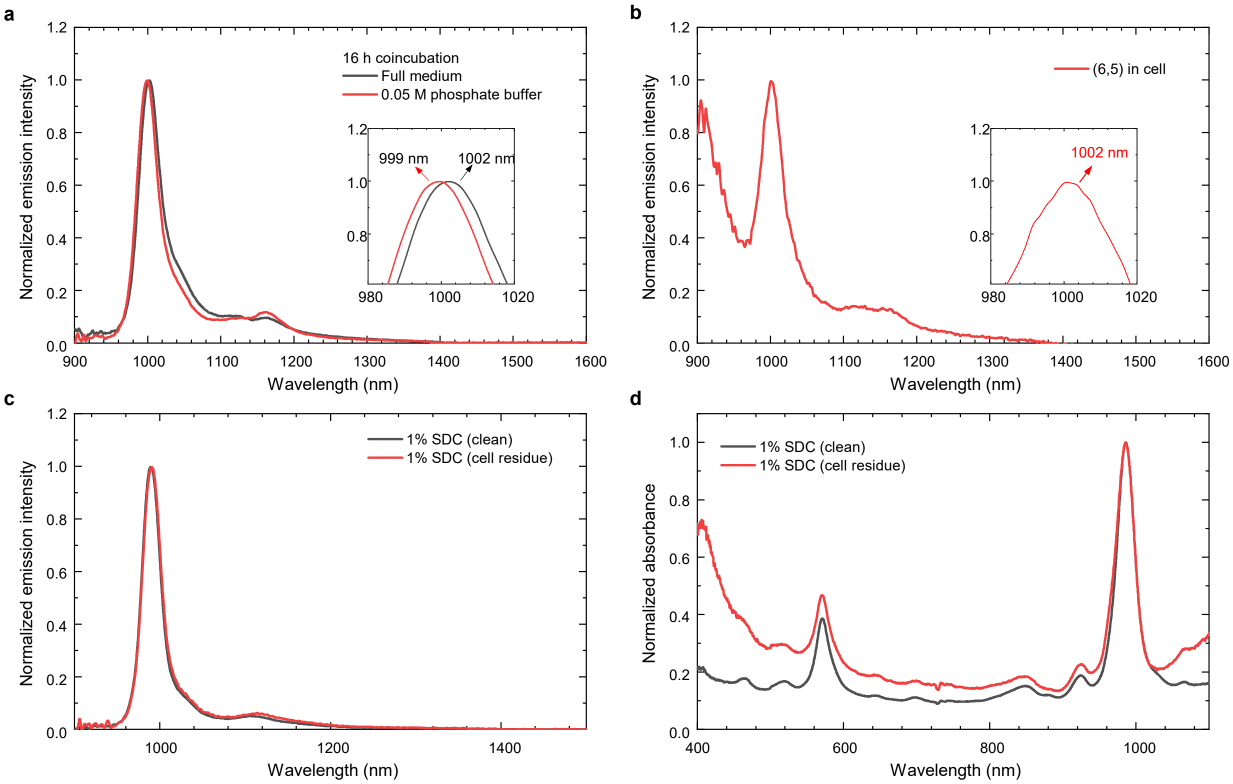

Figure S10. Fluorescence and absorption spectra of (6,5) in full medium, phosphate buffer, and macrophages. Fluorescence spectra of $(GT)_{20}$-coated (6,5) either (a) in the full medium and phosphate buffer or (b) in macrophages. Inset figures illustrate the peak position of (6,5) characteristic emission peak. (c) Emission and (d) absorption spectra of (6,5) in 1% SDC condition with or without the cell residue.

Discussion S9. *Background level of cellular autofluorescence*

Autofluorescence levels of macrophages in each flow cytometry channel were measured by setting the channel voltage gain to the maximum (3000) for the measurements. Fresh unstained macrophages were collected, washed, and measured by flow cytometry directly. It is observed that the autofluorescence is lower for longer excitation wavelengths such as 633 nm. For 405 and 488 nm excitations, the autofluorescence is stronger for green emission around 525 nm. Interestingly, for the 561 nm excitation, the autofluorescence is stronger for longer emission wavelengths, except 900 nm. Note that the intensities are not calibrated by the spectral efficiency of the APD, but it might not be an issue because the APD efficiency remains relatively unchanging from 400-900 nm.

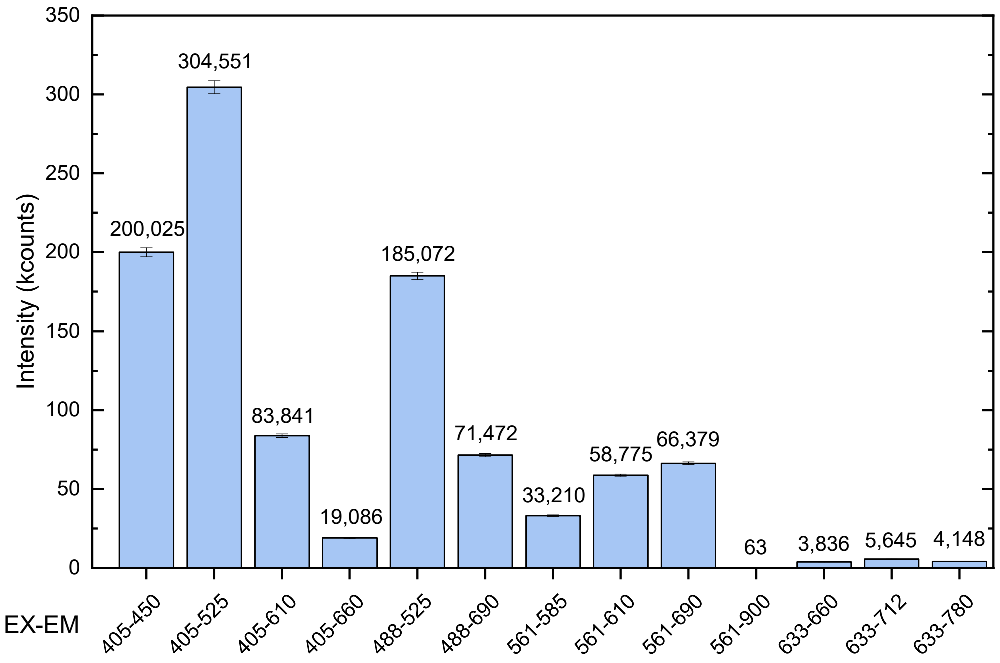

Figure S11. Autofluorescence levels of RAW 264.7 from each fluorescence channel of the flow cytometry.

Discussion S10. <u>*Deduction of (6,5) concentration using its fluorescence*</u>

The first part of this section details the procedure for determining the (6,5) mass concentration as a function of (6,5) fluorescence measured using bulk spectrometry. The stock solution of $(GT)_{20}$-coated (6,5) for this experiment was prepared by diluting the $(GT)_{20}$-coated (6,5) solution with a 1% w/v sodium deoxycholate (SDC) solution to OD ~1.0. Several samples with lower (6,5) concentrations were then prepared by sequentially diluting the stock solution with 1% SDC. Absorption and SWIR fluorescence spectra of these samples were measured. A 561-nm CW laser was used as the excitation source for SWIR fluorescence measurements.

Figure S12a and 12b show the absorption and fluorescence spectra of (6,5) with different dilution factors, respectively. Figure S12c shows the S_{11} peak absorption $A_{(6,5)}$ (~987 nm) with respect to the S_{11} peak emission intensity I_{spec} (~989 nm) of (6,5), and the data are linearly regressed with an R^2 value of 0.99942, indicating that photon reabsorption by (6,5) and photon scattering can be neglected.[5] To deduce the (6,5) mass concentration C_m, the extinction coefficient of (6,5), $\varepsilon_{(6,5)}$, is required, and the Beer–Lambert law can be exploited:

$$A_{(6,5)} = \varepsilon_{(6,5)} b C_m \tag{2}$$

where b is the path length of light through the sample. The value of $\varepsilon_{(6,5)}$ is set to 0.55 mL μg^{-1} cm^{-1}, as reported in a previous study.[6] b is set to 1 cm because a 1 cm fused silica cuvette was used. Thus, the (6,5) mass concentration can be obtained from the following equation:

$$C_m = \frac{A_{(6,5)}}{\varepsilon_{(6,5)} b} = \frac{A_{(6,5)}}{0.55\ \mathrm{mL\ \mu g^{-1}\ cm^{-1}}}$$

Figure S12d illustrates the (6,5) mass concentration C_m with respect to the measured fluorescence intensity I_{spec}. The fluorescence intensity should be proportional to the (6,5) concentration, and the linearly fitted line also results in a high R^2 value. The relation between C_m and I_{spec} can be described using the following equation:

$$C_m = \rho_{\mathrm{spec}} I_{\mathrm{spec}} \tag{3}$$

where ρ_{spec} is the slope of the function, or the (6,5) mass concentration per acquired fluorescence count. The fitted value of ρ_{spec} is then 1.783×10^{-2}. Therefore, the (6,5) mass concentrations from all samples can be deduced from their measured fluorescence intensity.

Figure S12e and h shows the fluorescence spectra of the seven (6,5) samples (extracted from the cells) aimed for instrument calibration. The fluorescence intensity is then converted into the (6,5) mass

concentration C_m using Eq. S3, and the corresponding plot of (6,5) mass concentration with respect to the treated (6,5) concentration is shown in Figure S12g.

Thus far, the calculated concentrations are from the bulk samples, lacking cellular information to correlate them to cytometry results. We need specific cell number n_{cell}, or cell number per unit of volume, for obtaining the cellular (6,5) mass concentration $\bar{m}$. The specific cell number n_{cell} is defined as:

$$n_{\text{cell}}(D) = \frac{N(D)}{V(D)} \tag{4}$$

where N is the total cell number in the sample, V is the volume of the sample, and D is the dose of the (6,5) during coincubation. The measured and deduced results are listed in Table S3. The cellular (6,5) mass $\bar{m}(D)$ is the (6,5) mass concentration divided by the specific cell number $n_{\text{cell}}(D)$:

$$\bar{m}(D) = \frac{C_m(D)}{n_{\text{cell}}(D)} = \frac{\rho_{\text{spec}} I_{\text{spec}}(D)}{n_{\text{cell}}(D)} \tag{5}$$

Note that the cellular (6,5) mass m is a function of the (6,5) dose D, but has not been written in an explicit form yet. Figure S12h shows the plot of cellular (6,5) mass $\bar{m}(D)$ with respect to the treated (6,5) concentrations D. In the main text, we empirically fit this dataset using the Hill equation, as shown in Figure 2c.

Table S3. Deduction of specific cell number.

Volume	Total volume (μL)	For bulk spectrometry (μL)	For cell counting (μL)	For flow spectrometry (μL)	Original n_{cell} (10^9 L^{-1})$	n_{cell} for bulk spectrometry (10^9 L^{-1})*
NO1	105	80	5	20	3.54	1.42
NO2	125	100	5	20	4.23	2.12
NO3	130	105	5	20	3.33	1.75
NO4	120	95	5	20	4.47	2.12
NO5	125	100	5	20	3.28	1.64
NO6	124	99	5	20	3.35	1.66
Cell only	975	950	5	20	5.72	5.72

$ The specific cell number n_{cell} was measured using Invitrogen Countess 2.

* Samples for bulk analysis were diluted to 200 μL. The values were obtained from original n_{cell} multiplied by the dilution factors, or original n_{cell} (10^9 L^{-1}) × volume for bulk spectrometry (μL) / 200 (μL). The cell only group did not perform the dilution because the volume exceeds 200 μL.

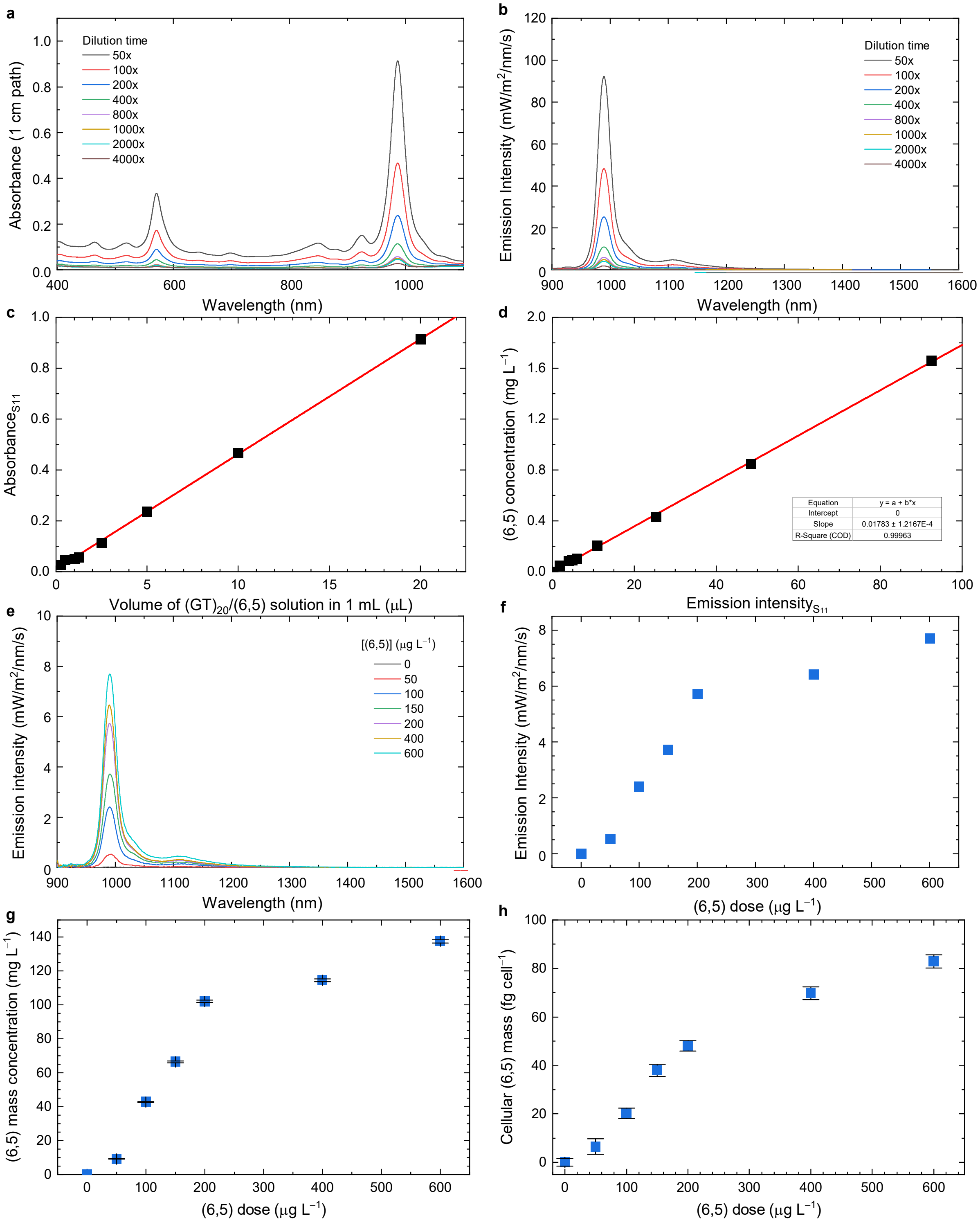

Figure S12. Quantification calibration procedure. (a) Fluorescence and (b) absorption spectra of (GT)$_{20}$-coated (6,5) in 1% SDC solution measured using bulk spectrometry. (c) Relation between absorption and fluorescence peak intensity at (6,5) S_{11} transition. (d) Relation between (6,5) concentration and fluorescence peak intensity. (e) Fluorescence spectra of the seven samples used for instrument quantification calibration. (f) S_{11} emission peak intensity of in vitro samples with respect to (6,5) dose. (g) C_m versus D. (h) Relation of cellular (6,5) mass to (6,5) dose, calculated from $C_m(D)$ and specific cell number $n_{\text{cell}}(D)$ of each experimental group.

Discussion S11. *Factors that influence the autofluorescence level in flow cytometry*

Figure S13a highlights distinct MFI values in the (6,5) channel for different cell lines, including mouse macrophage RAW 264.7, human breast cancer MCF-7, and mouse bladder cancer MB49. This discrepancy is likely attributed to variations in cell diameters and the complexity of the inner cellular structure, with approximately a 45% difference observed between RAW and MCF-7.

Furthermore, the impact of three different flow rates—10 μL min^{-1} (low), 30 μL min^{-1} (medium), and 60 μL min^{-1} (high), corresponding to ~300, 1,000, and 2,000 counts per second, respectively—on autofluorescence levels is explored (see Figure S13b). The results indicate that higher flow rates result in lower autofluorescence levels due to shorter acquisition times for cells. An approximate 15% difference is noted between low and high flow rates. For consistency, a medium flow rate with a count rate of ~1,000 cps is employed throughout this study.

In summary, these findings suggest that the inherent structural differences among cell lines exert a more significant influence on flow cytometry results than the flow rate of the measurements. The flow rate can be controlled well by the instrument, but the difference of autofluorescence levels among cell lines is intrinsic, cannot be changed, and should be considered.

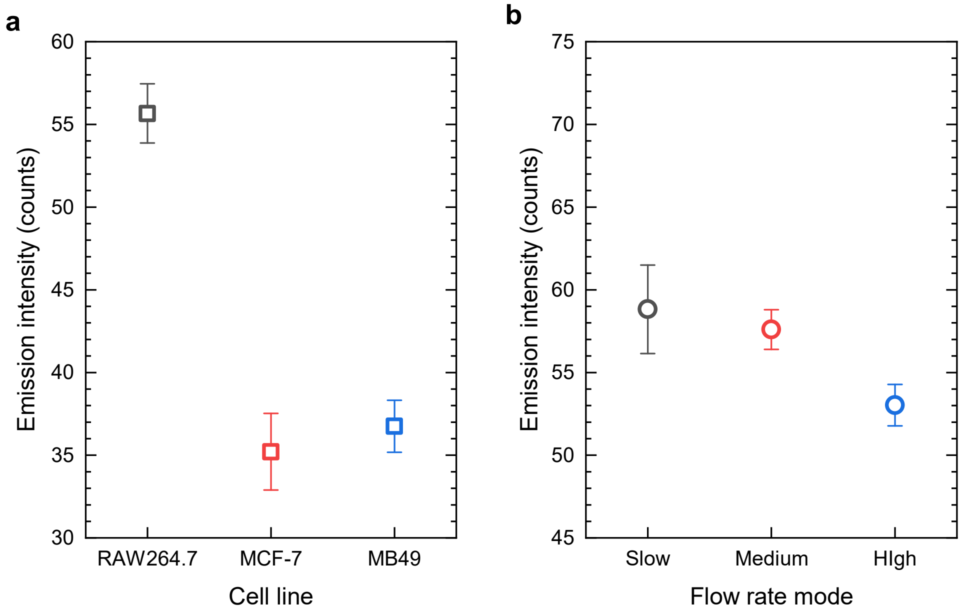

Figure S13. Autofluorescence signals under different conditions. (a) Autofluorescence level of RAW 264.7, MCF-7, and MB49 cell lines. (b) The autofluorescence level with respect to three different flow rates. Results are mean ± SD obtained from triplicate experiments.

Discussion S12. <u>*Cell cluster effect to the cellular fluorophore mass function*</u>

Measurements in the flow cytometry contain not only singlet but also clustered cells, if exist. All measured events have to be taken into account to correlate them with bulk measurement. That is, the data cannot be gated into singlet population. This will lead to overestimated autofluorescence background and (6,5) intensity per cell, if the number of cells is assumed to be the same as measurement events. In fact, this results in underestimated cellular (6,5) mass $\overline{m}$. The cellular fluorophore mass functions for all measured cells and gated singlet cells are

$$\begin{cases} \overline{m} = \gamma \times (\bar{I} - \bar{I}_0) \\ \overline{m} = \gamma_{\mathrm{g}} \times \left(\bar{I}_{\mathrm{g}} - \bar{I}_{0,\mathrm{g}}\right) \end{cases}$$

Assume that the non-gated vs gated ratio of average cell numbers per event is β. The following relation can be obtained

$$\begin{cases} \bar{I} = \beta \bar{I}_{\mathrm{g}} \\ \bar{I}_0 = \beta \bar{I}_{0,\mathrm{g}} \end{cases}$$

The cellular fluorophore mass function can be rewritten into

$$\begin{cases} \overline{m} = \gamma \times \beta\left(\bar{I}_{\mathrm{g}} - \bar{I}_{0,\mathrm{g}}\right) \\ \overline{m} = \gamma_{\mathrm{g}} \times \left(\bar{I}_{\mathrm{g}} - \bar{I}_{0,\mathrm{g}}\right) \end{cases}$$

Therefore

$$\gamma_{\mathrm{g}} = \beta\gamma$$

In summary, the correction procedure for singlet-gated cells, or for other type of gated cells is

1. Find β. The easiest method is to find the ratio of non-gated autofluorescence $\bar{I}_0$ to gated autofluorescence $\bar{I}_{0,\mathrm{g}}$; that is, $\beta = \bar{I}_0/\bar{I}_{0,\mathrm{g}}$
2. Find γ_{g}. The relation between γ_{g} and γ is $\gamma_{\mathrm{g}} = \beta\gamma$.

The resulting cellular fluorophore mass function is

$$\overline{m} = \beta\gamma \times \left(\bar{I}_{\mathrm{g}} - \bar{I}_{0,\mathrm{g}}\right) \tag{6}$$

For example, to deduce the cellular fluorophore mass function for singlet-cell gated population from all cells non-gated population, we should find the β first. Table S1 lists the autofluorescence for non-gated cells $\bar{I}_0$ and singlet-gated cells $\bar{I}_{0,\mathrm{g}}$ to be 81.1 and 54.0, respectively. The β therefore is $81.1/54.0 = 1.5$. The γ_{g} is $1.5 \times 83.9 = 126$.

Figure S14 compares the difference between corrected data from all cells and direct singlet-gated data. The emission intensity from direct gated data (in x-axis) does not contain the signals from cell clusters, while the cellular mass (in y-axis) deduced from bulk measurement includes the clustered ones. This leads to the different γ values between them. The β correction method is more appropriate and gives more accurate results.

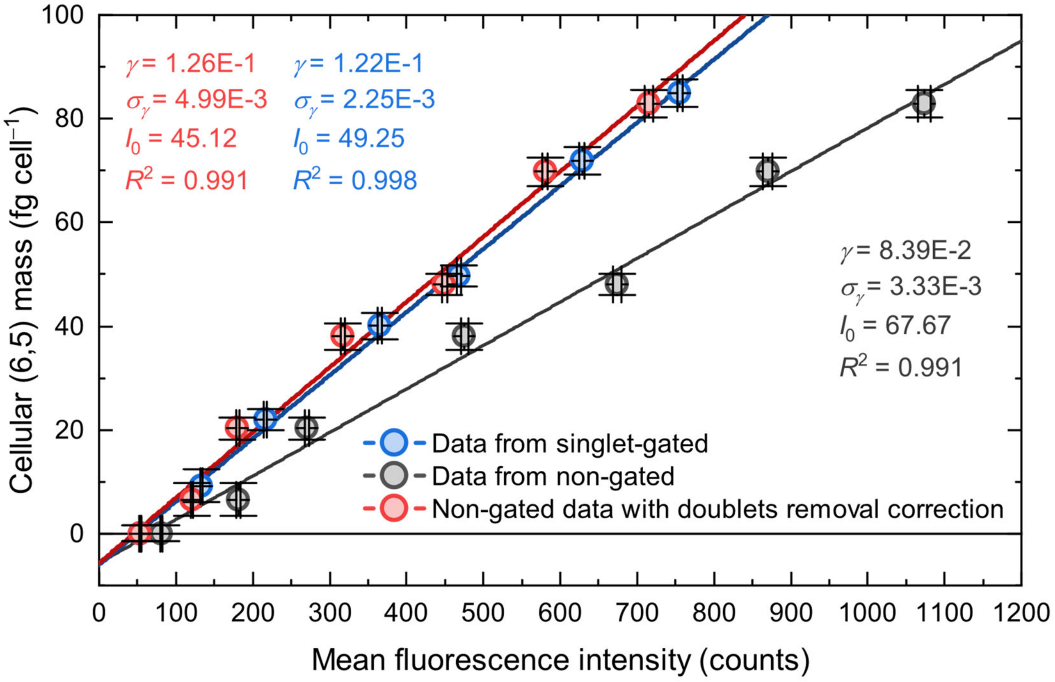

Figure S14. Cellular fluorophore mass functions from all measured cells (black), all measured cells corrected for singlet cells (red), and singlet-gated cells (blue). Linear lines are the fits to the data. Results are mean ± SD obtained from triplicate experiments.

Discussion S13. *Cell segmentation with ROI analysis in image cytometry*

Cell segmentation is a crucial step in successful image cytometry, and the freely available segmentation algorithm, Cellpose 2.0, is employed for selecting the appropriate cell regions for subsequent analysis. In one batch of measurements, the acquired brightfield and SWIR images in SPE format are saved and converted into tdms files as matrices, which are loaded into the Python program using the npTDMS package. These input matrices are exported into TIFF format, which can be loaded using the functions in the Cellpose package for conducting cell segmentation. The processing algorithm is summarized in Algorithm S1, involving several steps:

1. *Boundary Detection:*
   A list of cell boundaries is generated by employing **cellpose.io.save_masks** on a brightfield image (Figure S15a,h), with the output being a text file structured to contain coordinates outlining each cell boundary (Figure S15b). The input parameter “diameter” for Cellpose is consistently set at 30 in this work but can be modified for other cell lines.
2. *Mask Generation:*
   Using the boundary coordinates (Figure S15c), masks are generated using **skimage.draw.polygon2mask** to delineate the area enclosed by each cell boundary. Masks are excluded if the corresponding cell is cut off by the image edges or if the enclosed area is deemed too small.
3. *Composite Mask Creation:*
   Individual masks are overlaid onto a composite mask (Figure S15d), updating it to represent the collective areas covered by all cells.
4. *Mask Inversion:*
   The composite mask is inverted to highlight the regions covered by cells (Figure S15e).
5. *Fluorescence Image Overlay:*
   The fluorescence image (Figure S15f) is overlaid with the inverted composite mask, enabling the calculation of fluorescence intensity within each individual cell mask (Figure S15g).
6. *Intensity Calculation:*
   The sum of fluorescence intensity within each individual cell mask is calculated.

In step 3, it is essential to highlight that we performed connected component analysis and merged connected masks to create composite masks. Notably, our observations suggest that masks surpassing 1,500 pixels generally signify cell clusters, which are subsequently excluded in the gating mode.

As depicted in Figure S15h, one ROI is considered as one cell, successfully removing agglomerated cell clusters. It is essential to note that, for establishing the calibration line, the fluorescence

signal from the cell segmentation without gating is used to match the conditions of bulk measurements by the spectrometry method. The code for data processing has been provided on GitHub, and the website link is: https://github.com/jsw99/photonic-nanomaterials-lab/tree/main/Cell-Segmentation

Algorithm S1: Fluorescence intensity analysis for a single image.

Input: A list of cell boundaries, *L*, where each sub-list, *S*, corresponds to one cell and contains tuples representing the coordinates of the cell boundary.

Output: Fluorescence intensity of single-walled carbon nanotubes incubated within each cell.

```
for each S in L do
    if a cell is cut off by edges of an image then
        Exclude this cell.
    else
        Include this cell.
        Generate a mask of the area enclosed by the boundary.
        if the enclosed area is too small then
            Exclude this individual mask.
        else
            Include this individual mask.
            Overlay this individual mask onto composite mask.
            Update composite mask.
        end
    end
end
Invert the composite mask.
Overlay the composite mask onto the fluorescence image.
Calculate the sum of the fluorescence intensity within each individual mask.
```

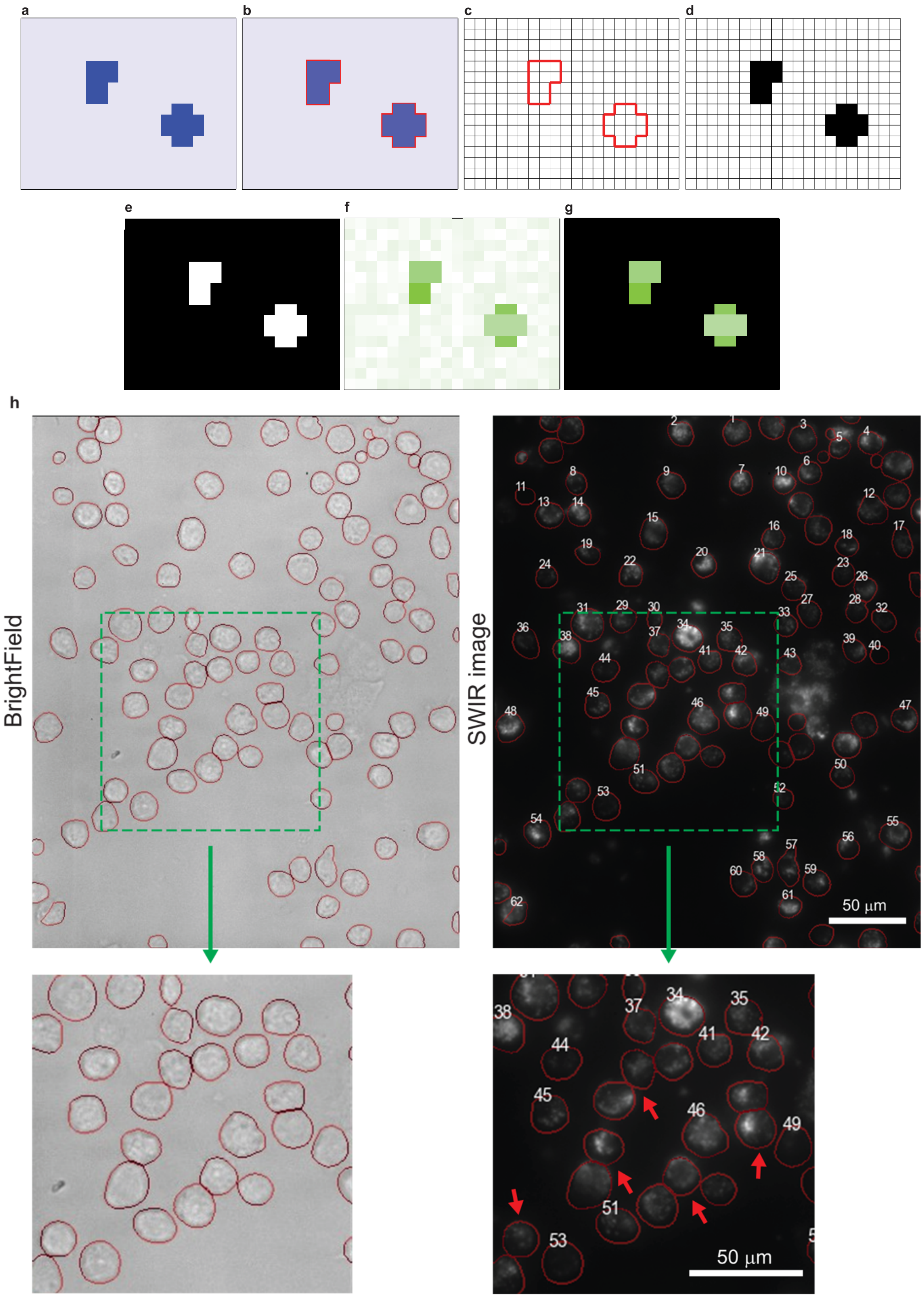

Figure S15. Workflow for fluorescence intensity analysis. Example images (a-g) are represented by 40×32 grids, where real images (h) by 640×512 pixels. (a) Brightfield image, where blue areas represent cells. (b) Cells with outlines. (c) Pure outlines and (d) Masked areas enclosed by the outlines. (e) Inverted masks. (f) Fluorescence image, where fluorescence signals are depicted by darker green, and the lower intensity background is shown in white to light green. (g) Overlapping of inverted masks with fluorescence image. (h) SWIR and brightfield images of (6,5)-treated cells. Bright pixels in the SWIR image represent the SWCNT signals. Red margins represent the selected ROI regions. Red arrows represent clustered cells with edge contact, which will not be included in the analyses, while number-labelled ROIs were included in the analyses.

Discussion S14. *<u>Intracellular (6,5) quantification using image cytometry</u>*

After integrating all cell signals in the selected ROIs in the acquired images, the data is converted into the probability histogram of ROI's integrated intensity, as shown in Figure S16a. The MFIs of samples treated with different (6,5) concentrations are further calculated and plotted in Figure S16b. Eq 1, with information including the calibration slope ρ_{spec} and specific cell number n_{cell}, converts MFIs into cellular (6,5) mass, giving a plot of cellular (6,5) mass versus (6,5) dose, as shown in Figure S16c. This plot conveys the same information as Figure 2c in the main text, despite being deduced from two different methods. The fitted parameter values are very similar, and the small difference originates from measurement errors. Figure S16d shows the comparison of SNRs between the image and flow cytometers. The SNR is defined as the ratio of MFI of (6,5)-treated cells (300 μg L^{-1}) to the standard error of the mean of the autofluorescence signal from unstained cells. The SNR for the image cytometry is ~10 times higher than that for the flow cytometry, likely due to the larger numerical aperture and lower detector noise of the image cytometer.

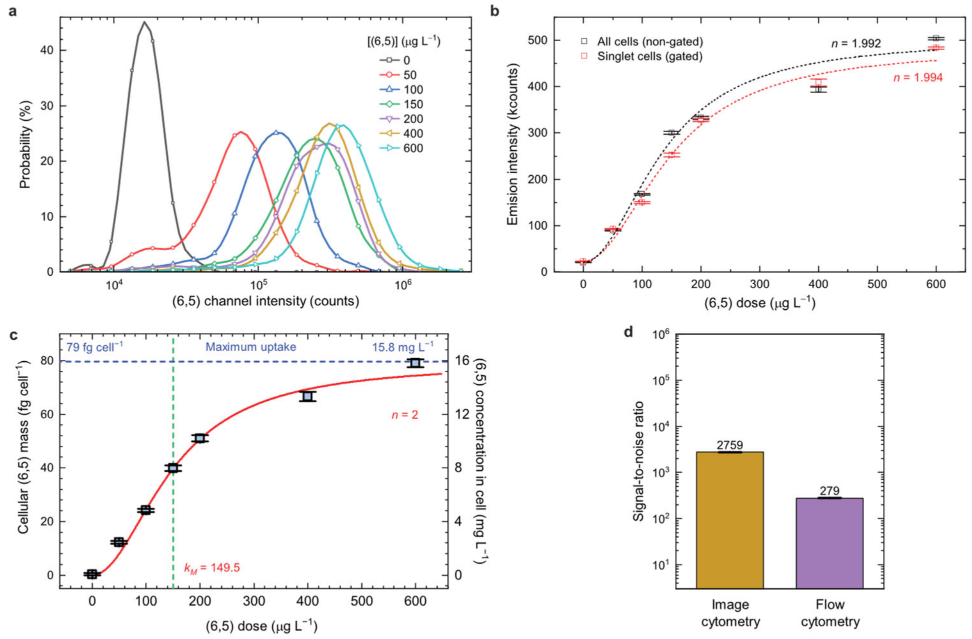

Figure S16. Quantification of (6,5) in RAW 264.7 using the SWIR image cytometer. (a) Probability histograms, which are binned at a width of log(I) = 1.3, of integrated (6,5) emission intensities from all measured cells (non-gated). (b) MFI of (6,5) with respect to the treated (6,5) concentration. The data is fitted by the Hill function, as described above. (c) Cellular (6,5) mass with respect to the treated (6,5) concentration. The *n* value is the Hill coefficient and the k_M value is the Michaelis constant. (d) SNRs of the (6,5) channels for the image and flow cytometers.

Table S4. Mean, median, FWHM, SD and SE of sample fluorescence intensity from the image cytometer.

Dose ($\mu g\ L^{-1}$)	Mean	Median	FWHM	SD	SE
Total cell population (non-gated)					
0	20,370	19,300	5,180	6,290	110
50	89,200	81,600	45,900	56,000	1,100
100	167,500	149,800	88,400	92,900	1,400
150	300,400	272,100	221,600	163,600	3,000
200	332,700	307,000	307,500	180,900	3,800
400	393,700	349,900	307,500	244,900	5,800
600	503,700	448,700	236,200	293,600	2,800
Singlet cell population (gated)					
0	22,770	21,750	6,570	5,620	150
50	92,600	83,800	52,600	54,500	1,200
100	162,200	145,700	118,300	79,500	1,800
150	254,100	223,400	203,800	141,800	3,800
200	319,300	288,600	315,500	157,100	3,500
400	411,000	364,700	361,500	236,900	7,800
600	483,500	426,800	315,500	270,200	2,500

Discussion S15. *Calculation of the limits of detection of the SWIR cytometers*

The total signal intensity I_s equals the cell autofluorescence intensity I_0 plus the (6,5) intensity $I_{(6,5)}$

$$I_s = I_0 + I_{(6,5)}$$

The I_s does not contain detector noise signal. Note that the cell autofluorescence I_0 and the (6,5) intensity $I_{(6,5)}$ both have their own distributions,

$$\begin{cases} N_0 = N_0(I_0) \\ N_{(6,5)} = N_{(6,5)}\left(I_{(6,5)}\right) \end{cases}$$

We can calculate the sampled average intensities from cell autofluorescence and (6,5) emission, $\bar{I}_0$ and $\bar{I}_{(6,5)}$, respectively. The total average intensity is then

$$\bar{I}_s = \bar{I}_0 + \bar{I}_{(6,5)}$$

Assume that the average cell autofluorescence and (6,5) intensities of the whole population are $\bar{I}_0^{\infty}$ and $\bar{I}_{(6,5)}^{\infty}$, respectively. The total intensity of the whole population is then

$$\bar{I}_s^{\infty} = \bar{I}_0^{\infty} + \bar{I}_{(6,5)}^{\infty}$$

The relations between sample and population intensities of the cell autofluorescence and (6,5) emission are

$$\begin{cases} \bar{I}_0 = \bar{I}_0^{\infty} \pm \sigma_{\bar{I}_0} \\ \bar{I}_{(6,5)} = \bar{I}_{(6,5)}^{\infty} \pm \sigma_{\bar{I}_{(6,5)}} \\ \bar{I}_s = \bar{I}_s^{\infty} \pm \sigma_{\bar{I}_s} \end{cases}$$

where the $\sigma_{\bar{I}_0}$ and $\sigma_{\bar{I}_{(6,5)}}$ are the standard error of the means of the cell autofluorescence and (6,5) emission intensities. They can also be written as

$$\begin{cases} \sigma_{\bar{I}_0} = \dfrac{\sigma_{I_0}}{\sqrt{N}} \\ \sigma_{\bar{I}_{(6,5)}} = \dfrac{\sigma_{I_{(6,5)}}}{\sqrt{N}} \end{cases}$$

where σ_{I_0} and $\sigma_{I_{(6,5)}}$ are the standard deviations of the intensity distributions of cell autofluorescence and (6,5) emission, respectively, and N is the number of the sampling of the cells

$$\bar{I}_s = \bar{I}_0 + \bar{I}_{(6,5)} = \left(\bar{I}_0^{\infty} \pm \sigma_{\bar{I}_0}\right) + \left(\bar{I}_{(6,5)}^{\infty} \pm \sigma_{\bar{I}_{(6,5)}}\right)$$

$$\bar{I}_s = \bar{I}_s^{\infty} \pm \sigma_{\bar{I}_s} = \left(\bar{I}_0^{\infty} + \bar{I}_{(6,5)}^{\infty}\right) \pm \sqrt{{\sigma_{\bar{I}_0}}^2 + {\sigma_{\bar{I}_{(6,5)}}}^2}$$

Now, we want to deduce the average (6,5) emission intensity from the whole population,

$$\bar{I}^{\infty}_{(6,5)} = \bar{I}^{\infty}_{s} - \bar{I}^{\infty}_{0}$$

$$\bar{I}^{\infty}_{(6,5)} = \left(\bar{I}_s \pm \sigma_{\bar{I}_s}\right) - \left(\bar{I}_0 \pm \sigma_{\bar{I}_0}\right)$$

$$\bar{I}^{\infty}_{(6,5)} = (\bar{I}_s - \bar{I}_0) \pm \sqrt{\sigma_{\bar{I}_s}{}^2 + \sigma_{\bar{I}_0}{}^2}$$

$$\bar{I}^{\infty}_{(6,5)} = (\bar{I}_s - \bar{I}_0) \pm \sqrt{\frac{\sigma_{I_s}{}^2 + \sigma_{I_0}{}^2}{N}}$$

We can deduce the average mass of the (6,5) in the cells $\bar{m}^{\infty}$ using bulk spectrometry measurements.

$$\bar{m}^{\infty} = \bar{m} \pm \sigma_{\bar{m}}$$

We assume that the mass of the (6,5) inside the cells is proportional to the detected (6,5) emission intensities, as depicted in Eq 2 of the main text

$$\bar{m}^{\infty} = \bar{m}^{\infty}\left(\bar{I}^{\infty}_{(6,5)}\right) = \gamma \times \bar{I}^{\infty}_{(6,5)}$$

$$\gamma \pm \sigma_{\gamma} = \frac{\bar{m} \pm \sigma_{\bar{m}}}{(\bar{I}_s - \bar{I}_0) \pm \sqrt{\frac{\sigma_{I_s}{}^2 + \sigma_{I_0}{}^2}{N}}} \qquad (7)$$

where γ is the slope and σ_{γ} is the standard deviation of the function. Here, we define the limit of detection (LOD) by applying 3σ rule, that is, the average population (6,5) intensity $\bar{I}^{\infty}_{(6,5)}$ to the standard deviation of the mean signal from the non-stained cell sample $\sigma_{\bar{0}}$ should be greater than three. We can write the equation into

$$\frac{S}{N} = \frac{\bar{I}^{\infty}_{(6,5)}}{\sigma_{\bar{0}}} > 3 \qquad (8)$$

Note that the standard deviation $\sigma_{\bar{0}}$ contains several factors, including instrument noise $\sigma_{\overline{\text{inst}}}$, shot noise $\sigma_{\overline{\text{shot}}}$, and sampling error of the autofluorescence $\sigma_{\bar{I}_0}$. Therefore, the $\sigma_{\bar{0}}$ can be expressed as

$$\sigma_{\bar{0}} = \sqrt{\sigma^2_{\overline{\text{inst}}} + \sigma^2_{\overline{\text{shot}}} + \sigma^2_{\bar{I}_0}}$$

where

$$\begin{cases} \sigma_{\overline{\text{inst}}} = \dfrac{\sigma_{\text{inst}}}{\sqrt{N}} \\ \sigma_{\overline{\text{shot}}} = \dfrac{\sigma_{\text{shot}}}{\sqrt{N}} \end{cases}$$

Rearranging the SNR equation gives

$$\bar{I}_{(6,5)}^{\infty} > 3 \times \sigma_{\bar{0}}$$

The $\sigma_{\bar{0}}$ can be expressed as

$$\sigma_{\bar{0}} = \frac{\sigma_0}{\sqrt{N}}$$

Therefore, the equation becomes

$$\bar{I}_{(6,5)}^{\infty} > \frac{3 \times \sigma_0}{\sqrt{N}}$$

In addition, the population mean of the (6,5) intensity $\bar{I}_{(6,5)}^{\infty}$ can be re-written into the sample mean of the (6,5) intensity $\bar{I}_{(6,5)}$ with a sampling error

$$\bar{I}_{(6,5)} \pm \sqrt{\frac{{\sigma_{I_s}}^2 + {\sigma_{I_0}}^2}{N}} > \frac{3 \times \sigma_0}{\sqrt{N}}$$

The LOD of the sample mean of the (6,5) intensity $\bar{I}_{(6,5)}^{\text{LOD}}$ is the minimum value of $\bar{I}_{(6,5)}$

$$\bar{I}_{(6,5)}^{\text{LOD}}(N) \pm \sqrt{\frac{{\sigma_{I_s}}^2 + {\sigma_{I_0}}^2}{N}} = \frac{3\sigma_0}{\sqrt{N}}$$

$$\bar{I}_{(6,5)}^{\text{LOD}}(N) = \frac{3\sigma_0}{\sqrt{N}} \pm \sqrt{\frac{{\sigma_{I_s}}^2 + {\sigma_{I_0}}^2}{N}}$$

Therefore, the LOD of (6,5) mass in cell is

$$\bar{m}^{\text{LOD}}\left(\bar{I}_{(6,5)}^{\text{LOD}}\right) = \gamma \times \bar{I}_{(6,5)}^{\text{LOD}} = [\gamma \pm \sigma_\gamma] \times \left[\frac{3\sigma_0}{\sqrt{N}} \pm \sqrt{\frac{{\sigma_{I_s}}^2 + {\sigma_{I_0}}^2}{N}}\right]$$

The equation can be rearranged into

$$\bar{m}^{\mathrm{LOD}}(N) \pm \sigma_{\bar{m}^{\mathrm{LOD}}}(N) = \frac{3\gamma\sigma_0}{\sqrt{N}} \pm \bar{m}^{\mathrm{LOD}} \times \sqrt{\left(\frac{\sigma_\gamma}{\gamma}\right)^2 + \left(\frac{\sqrt{\frac{{\sigma_{I_\mathrm{s}}}^2 + {\sigma_{I_0}}^2}{N}}}{\frac{3\sigma_{I_0}}{\sqrt{N}}}\right)^2}$$

$$\bar{m}^{\mathrm{LOD}}(N) \pm \sigma_{\bar{m}^{\mathrm{LOD}}}(N) = \frac{3\gamma\sigma_0}{\sqrt{N}} \pm \frac{3\gamma\sigma_0}{\sqrt{N}} \sqrt{\left(\frac{\sigma_\gamma}{\gamma}\right)^2 + \frac{1}{9}\left[\left(\frac{\sigma_{I_\mathrm{s}}}{\sigma_{I_0}}\right)^2 + 1\right]} \tag{9}$$

Note that γ and σ_γ are constant values for a specific modality including a defined fluorophore and a defined cytometry system. σ_{I_0} is the standard deviation of the autofluorescence distribution and is a constant value for the same cell line measured with the same cytometry system. The σ_{I_s} changes with changing $\bar{I}_{(6,5)}$.

Figure S17a illustrates the total standard error of the mean σ_{total} with respect to the total intensity of the whole population $\bar{I}_\mathrm{s}$. The measured σ_{total} includes not only σ_{I_s} but also other distributions, including instrument noise σ_{inst} and shot noise σ_{shot}. Therefore,

$$\sigma_{\mathrm{total}} = \sqrt{\sigma_{\mathrm{inst}}^2 + \sigma_{\mathrm{shot}}^2 + \sigma_{I_\mathrm{s}}^2} = \sqrt{\sigma_{\mathrm{inst}}^2 + \bar{I}_\mathrm{s} + \sigma_{I_\mathrm{s}}^2} \tag{10}$$

A special case of this is the cell without (6,5)

$$\sigma_0 = \sqrt{\sigma_{\mathrm{inst}}^2 + \bar{I}_0 + \sigma_{I_0}^2} \tag{11}$$

Taking the ratio of these two equations gives

$$\frac{\sigma_{I_\mathrm{s}}}{\sigma_{I_0}} = \sqrt{\frac{\sigma_{\mathrm{total}}^2 - \sigma_{\mathrm{inst}}^2 - \bar{I}_\mathrm{s}}{\sigma_0^2 - \sigma_{\mathrm{inst}}^2 - \bar{I}_0}}$$

The empirical formula obtained from the fitted curve in Figure S17a is used

$$\sigma_{\mathrm{total}} = \xi\bar{I}_\mathrm{s} + \sigma_{\mathrm{inst}} \tag{12}$$

Replacing the σ_{total} using this equation gives

$$\frac{\sigma_{I_\mathrm{s}}}{\sigma_{I_0}} = \sqrt{\frac{(\xi\bar{I}_\mathrm{s} + \sigma_{\mathrm{inst}})^2 - \sigma_{\mathrm{inst}}^2 - \bar{I}_\mathrm{s}}{\sigma_0^2 - \sigma_{\mathrm{inst}}^2 - \bar{I}_0}}$$

Rearrange the equation gives

$$\frac{\sigma_{I_s}}{\sigma_{I_0}} = \sqrt{\frac{(\xi^2 \bar{I}_s + 2\xi\sigma_{\text{inst}} - 1)\bar{I}_s}{\sigma_0^2 - \sigma_{\text{inst}}^2 - \bar{I}_0}}$$

We know that

$$\bar{I}_s = \bar{I}_{(6,5)} + \bar{I}_0$$

Therefore,

$$\frac{\sigma_{I_s}}{\sigma_{I_0}} = \sqrt{\frac{\left[\xi^2\left(\bar{I}_{(6,5)} + \bar{I}_0\right) + 2\xi\sigma_{\text{inst}} - 1\right]\left(\bar{I}_{(6,5)} + \bar{I}_0\right)}{\sigma_0^2 - \sigma_{\text{inst}}^2 - \bar{I}_0}}$$

In addition,

$$\bar{I}_{(6,5)} = \frac{3\sigma_0}{\sqrt{N}}$$

So

$$\frac{\sigma_{I_s}}{\sigma_{I_0}} = \sqrt{\frac{\left[\xi^2\left(\frac{3\sigma_0}{\sqrt{N}} + \bar{I}_0\right) + 2\xi\sigma_{\text{inst}} - 1\right]\left(\frac{3\sigma_0}{\sqrt{N}} + \bar{I}_0\right)}{\sigma_0^2 - \sigma_{\text{inst}}^2 - \bar{I}_0}}$$

Therefore, the LOD of the (6,5) mass is

$$\bar{m}^{\text{LOD}}(N) \pm \sigma_{\bar{m}^{\text{LOD}}}(N) = \frac{3\gamma\sigma_0}{\sqrt{N}} \pm \frac{3\gamma\sigma_0}{\sqrt{N}}\sqrt{\left(\frac{\sigma_\gamma}{\gamma}\right)^2 + \frac{1}{9}\left[\frac{\left[\xi^2\left(\frac{3\sigma_0}{\sqrt{N}}+\bar{I}_0\right)+2\xi\sigma_{\text{inst}}-1\right]\left(\frac{3\sigma_0}{\sqrt{N}}+\bar{I}_0\right)}{\sigma_0^2-\sigma_{\text{inst}}^2-\bar{I}_0} + 1\right]} \qquad (13)$$

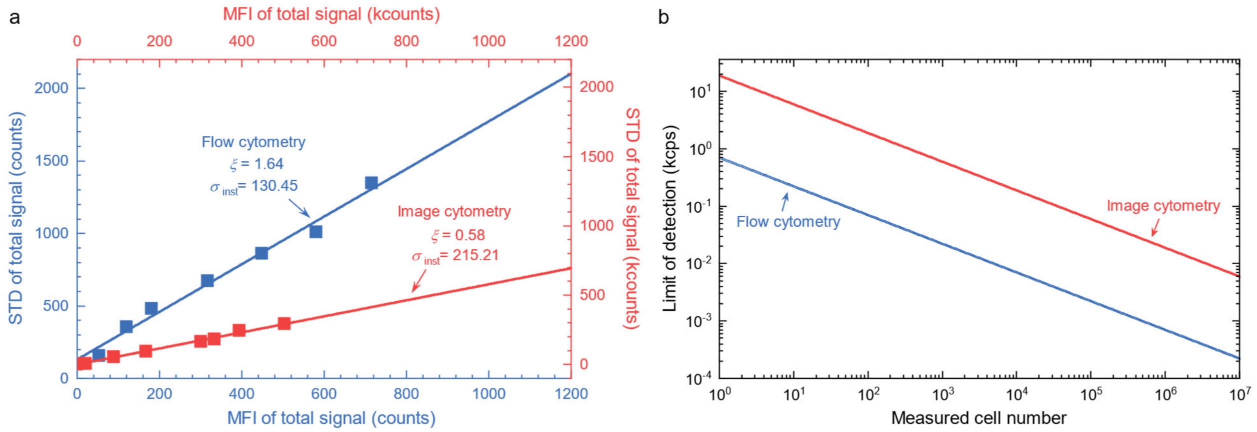

Figure S17. Deduction of parameter values required for LOD estimation. (a) σ_{total} with respect to $\bar{I}_s$ of samples measured by flow and image cytometers. ξ and σ_{inst} are the slope and the intercept of the fitted lines, respectively. (b) LOD in the unit of cps with respect to measured cell number.

The parameters σ_0 and $\bar{I}_0$ are included in the original measurement data. The γ is deduced from the calibration line. The ξ and σ_{inst} is estimated from the slope and intercept of the fitted line, respectively, for the cytometers in Figure S17a. The intercept for the image cytometer can be estimated from the camera noise and is fixed at the estimated value.

Table S5. LOD of (6,5) in units of cps, fg cell^{-1}, and tubes cell^{-1} for the flow and image cytometers with various measured cell numbers.

Measured number	LOD in cps		LOD in fg cell^{-1}		LOD in tubes cell^{-1}*	
	Flow	Image	Flow	Image	Flow	Image
10^1	222±74	5,960±20	19±32	1.02±0.90	53,000±94,000	3,000±2,600
10^2	70±24	1,885±63	6±6	0.3±0.2	16,700±18,400	920±710
10^3	22±8	600±200	2±2	0.10±0.07	5,300±4,700	300±230
10^4	7±3	189±63	0.6±0.5	0.03±0.02	1,700±1,400	92±67
10^5	2.2±0.7	60±20	0.2±0.2	0.01±0.007	530±420	29±21
10^6	0.7±0.2	19±6	0.06±0.05	0.003±0.002	170±130	9±7
10^7	0.2±0.1	6±2	0.02±0.02	0.001±0.0007	53±41	3±2

*The number of tubes per cell is determined by dividing cellular (6,5) mass divided by the weight of an individual 200-nm (6,5), which is ~3.52×10^{-4} fg. The tube length is estimated based on the commonly used sample preparation protocol, and the weight per unit length is calculated based on the assumption of a C-C bond distance at 0.144 nm.

Discussion S16. Comparison of the LODs of the two cytometric methods

Figure 3a in the main text illustrates the $\bar{m}^{\mathrm{LOD}}(N)$ from the flow and image cytometers. Here, we compare their performances through their LODs at the same measured cell number and their required measured cell numbers at the same LODs. We know the formula for determining the LOD of the (6,5) mass from the previous section, and the formula is written here again:

$$\bar{m}^{\mathrm{LOD}}(N) = \frac{3\gamma\sigma_0}{\sqrt{N}}$$

1) In the first case, assume that two methods have the same LOD, that is,

$$\bar{m}_{\mathrm{I}}^{\mathrm{LOD}} = \bar{m}_{\mathrm{F}}^{\mathrm{LOD}}$$

where $\bar{m}_{\mathrm{I}}^{\mathrm{LOD}}$ and $\bar{m}_{\mathrm{F}}^{\mathrm{LOD}}$ are the LODs of the image and flow cytometers. Combining the two equations gives

$$\frac{3\gamma_{\mathrm{I}}\sigma_{0,\mathrm{I}}}{\sqrt{N_{\mathrm{I}}}} = \frac{3\gamma_{\mathrm{F}}\sigma_{0,\mathrm{F}}}{\sqrt{N_{\mathrm{F}}}}$$

Or

$$\boxed{\frac{N_{\mathrm{F}}}{N_{\mathrm{I}}} = \left(\frac{\gamma_{\mathrm{F}}\sigma_{0,\mathrm{F}}}{\gamma_{\mathrm{I}}\sigma_{0,\mathrm{I}}}\right)^2} \tag{14}$$

In our experimental results,

$$\gamma_{\mathrm{F}} = 1.26 \times 10^{-10};\ \sigma_{0,\mathrm{F}} = 155.76;\ \gamma_{\mathrm{I}} = 1.71 \times 10^{-13};\ \sigma_{0,\mathrm{I}} = 6{,}284.41$$

The ratio of the measured cell number between flow and image cytometers is

$$\frac{N_{\mathrm{F}}}{N_{\mathrm{I}}} = 333.5$$

2) In the second case, assume that two methods have measured the same cell number. Then,

$$N_{\mathrm{I}} = N_{\mathrm{F}}$$

$$\left(\frac{3\gamma_{\mathrm{I}}\sigma_{0,\mathrm{I}}}{\bar{m}_{\mathrm{I}}}\right)^2 = \left(\frac{3\gamma_{\mathrm{F}}\sigma_{0,\mathrm{F}}}{\bar{m}_{\mathrm{F}}}\right)^2$$

$$\boxed{\frac{\bar{m}_{\mathrm{F}}^{\mathrm{LOD}}}{\bar{m}_{\mathrm{I}}^{\mathrm{LOD}}} = \frac{\gamma_{\mathrm{F}}\sigma_{0,\mathrm{F}}}{\gamma_{\mathrm{I}}\sigma_{0,\mathrm{I}}}} \tag{15}$$

In our experimental results, the parameter values are the same as that in the first case. The ratio of the LODs of the flow and image cytometers is

$$\frac{\bar{m}_{\mathrm{F}}^{\mathrm{LOD}}}{\bar{m}_{\mathrm{I}}^{\mathrm{LOD}}} = 18.3$$

Comparing the results between two cases, we can obtain the following relation

$$\boxed{\frac{N_{\mathrm{F}}}{N_{\mathrm{I}}} = \left(\frac{\bar{m}_{\mathrm{F}}^{\mathrm{LOD}}}{\bar{m}_{\mathrm{I}}^{\mathrm{LOD}}}\right)^2} \quad (16)$$

The ratio of the cell numbers required to achieve the same LOD equals the square of the LOD ratio when the same number of measurements are acquired. Specifically, if one system has a LOD η times lower than the other, it is necessary to acquire η^2 times more measurements to attain the same LOD. In our scenario, the flow cytometer exhibits a LOD 18.3 times lower than the image cytometry. Consequently, the number of cells required for the flow cytometer is $18.3^2 = 334.5$ times greater than that for the image cytometry.

Discussion S17. *Estimation of the system performance*

Here, we estimate the time required to achieve the same data quality for the two cytometers. Table S6 provides details on the time required for each step. The "Number of cells" represents the cells acquired from the cytometers for data analysis. The "Pre-acquisition preparation time" includes actions such as cell resuspension for flow cytometry and cell fixation, wash, addition to cover-slide chambers, and finding image focus and sample positions for image cytometry. "Acquisition time" is the total time needed for the instruments to capture data. "Data processing time" is the time required to convert raw data into the probability histogram. "Total time" is the sum of pre-acquisition preparation time, acquisition time, and data processing time. The "Rate" is the number of cells divided by the total time, representing how many cells the cytometry system can measure per unit time. It is important to note that the pre-acquisition preparation time and data processing time for image cytometry can be shortened with a more automated system, improved segmentation algorithms, and more powerful computers. In contrast, acquisition time is limited by system performance, which is relatively challenging to reduce unless more sensitive detectors, more powerful excitation, or better optical designs are available. However, increasing the excitation power in our image cytometer is feasible and can easily improve the SNR by a factor of several.

The ratio of the rate between two methods $r_\mathrm{F}/r_\mathrm{I}$ is

$$\frac{r_\mathrm{F}}{r_\mathrm{I}} = \frac{15{,}213.2}{25.02} = 608$$

The total time required for an experiment is

$$t = N/r$$

Therefore, the ratio of time required between two methods is

$$\frac{t_\mathrm{F}}{t_\mathrm{I}} = \frac{N_\mathrm{F} r_\mathrm{I}}{N_\mathrm{I} r_\mathrm{F}} = \frac{N_\mathrm{F}/N_\mathrm{I}}{r_\mathrm{F}/r_\mathrm{I}} = \frac{333.5}{608} = 0.55 = \frac{1}{1.82}$$

Table S6. Consuming time of acquiring and processing data from flow or image cytometry.

	Number of cells	Pre-acquisition preparation time (min)	Acquisition time (min)	Data processing time (min)	Total time (min)	Rate (cell min^{-1})
Flow cytometry	365,116[#]	3	15.1	6	24	15,213.2
Image cytometry	17,965	90	214	414	728	25.02

[#]Event in flow cytometry was considered as cell.

Discussion S18. <u>Fluorescence spectra of fluorophores used in this work</u>

Carbon nanotubes do not have the same molecular weight due to variations in length. To facilitate a fair comparison with other fluorophores including Hoechst, DCF and PI in Figure 3a and Figure S18, we assume a 50-carbon (6,5) nanotube with a molecular weight of ~600, aligning it with the molecular weights of those fluorophores. The power calibrated fluorescence spectra are subsequently divided by the molar concentration of the fluorophore and then multiplied by the spectral quantum efficiency of the APD detector. The SWCNTs, including (6,5), are known to have no emission in the visible range, as confirmed by the emission spectra in Figure S18a. Therefore, the SWCNTs' emission will not contribute to the spillover signals, which will be discussed in later sections.

Figure S18b displays the SWIR fluorescence spectra in a logarithmic scale, instead of the linear scale shown in Figure 3a. PI exhibits the highest emission intensity across the entire SWIR range. Other fluorophores, including DCF, CRDR, and Hoechst 33342, show approximately one order of magnitude lower emission intensity compared to (6,5). This implies that the signal contribution from these dyes in the SWIR channel during cytometry measurements is negligible, unless the (6,5) concentration is ten times lower than the others.

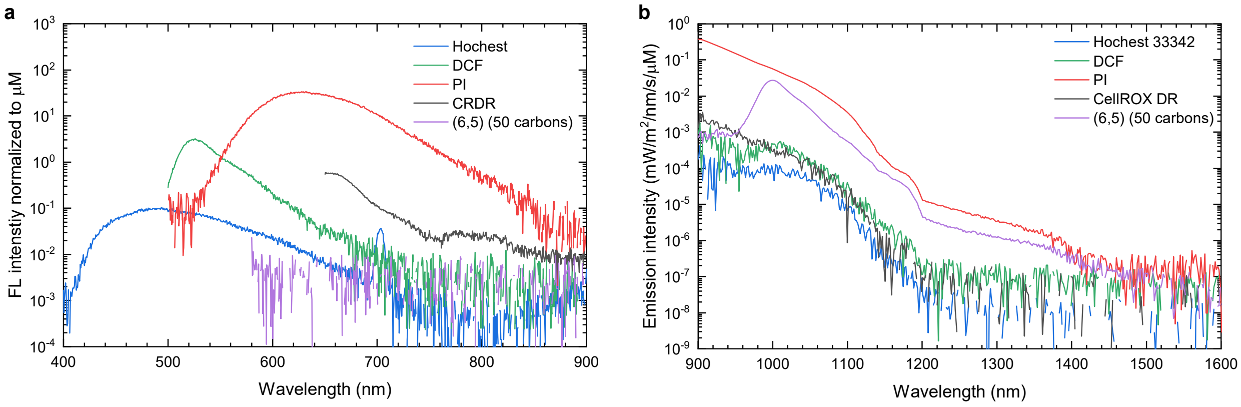

Figure S18 | log-linear plots of fluorophore spectra of fluorophores in the visible and SWIR windows. (a, b) Fluorescence spectra of Hoechst, DCF, PI, CRDR, and (6,5) in the (a) visible range and (b) SWIR range.

Discussion S19. <u>Spillover compensation</u>

Spillover compensation is a crucial step in flow cytometry analysis, particularly in a multi-fluorophore system. Figure S19 illustrates a scenario with two channels (CH1 and CH2) detecting emissions from two fluorophores (FL1 and FL2). A portion of the emission from FL1 spills into CH2, leading to potential overestimation of FL2 fluorescence intensity, and vice versa.

Assuming the observed signal O is a combination of signals from all fluorophores and cell autofluorescence, the relationship can be expressed as:

$$\begin{cases} O_1 = R_{11} + R_{21} + A_1 \\ O_2 = R_{22} + R_{12} + A_2 \end{cases} \tag{17}$$

where O_1 is the signal observed by CH1, R_{11} is the FL1 signal collected by CH1, R_{21} is the is the FL2 signal spilling into and collected by CH2, and A_1 is the autofluorescence signal collected by CH1. Similar notation applies to the second equation.

Obtain spillover coefficients. Assume that the spectral shape of the fluorophores remains unchanged during the experiments. Single-stained cell samples and a non-stained cell sample are required as controls. The spillover coefficient S_{ij} is defined as the ratio of spillover channel signal to target channel signal:

$$S_{ij} = \frac{R_{ij}}{R_{ii}} = \frac{O_{ij}^{s} - A_j}{O_{ii}^{s} - A_i} \tag{18}$$

where i is the target fluorophore/channel and j represents the spillover channel. A dual-stained system has two spillover coefficients:

$$\begin{cases} S_{12} = \frac{R_{12}}{R_{11}} = \frac{O_{12}^{s} - A_2}{O_{11}^{s} - A_1} \\ S_{21} = \frac{R_{21}}{R_{22}} = \frac{O_{21}^{s} - A_1}{O_{22}^{s} - A_2} \end{cases} \tag{19}$$

where O_{12}^{s} is the observed signal collected in CH2 using PL1-stained cells and A_2 is the observed autofluorescence signal collected in CH2 using non-stained cells. The definitions are the same for the second equation.

Combining two sets of equations results in:

$$\begin{cases} O_1 = R_{11} + S_{21}R_{22} + A_1 \\ O_2 = R_{22} + S_{12}R_{11} + A_2 \end{cases} \tag{20}$$

Rewrite these equations into matrix representation:

$$\begin{bmatrix} O_1 \\ O_2 \end{bmatrix} = \begin{bmatrix} 1 & S_{21} \\ S_{12} & 1 \end{bmatrix} \begin{bmatrix} R_{11} \\ R_{22} \end{bmatrix} + \begin{bmatrix} A_1 \\ A_2 \end{bmatrix} \tag{21}$$

Therefore,

$$\mathcal{O} = \mathcal{S}\mathcal{R} + \mathcal{A} \tag{22}$$

Rearranging the equation gives

$$\mathcal{R} = \mathcal{S}^{-1}(\mathcal{O} - \mathcal{A}) \tag{23}$$

where

$$\mathcal{R} = \begin{bmatrix} R_{11} \\ R_{22} \end{bmatrix}; \mathcal{S} = \begin{bmatrix} 1 & S_{21} \\ S_{12} & 1 \end{bmatrix}; \mathcal{O} = \begin{bmatrix} O_1 \\ O_2 \end{bmatrix}; \mathcal{A} = \begin{bmatrix} A_1 \\ A_2 \end{bmatrix} \tag{24}$$

A more generalized form for an n-fluorophore, n-channel system is:

$$\mathcal{R} = \begin{bmatrix} R_{11} \\ \vdots \\ R_{nn} \end{bmatrix}; \mathcal{S} = \begin{bmatrix} 1 & \cdots & S_{n1} \\ \vdots & \ddots & \vdots \\ S_{1n} & \cdots & 1 \end{bmatrix}; \mathcal{O} = \begin{bmatrix} O_1 \\ \vdots \\ O_n \end{bmatrix}; \mathcal{A} = \begin{bmatrix} A_1 \\ \vdots \\ A_n \end{bmatrix} \tag{25}$$

The spillover matrix $\mathcal{S}$ is obtained from single-stained and non-stained cell samples (control group), while the autofluorescence signal $\mathcal{A}$ is obtained from a non-stained cell sample (control group). The observed signal $\mathcal{O}$ is acquired from all-stained cells (experimental group). Using this information, the real signals $\mathcal{R}$ from all fluorophores in their corresponding channels can be deduced.

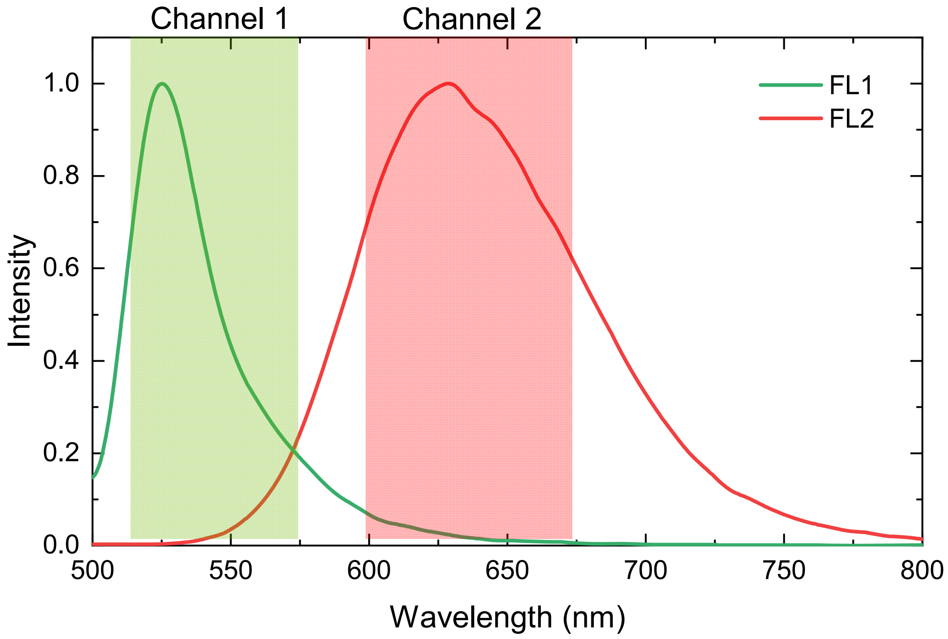

Figure S19. An example of fluorescence spectra and channel windows of a two-fluorophore system.

Discussion S20. <u>*Calculation of spillovers between dyes and (6,5)*</u>

The spillover coefficients are calculated as follows

$$\begin{cases} S_{(6,5),\mathrm{dye}} = \dfrac{R_{(6,5),\mathrm{dye}}}{R_{(6,5),(6,5)}} = \dfrac{O^s_{(6,5),\mathrm{dye}} - A_{\mathrm{dye}}}{O^s_{(6,5),(6,5)} - A_{(6,5)}} \\ S_{\mathrm{dye},(6,5)} = \dfrac{R_{\mathrm{dye},(6,5)}}{R_{\mathrm{dye},\mathrm{dye}}} = \dfrac{O^s_{\mathrm{dye},(6,5)} - A_{(6,5)}}{O^s_{\mathrm{dye},\mathrm{dye}} - A_{\mathrm{dye}}} \end{cases}$$

The values O^s and A are obtained from single-stained and non-stained cells, respectively. The measured parameter values and the resulting spillover coefficients are listed in Table S8.

Table S8. Parameter values for spillover calculation by median fluorescence intensity.

Parameter	$A_{(6,5)}$*	A_{dye}	$O^s_{(6,5),\mathrm{dye}}$	$O^s_{\mathrm{dye},\mathrm{dye}}$	$O^s_{\mathrm{dye},(6,5)}$	$O^s_{(6,5),(6,5)}$	$S_{\mathrm{dye},(6,5)}$	$S_{(6,5),\mathrm{dye}}$
By median								
PI	52.9	739.7	906.8	4,255.9	239.1	171.8	0.053	1.4054
DCF	84.6	1,022.4	1,020.6	65,916.4	128.3	193.4	0.0007	-0.0165
CRDR	85.0	744.4	744.6	28,352.6	134.4	176.8	0.002	0.0022
Hoechst	61.1	1,463.7	970.2	116,473.6	96	132.7	0.0003	-6.8925
By mean								
PI	203.5	1,035.9	1,367.2	8367.5	418.6	419.2	0.029	1.5361
DCF	202.9	1,641.1	1,637.7	136,745.7	234.7	500.1	0.0002	-0.0114
CRDR	196.7	1,012.4	1,010.4	34,061.3	232.6	462.01	0.00109	-0.00762
Hoechst	193.7	3,956.6	1,425.5	182,621.4	200.8	330.7	0.00004	-18.4862

* Note that the $A_{(6,5)}$ represents the autofluorescence of non-stained cells in the (6,5) channel. The four numbers in the column correspond to measurements of the same sample and the same channel. Slight variations among them arise from four different measurements conducted for the four dual-stained dye-(6,5) experiments.

Discussion S21. *Spillover compensations between dyes and (6,5) channels*

Figure S20 shows intensity scatter plots of dye (CRDR, DCF or Hoechst 33342) and (6,5) channels with and without spillover compensations. In the CRDR and DCF groups, the spillover coefficients for CRDR and (6,5) channels are only 0.2% and 0.01%, respectively, and therefore spillover compensation does not significantly alter the data pattern. In the DCF group, the negative spillover coefficient $S_{(6,5),\mathrm{DCF}}$ at -1.65% originates from the lower intensity value of (6,5) in the DCF channel $O^{S}_{(6,5),\mathrm{DCF}}$ compared to the cell autofluorescence intensity in the DCF channel A_{DCF} (see Table S8). This 1.8 difference might arise from measurement uncertainty.

However, a significant compensation (with a spillover coefficient $S_{(6,5),\mathrm{Ho}}$ of -6.8925) is required for the Hoechst 33342 channel intensity. This shifts the data population to the upper region and forms a clear diagonal line. We suspect that a significant reduction in the intensity of the target fluorophore leads to the large negative $S_{(6,5),\mathrm{Ho}}$. It has been reported that SWCNTs can quench fluorophores when adsorbed on the surface of the SWCNTs.[7] The intracellular (6,5) might interact with some specific innate fluorophores (e.g., flavin, NADPH, etc.) in cells and quench the fluorophore emission in 425-475 nm range. A more detailed study is required in the future.

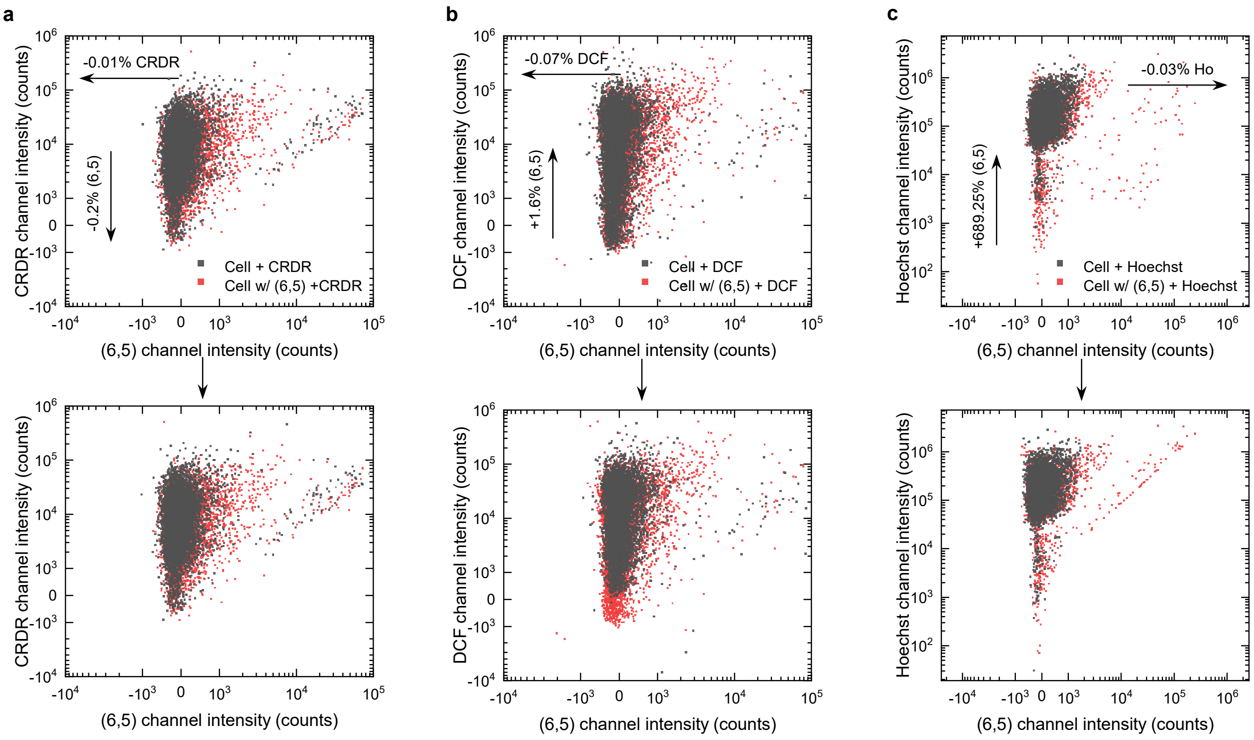

Figure S20. Spillover compensations between dyes (CRDR, DCF and Hoechst 33342) and (6,5). (a-c) Compensation of (a) CRDR, (b) DCF and (c) Hoechst 33342 groups, using the calculated spillover coefficients.

Discussion S22. <u>Investigation of the overcompensation in the case of PI-(6,5) system</u>

Figure S21 displays scatter plots of PI versus (6,5) channels before and after compensations using Eq 6 and data from Table S8. The compensation results, particularly on the PI channel, exhibit an unusual tail in the lower right of the population, suggesting potential over-compensation. Over-compensation occurs when cell conditions, such as size and complexity, differ among groups, typically between control and experimental groups. In such cases, manual adjustment of the spillover coefficients becomes necessary. We suspect that the increase in cell size and complexity after (6,5) treatment leads to an elevation in

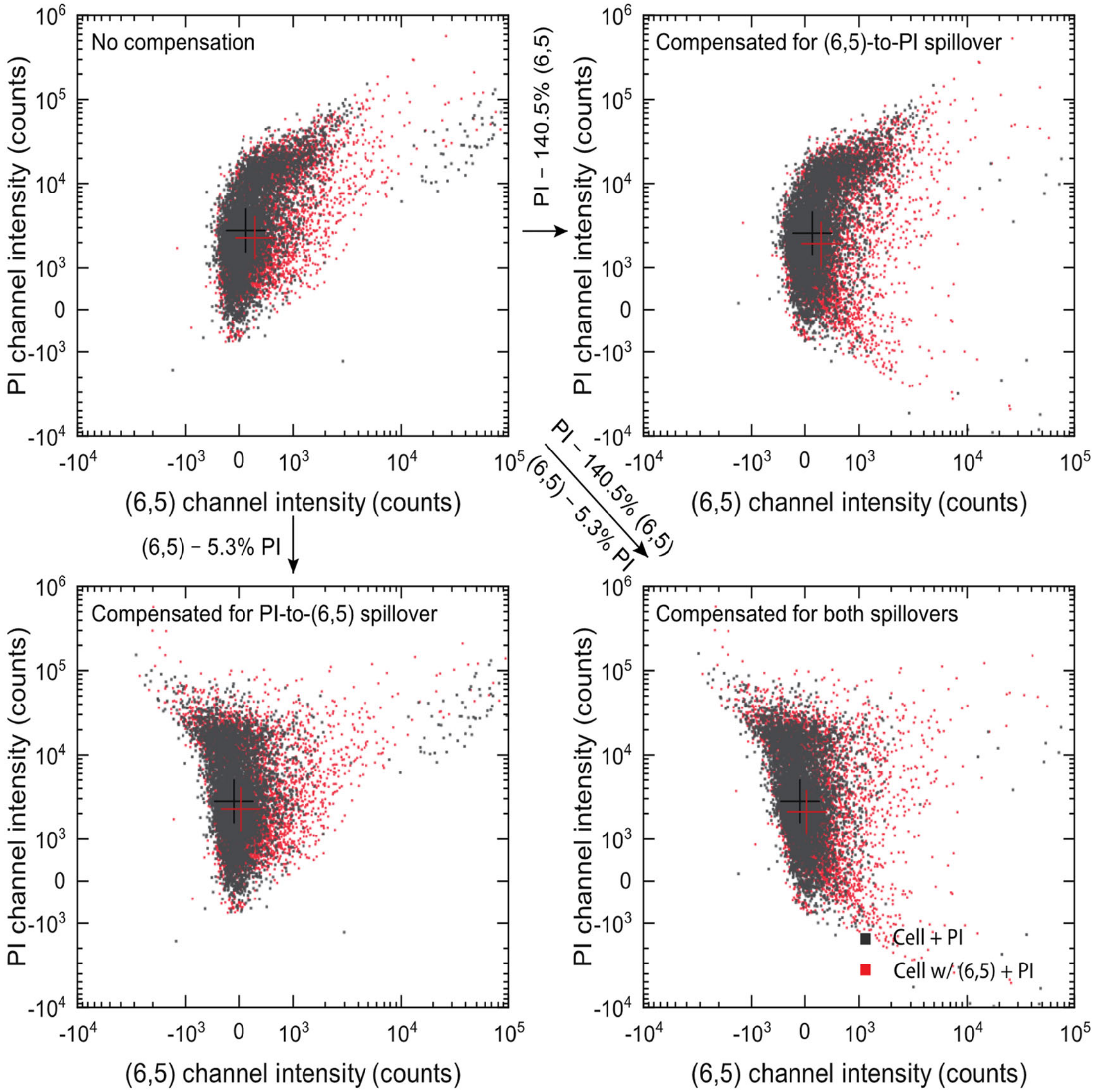

Figure S21. Scatter plot of PI versus (6,5) channel intensity for dual-stained and non-stained cell groups. The PI-to-(6,5) and (6,5)-to-PI spillover coefficients are calculated directly using Eq6 and are 5.3% and 140.5%, respectively.

autofluorescence levels, as depicted in Figure S22a. Figure S22b provides evidence of increasing forward scattering with increasing amount of intracellular (6,5).

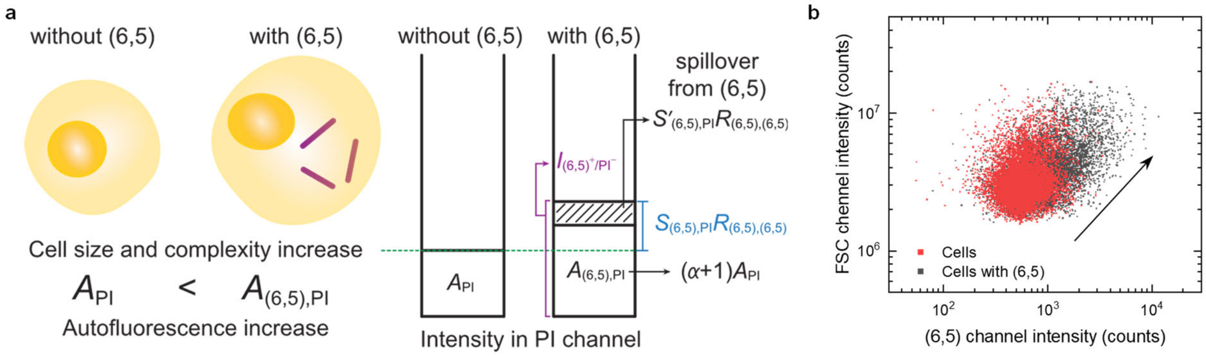

Figure S22. (a) Schematic illustration of the autofluorescence change and the relation of autofluorescence and (6,5) spill. (b) Scattering plot showing the relation between FSC and (6,5) channel intensities.

To estimate the increase of the autofluorescence level in the PI channel, we assume that the autofluorescence levels for PI-stained and dual-stained cells are denoted as A_{PI} and $A_{(6,5),\mathrm{PI}}$, respectively. The relationship between A_{PI} and $A_{(6,5),\mathrm{PI}}$ is defined by:

$$A_{(6,5),\mathrm{PI}} = (1+\alpha)A_{\mathrm{PI}} \tag{26}$$

where α is the autofluorescence enhancement factor. The (6,5)-to-PI spillover coefficient $S_{(6,5),\mathrm{PI}}$ is ~1.4054, as obtained from Table S8. In Figure S23, various 2D intensity distributions are depicted, incorporating manually adjusted spillover coefficients $S'_{(6,5),\mathrm{PI}}$ ranging from 0 to 1.4054. A more symmetric signal spreading is evident with $S'_{(6,5),\mathrm{PI}}$ set at 0.35, suggesting a more accurate representation of the distribution. Thus, the observed intensity of $(6,5)^+/\mathrm{PI}^-$ cells from the PI channel can be expressed as follows:

$$I_{(6,5)^+/\mathrm{PI}^-} = S_{(6,5),\mathrm{PI}}R_{(6,5),(6,5)} + A_{\mathrm{PI}} = S'_{(6,5),\mathrm{PI}}R_{(6,5),(6,5)} + A_{(6,5),\mathrm{PI}} \tag{27}$$

Combine the two equations above:

$$S_{(6,5),\mathrm{PI}}R_{(6,5),(6,5)} + A_{\mathrm{PI}} = S'_{(6,5),\mathrm{PI}}R_{(6,5),(6,5)} + (\alpha+1)A_{\mathrm{PI}} \tag{28}$$

Rearranging the equation gives

$$\alpha = \left(S_{(6,5),\mathrm{PI}} - S'_{(6,5),\mathrm{PI}}\right) \times \frac{R_{(6,5),(6,5)}}{A_{\mathrm{PI}}} = \left(S_{(6,5),\mathrm{PI}} - S'_{(6,5),\mathrm{PI}}\right) \times \frac{O^S_{(6,5),(6,5)} - A_{(6,5)}}{A_{\mathrm{PI}}} \tag{29}$$

Apply the following values into the equation:

$$S_{(6,5),\mathrm{PI}} = 1.4054;\ S'_{(6,5),\mathrm{PI}} = 0.35;\ O^S_{(6,5),(6,5)} = 171.8;\ A_{(6,5)} = 52.9;\ A_{\mathrm{PI}} = 739.7$$

Then

$$\alpha = (1.4054 - 0.35) \times \frac{171.8 - 52.9}{739.7} = 0.17$$

The outcome suggests that the autofluorescence level of the cells increases by ~17% following (6,5) treatment. This increase introduces an overcompensation issue since the initial assumption of identical autofluorescence levels proves incorrect.

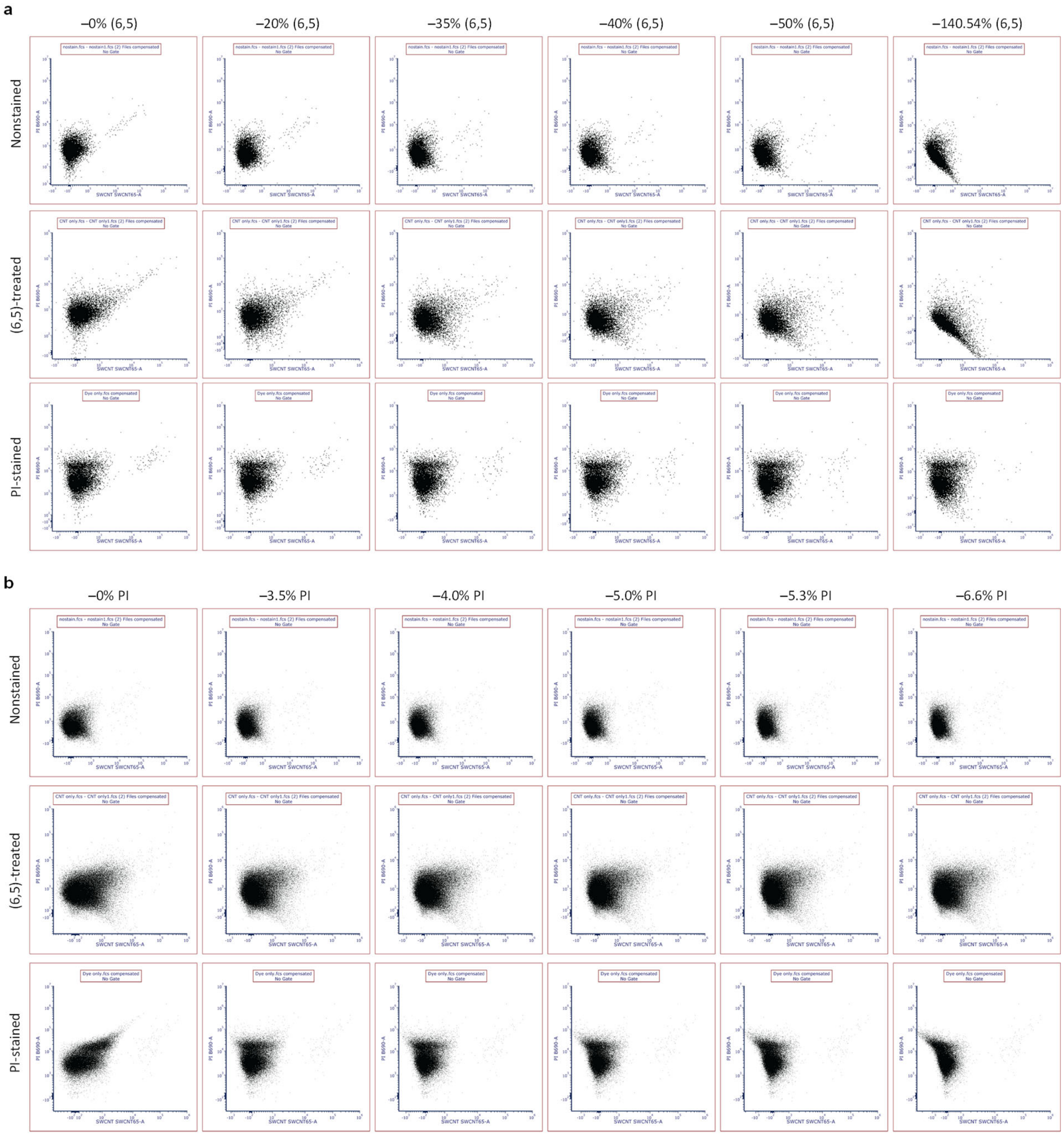

Figure S23. Scatters plot of PI to (6,5) channel intensity with respect to various spillover coefficient values for compensations. (a) Compensation for (6,5)-to-PI spillover. The value 0.35 is chosen instead of 1.4054. (b) Compensation for PI-to-(6,5) spillover. The value 0.05 is chosen instead of 0.053.

Assuming that the spectral shape of autofluorescence remains constant after (6,5) treatment, we can determine the manually adjusted PI-to-(6,5) spillover coefficient $S'_{\mathrm{PI},(6,5)}$, using the following equation:

$$A_{(6,5),(6,5)} = (1+\alpha)A_{(6,5)} \tag{30}$$

$$I_{(6,5)^+} = S_{\mathrm{PI},(6,5)}R_{\mathrm{PI,PI}} + A_{(6,5)} = S'_{\mathrm{PI},(6,5)}R_{\mathrm{PI,PI}} + A_{(6,5),(6,5)} \tag{31}$$

Combining and rearranging the above two equations gives

$$S'_{\mathrm{PI},(6,5)} = S_{\mathrm{PI},(6,5)} - \frac{\alpha A_{(6,5)}}{O^{s}_{\mathrm{PI,PI}} - A_{\mathrm{PI}}} \tag{32}$$

All the values can be found in Table S8:

$$S_{\mathrm{PI},(6,5)} = 0.053;\ \alpha = 0.17;\ A_{(6,5)} = 52.9;\ O^{s}_{\mathrm{PI,PI}} = 4{,}255.9;\ A_{\mathrm{PI}} = 739.7$$

Placing the values into the equation gives

$$S'_{\mathrm{PI},(6,5)} = 0.053 - \frac{0.17 \times 52.9}{4255.9 - 739.7} = 0.05044$$

Discussion S23. <u>The relation of cellular (6,5) mass and cellular response using SWIR flow cytometry</u>

Figure S24a-d shows scatter plots of CRDR or FSC channel intensity versus (6,5) channel intensity for the remaining samples not displayed in Figure 6. The thresholds for CRDR and (6,5) channel intensities are set at the top 1% signals, which are Q1 + Q4 and Q1 + Q2, respectively, of the non-stained control sample, while the threshold for FSC channel intensity is determined at the intersection point of FSC signals between non-treated and LPS-treated cell groups. This rule applies to Figure 5 as well.

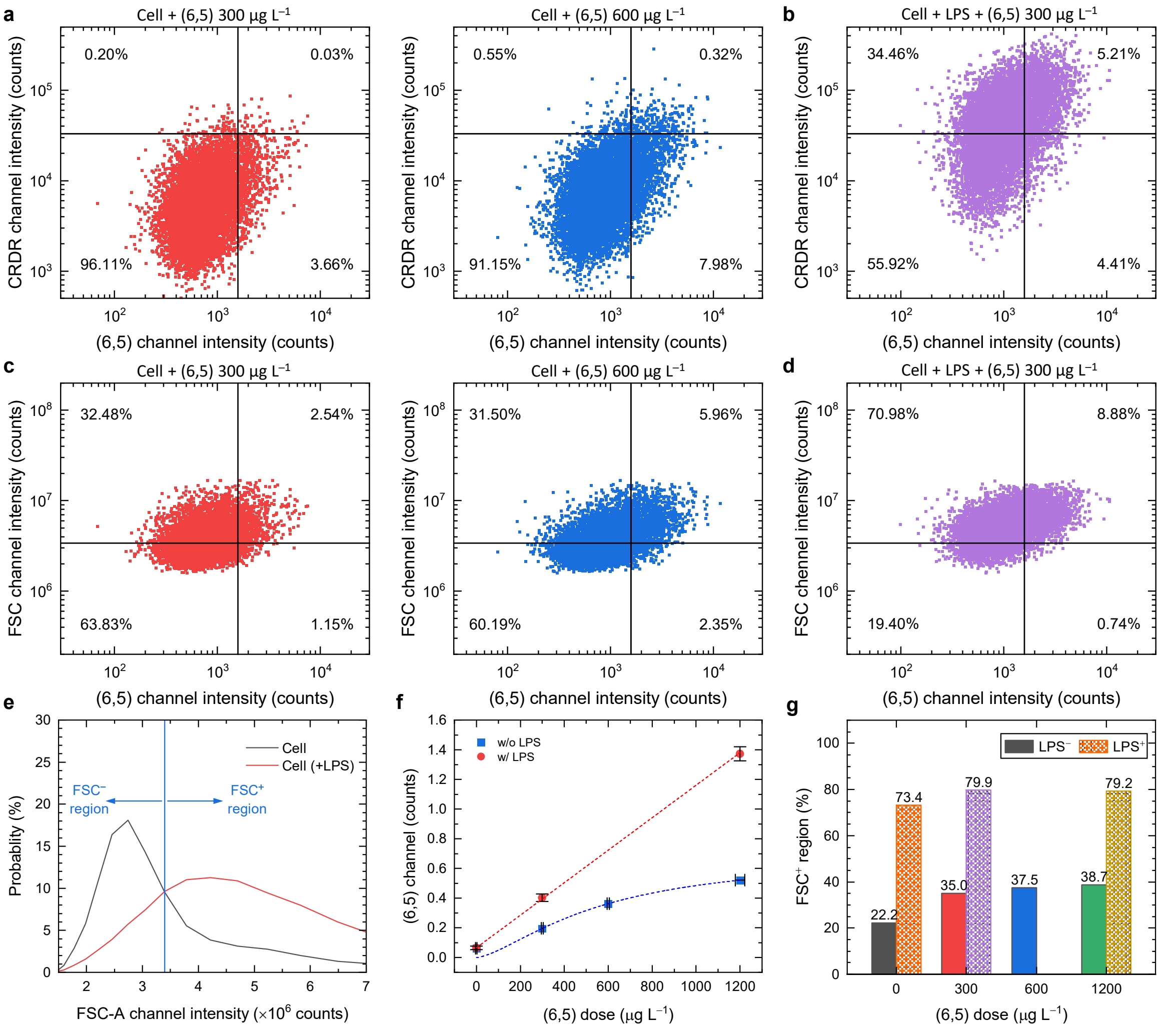

Figure S24. The correlation between cellular (6,5) mass and ROS generation. (a-d) Scatter plots of (a, b) CRDR or (c, d) FSC intensity versus (6,5) channel intensity. (e) Probability histogram of FSC-A channel intensity. The boundary for FSC^{+} and FSC^{-} regions are determined by the intersection point of non-treated and LPS-treated cell groups. (f) (6,5) channel intensity with respect to treated (6,5) concentration. Short dashed lines are guides to the data trends, fitted with a linear (red) and a Hill (blue) function for LPS- and non-treated samples, respectively. Different LPS effects on cellular (6,5) uptake can be clearly observed. The error bars represent SD. Experiments were conducted in triplicates, and around 30-40 thousand cells were collected in each experimental group. Results are mean ± SD obtained from triplicate experiments.

The flow cytometry allows for the evaluation of another cell characteristic, namely, cell complexity or granularity, through side scattering (SCC). We further analyze the relationship between SSC and (6,5)

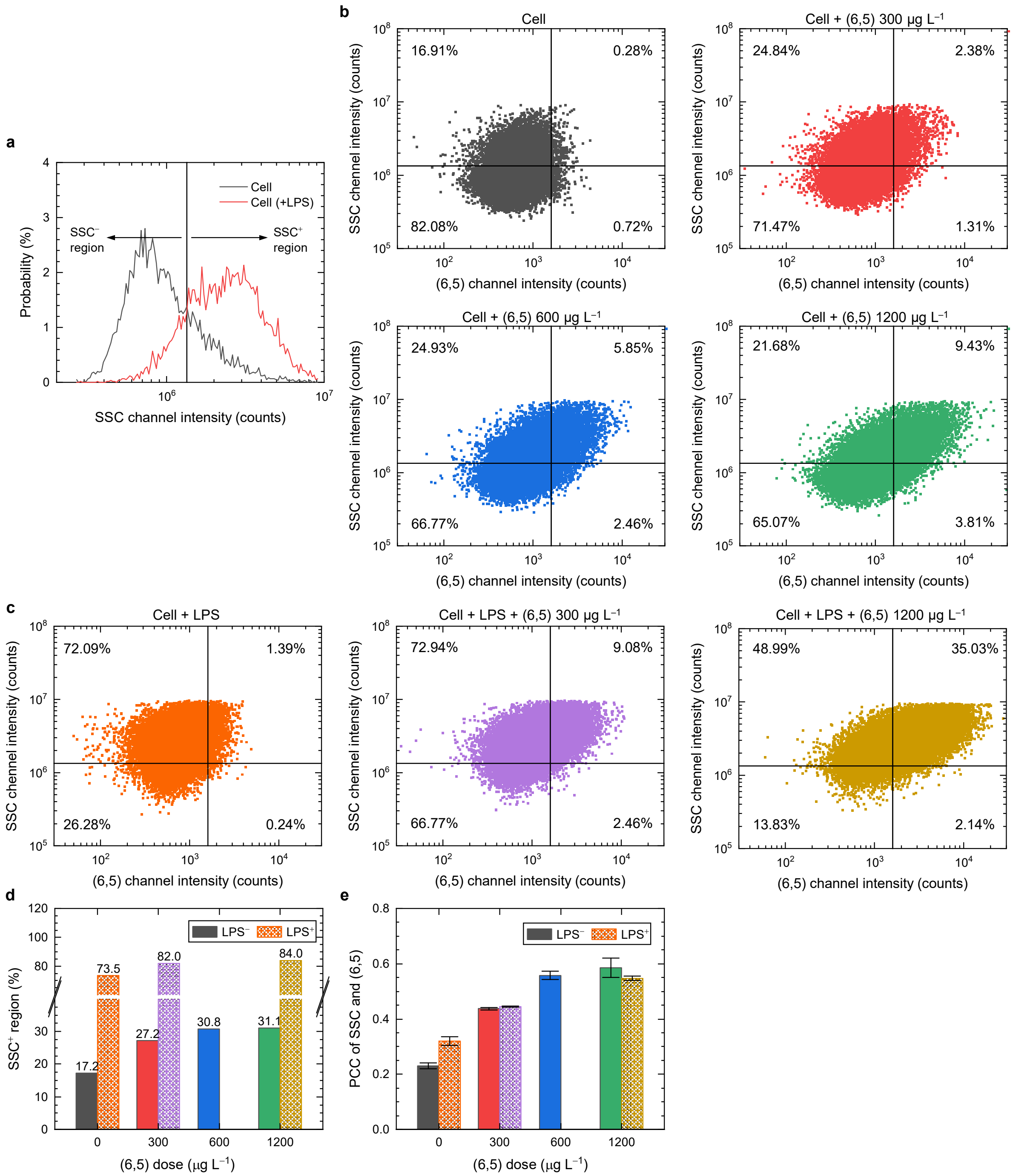

Figure S25. The correlation between cellular (6,5) mass and cell complexity. (a) Probability histogram of SSC-A channel intensity. The boundary for SSC⁺ and SSC⁻ regions is determined by the intersection point of non-treated and LPS-treated cell groups. (b, c) Scatter plots of SSC versus (6,5) channel intensity from (b) non-treated and (c) LPS-treated cells with various (6,5) doses. (d) SSC⁺ region with various (6,5) doses. e PCC of SSC and (6,5). The error bars represent SD. Experiments were conducted in triplicates, and around 30-40 thousand cells were collected in each experimental group. Results are mean ± SD obtained from triplicate experiments.

channel intensities. Similar to the previous case, the threshold for SSC^+ and SSC^- groups is set at the intersection point of FSC signals between non-treated and LPS-treated cell groups (see Figure S25a). The LPS treatment shifts ~56% of the cell population from SSC^- to SSC^+ region, indicating an increase of cytoplasmic granularity in proinflammatory macrophages (see Figure S25b-d).[8] Additionally, an ~0.6% increase in $(6,5)^+_{ch}$ is observed, suggesting that cell complexity also contributes to the autofluorescence in the SWIR. The elevated PCC between SSC and (6,5) channel intensities with increased (6,5) dose is consistent with the results in the CRDR and FSC cases described in the main text (Figure S25e), indicating that (6,5) could induce oxidative stress and macrophage activation.

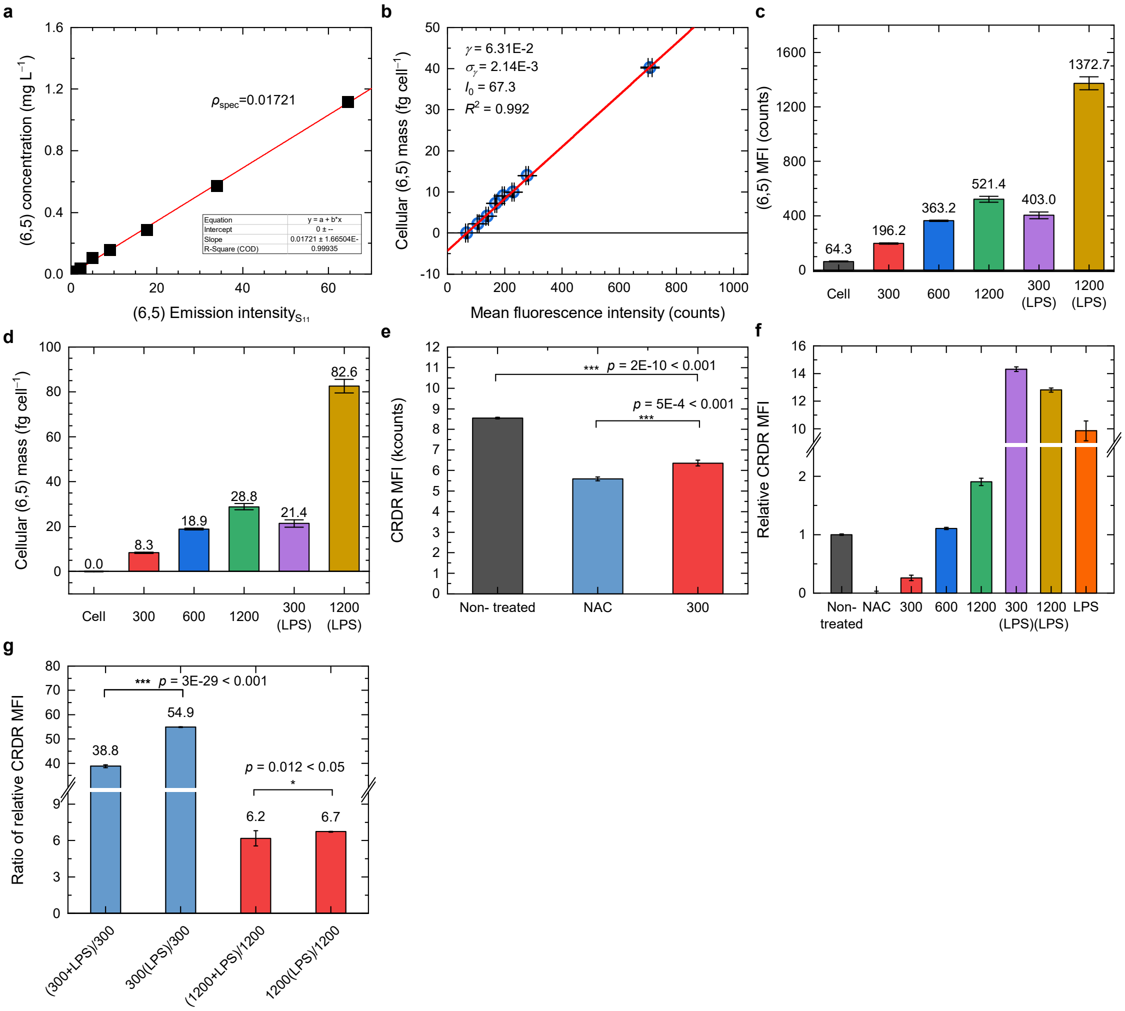

Figure S26. Quantification of (6,5) in RAW 264.7 from the experiment shown in Figure 5. (a) Calibration of the relationship between (6,5) concentration and fluorescence peak intensity. (b) Cellular (6,5) mass as a function of the MFI. (c) MFI of (6,5) with respect to the (6,5) dose. (d) Cellular (6,5) mass with respect to the (6,5) dose. (e) Comparison of CRDR MFIs from non-treated, NAC-treated and 300 µg L^{-1} (6,5)-treated cells. The statistical significances between groups are tested and represented as p values. ***p < 0.001 (unpaired two-tailed t-test). (f) Relative CRDR MFI with respect to the treatment conditions. (g) Ratio of relative CRDR MFI normalized by non-LPS treated group. ***p < 0.001 and *p < 0.05 from unpaired two-tailed t-test.

The quantification of (6,5) in the cells can be further performed using our developed quantification protocol. The experimental cell samples for this experiment were divided into two aliquots for flow cytometry and bulk spectrometry measurements. The ρ_{spec} is obtained from the measurements of absorption and emission spectra using stock (6,5) materials (Figure S26a). Combining this result with the cytometry data, the correlation between cellular (6,5) mass $\bar{m}$ and (6,5) MFI $\bar{I}$ can be deduced (Figure S26b). This correlation allows for the conversion of the MFI obtained from samples to the cellular (6,5) mass (Figure S26c,d). We note that the LPS treatment increases (6,5) uptake approximately threefold, from 8.3 to 21.4 fg cell^{-1} under the 300 μg L^{-1} dose condition. Figure S26e evaluates the significance of the differences between the non-treated/NAC and 300 μg L^{-1} groups. In both cases, significant differences are observed, indicating that the antioxidation effect is real and that the 300 μg L^{-1} dose has not reached the minimum possible ROS level. Next, we use NAC-treated cells (NAC) as the background in the CRDR channel and employ non-treated cells (Cell) as a reference. The relative CRDR levels for all other conditions are depicted in Figure S26f. From this, we conclude that the (6,5)-induced ROS does not exceed two times the original ROS level, while the LPS-induced ROS reaches almost ten times. In other words, LPS induces significantly higher ROS stimulation compared to (6,5). Moreover, the combination of LPS and (6,5) treatments appears to enhance ROS production even more than the combination of the two individual treatments, as shown in Figure S26g.

Discussion S24. The correlation of cell size and cell autofluorescence

The relationship between cell size and autofluorescence can be discerned from the results presented in Figure 5d, Figure S9a and Figure S22. Figure 5d shows an increased PCC after LPS treatment at zero (6,5) dose, indicating a correlation between cell size and autofluorescence. Figure S9 shows a positive correlation (PCC value of ~0.2) between cell autofluorescence in the (6,5) channel and FSC intensity (cell size). Additionally, Figure S22 indicates a ~17% increase in autofluorescence, along with the observed increased FSC intensity of macrophages after (6,5) treatment. These suggest a positive correlation between autofluorescence and cell size, supported by similar findings in the literature.[9] Previous studies have demonstrated a positive correlation between cell size, granularity, and autofluorescence, particularly in mesenchymal stromal cells. Lipofuscin, an autofluorescence-associated protein, accumulates in older cells with larger sizes and lysosomal masses. Hence, macrophages likely exhibit a similar positive correlation between autofluorescence and cell size. However, further investigations are necessary to elucidate the exact mechanism. No correlation between cell viability and autofluorescence was observed in our study.

Discussion S25. *Evaluation of the cytotoxicity of $(GT)_{20}$-coated (6,5) SWCNTs*

The cytotoxicity of $(GT)_{20}$-coated (6,5) was evaluated by PrestoBlue HS assay (P50200, Invitrogen™). The cell viability in all interested (6,5) doses are close to 100%, indicating no toxicity concerns of using $(GT)_{20}$-coated (6,5).

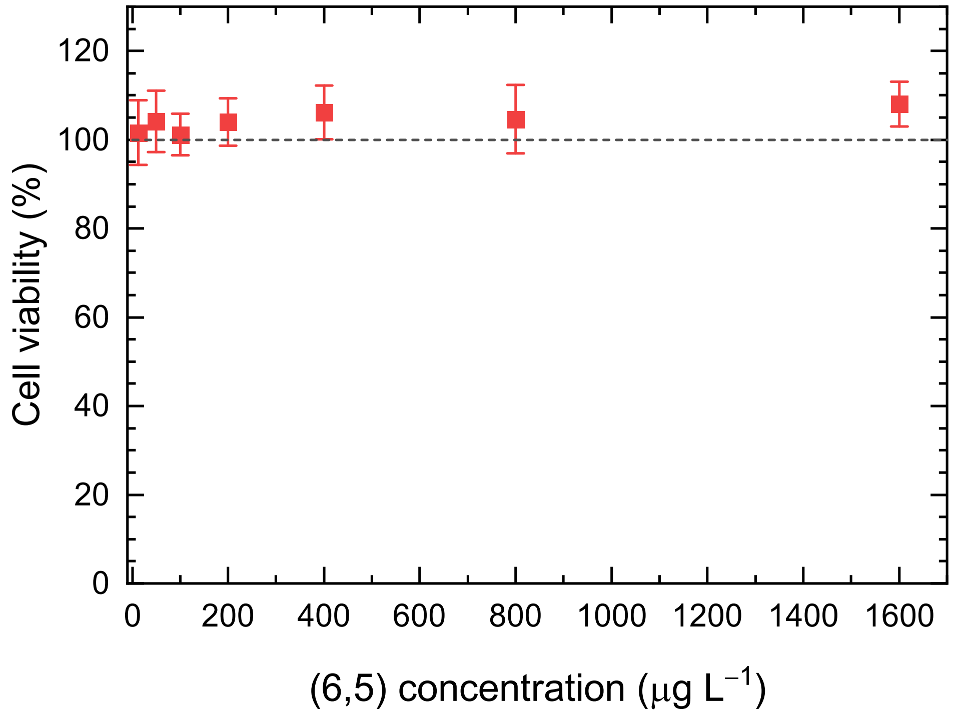

Figure S27. Cytotoxicity assessment of $(GT)_{20}$-coated (6,5) incubated with RAW 264.7 using PrestoBlue HS assay. Error bar represents the mean ± SD from triplicate experiments.

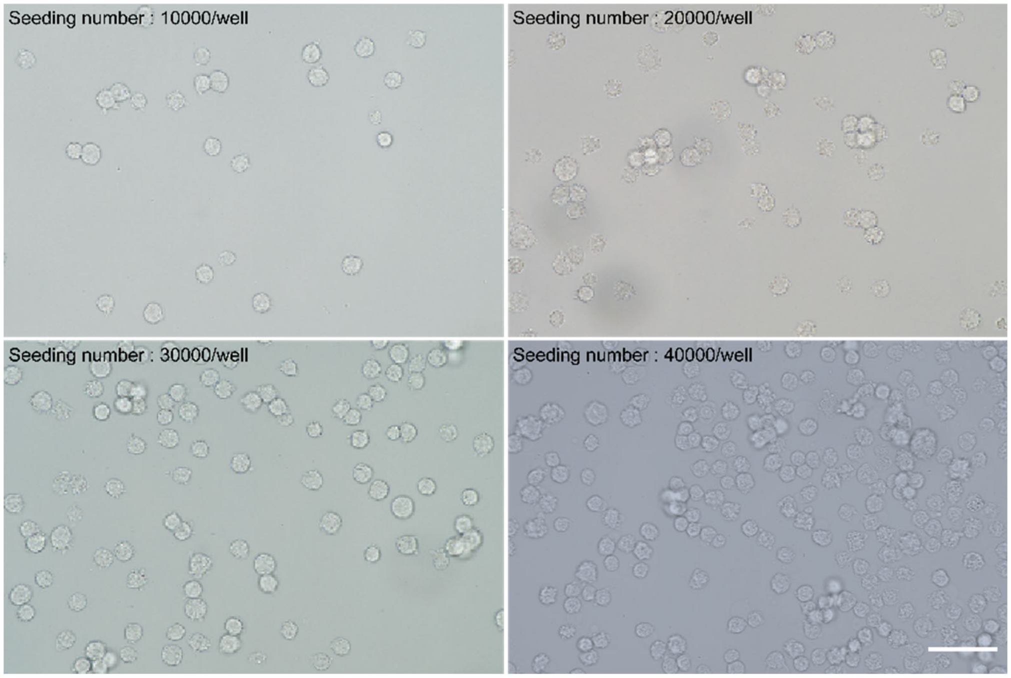

Figure S28. Photographs of RAW 264.7 with different cell densities in 8-well chambered coverglass. Scale bar is 50 µm.

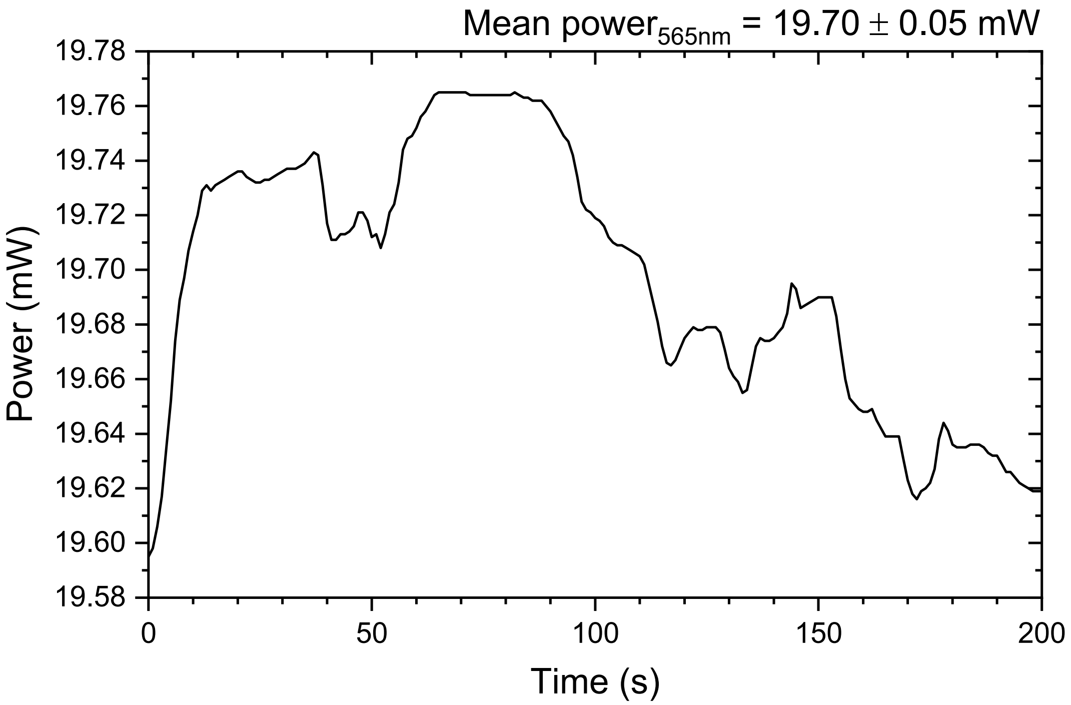

Figure S29. The mean power intensity of 565 nm LED light source from Thorlabs CHROLIS measured directly at the focal plane of the 20× objective.

References

(1) Li, H.; Gordeev, G.; Garrity, O.; Peyyety, N. A.; Selvasundaram, P. B.; Dehm, S.; Krupke, R.; Cambré, S.; Wenseleers, W.; Reich, S.; Zheng, M.; Fagan, J. A.; Flavel, B. S. Separation of Specific Single-Enantiomer Single-Wall Carbon Nanotubes in the Large-Diameter Regime. *ACS Nano* **2020**, *14*, 948-963.
(2) Chantzoura, E.; Kaji, K. Chapter 10 - Flow Cytometry. In *Basic Science Methods for Clinical Researchers,* Jalali, M., Saldanha, F. Y. L., Jalali, M. Eds.; Academic Press, 2017; pp 173-189.
(3) Montes-Pérez, J. J.; Moreno-Ostos, E.; Marañón, E.; Blanco, J. M.; Rodríguez, V.; Rodríguez, J. Intermediate-size cell dominance in the phytoplankton community of an eutrophic, estuarine ecosystem (Guadalhorce River, Southern Spain). *Hydrobiologia* **2020**, *847*, 2241-2254.
(4) Jeng, E. S.; Moll, A. E.; Roy, A. C.; Gastala, J. B.; Strano, M. S. Detection of DNA Hybridization using the Near-Infrared Band-Gap Fluorescence of Single-Walled Carbon Nanotubes. *Nano Lett.* **2006**, *6*, 371-375.
(5) Wei, X.; Tanaka, T.; Li, S.; Tsuzuki, M.; Wang, G.; Yao, Z.; Li, L.; Yomogida, Y.; Hirano, A.; Liu, H.; Kataura, H. Photoluminescence Quantum Yield of Single-Wall Carbon Nanotubes Corrected for the Photon Reabsorption Effect. *Nano Lett.* **2020**, *20*, 410-417.
(6) Sanchez, S. R.; Bachilo, S. M.; Kadria-Vili, Y.; Lin, C. W.; Weisman, R. B. (*n,m*)-Specific Absorption Cross Sections of Single-Walled Carbon Nanotubes Measured by Variance Spectroscopy. *Nano Lett.* **2016**, *16*, 6903-6909.
(7) Zhu, Z.; Yang, R.; You, M.; Zhang, X.; Wu, Y.; Tan, W. Single-Walled Carbon Nanotube as an Effective Quencher. *Anal. Bioanal. Chem.* **2010**, *396*, 73-83.
(8) Nasra, S.; Shah, T.; Bhatt, M.; Chaudhari, R.; Bhatia, D.; Kumar, A. Reprogramming M1-to-M2 Phenotype to Alleviate Inflammation: using Liposomal Curcumin as a Tool to Redefine Macrophage Functionality. *ACS Appl. Bio Mater.* **2023**, *6*, 2886-2897.
(9) Bertolo, A.; Baur, M.; Guerrero, J.; Pötzel, T.; Stoyanov, J. Autofluorescence is a Reliable In Vitro Marker of Cellular Senescence in Human Mesenchymal Stromal Cells. *Sci. Rep.* **2019**, *9*, 2074.
